# Defects in mitophagy promote redox-driven metabolic syndrome in the absence of TP53INP1

**DOI:** 10.15252/emmm.201404318

**Published:** 2015-03-31

**Authors:** Marion Seillier, Laurent Pouyet, Prudence N'Guessan, Marie Nollet, Florence Capo, Fabienne Guillaumond, Laure Peyta, Jean-François Dumas, Annie Varrault, Gyslaine Bertrand, Stéphanie Bonnafous, Albert Tran, Gargi Meur, Piero Marchetti, Magalie A Ravier, Stéphane Dalle, Philippe Gual, Dany Muller, Guy A Rutter, Stéphane Servais, Juan L Iovanna, Alice Carrier

**Affiliations:** 1 Inserm, U1068, CRCMMarseille, France; 2 Institut Paoli-CalmettesMarseille, France; 3 Aix-Marseille UniversitéMarseille, France; 4 CNRS, UMR7258, CRCMMarseille, France; 5 Inserm, U1069, Nutrition, Croissance et Cancer (N2C)Tours, France; 6 CNRS, UMR5203, Inserm, U661, Universités de Montpellier 1 & 2, IGFMontpellier, France; 7 Inserm, U1065, C3M, Team 8 “Hepatic Complications in Obesity”Nice, France; 8 Université de Nice-Sophia-AntipolisNice, France; 9 Centre Hospitalier Universitaire de Nice, Pôle Digestif, Hôpital L'ArchetNice, France; 10 Cell Biology, Department of Medicine, Imperial CollegeLondon, UK; 11 Islet Cell Laboratory, University of Pisa – Cisanello HospitalPisa, Italy

**Keywords:** autophagy, diabetes, mitochondria, obesity, oxidative stress

## Abstract

The metabolic syndrome covers metabolic abnormalities including obesity and type 2 diabetes (T2D). T2D is characterized by insulin resistance resulting from both environmental and genetic factors. A genome-wide association study (GWAS) published in 2010 identified *TP53INP1* as a new T2D susceptibility locus, but a pathological mechanism was not identified. In this work, we show that mice lacking TP53INP1 are prone to redox-driven obesity and insulin resistance. Furthermore, we demonstrate that the reactive oxygen species increase in TP53INP1-deficient cells results from accumulation of defective mitochondria associated with impaired PINK/PARKIN mitophagy. This chronic oxidative stress also favors accumulation of lipid droplets. Taken together, our data provide evidence that the GWAS-identified *TP53INP1* gene prevents metabolic syndrome, through a mechanism involving prevention of oxidative stress by mitochondrial homeostasis regulation. In conclusion, this study highlights TP53INP1 as a molecular regulator of redox-driven metabolic syndrome and provides a new preclinical mouse model for metabolic syndrome clinical research.

## Introduction

Metabolic syndrome (MS) describes a cluster of metabolic abnormalities including obesity, insulin resistance, hypertension and dyslipidemia (Pothiwala *et al*, 2009). The prevalence of MS has been increasing exponentially in the last few decades, paralleling the obesity epidemic. Obesity, which is defined as a body mass index ≥ 30 kg/m^2^, results from accumulation of white adipose tissue. It depends on both genetic and environmental factors, in particular lifestyles featuring increased nutrient caloric intake but decreased calorie consumption. Diet may have a major role in the pathogenesis and prevalence of obesity (Calder *et al*, 2011), but other causes have to be considered, such as gut microbiota which affect host nutritional metabolism (Musso *et al*, 2010; Greiner & Backhed, 2011), and lack of physical exercise (Calder *et al*, 2011). Excess body weight is a major public health concern since it is associated with increased risk of cardiovascular disease, type 2 diabetes (T2D), Alzheimer's disease and cancer (van Kruijsdijk *et al*, 2009; Siegel & Zhu, 2009; Forte *et al*, 2012; Leboucher *et al*, 2013).

One major link between obesity and associated diseases is the chronic low-grade inflammatory state observed in obese patients (Calder *et al*, 2011; Gregor & Hotamisligil, 2011). Inflammation is induced by excessive accumulation of lipids in adipose tissue leading to adipocyte stress and release of inflammatory cytokines and adipokines. The resulting recruitment of immune cells to key metabolic organs further contributes to chronic inflammation. Obesity-associated immune signals concern all types of immune cells that prompt inflammation, as well as adipocytes themselves (Chawla *et al*, 2011; Deng *et al*, 2013). Importantly, the obesity-associated chronic low-grade inflammatory state impacts all organs in the body. Hence, obese patients are at increased risk of developing cancer in any localization even if pancreatic and liver cancers show the highest increase in risk (Siegel & Zhu, 2009).

Inflammation is associated with oxidative stress which is one obesity-related feature participating in the development of MS (Bondia-Pons *et al*, 2012; Khoo *et al*, 2012; Rolo *et al*, 2012; Crujeiras *et al*, 2013). Oxidative stress results from excess of reactive oxygen species (ROS) production overwhelming antioxidant defenses (Pouyet & Carrier, 2010). ROS are mainly produced as by-products of the mitochondrial electron transport chain involved in ATP production (oxidative phosphorylation). Excess fatty acids and glucose are known to be deleterious for mitochondrial function, thus increasing ROS production. ROS can oxidize cell macromolecules, leading to impaired cellular homeostasis and associated pathologies such as cancer (Gupta *et al*, 2012; Crujeiras *et al*, 2013).

In the recent years, we have provided evidence that tumor protein 53-induced nuclear protein 1 (TP53INP1) is a key stress protein with antioxidant-associated tumor suppressive function (Gironella *et al*, 2007; Gommeaux *et al*, 2007; Cano *et al*, 2009; N'Guessan *et al*, 2011; Seux *et al*, 2011). The *TP53INP1* gene (a transcriptional target of p53 and other transcription factors) is highly conserved between human and rodents and over-expressed during stress response including inflammation (Tomasini *et al*, 2001; Jiang *et al*, 2004). TP53INP1-deficient mice, which lack participation of TP53INP1 in stress resolution, are prone to stress-induced dysfunctions including cancer (Gironella *et al*, 2007; Gommeaux *et al*, 2007; Cano *et al*, 2009; N'Guessan *et al*, 2011). Moreover, these mutant mice show a chronic oxidative stress characterized by an increase in the cell ROS level as well as a decrease of antioxidant defenses (Gommeaux *et al*, 2007; Cano *et al*, 2009; N'Guessan *et al*, 2011). Restoration of TP53INP1 expression in TP53INP1-deficient cells rescues the phenotype by alleviating ROS burden (Cano *et al*, 2009).

We demonstrated that TP53INP1 impacts on p53 and p73 transcriptional activity by direct interaction and mediates the antioxidant activity of p53 (Tomasini *et al*, 2003, 2005; Cano *et al*, 2009). The tumor suppressor p53 is a fascinating protein endowed with multiple functions, including metabolic regulation, in common with two other members of this family: p63 and p73 (Maddocks & Vousden, 2011; Rufini *et al*, 2012; Su *et al*, 2012; Liang *et al*, 2013). We also provided evidence for a role of TP53INP1 in autophagy by direct interaction with mammalian Atg8 orthologs including LC3 (Seillier *et al*, 2012). Autophagy is a catabolic process involved in the cellular energetic balance and lipid homeostasis thus regulating obesity (Singh & Cuervo, 2011; Lavallard *et al*, 2012). Interestingly, a genome-wide association study (GWAS) published in 2010 identified *TP53INP1* as a new T2D susceptibility locus (Voight *et al*, 2010). Collectively, those observations led us to address the role of TP53INP1 in metabolic regulation. We used TP53INP1-deficient mice to assess *in vivo* the effect of a high-fat diet which favors obesity, insulin resistance and T2D, and we investigated the cellular metabolic defects induced by TP53INP1 deficiency. In this work, we provide the demonstration that TP53INP1 is a primary molecular link between oxidative stress and MS.

## Results

### Absence of TP53INP1 favors obesity in a redox-dependent manner *in vivo*

**Figure 1:**
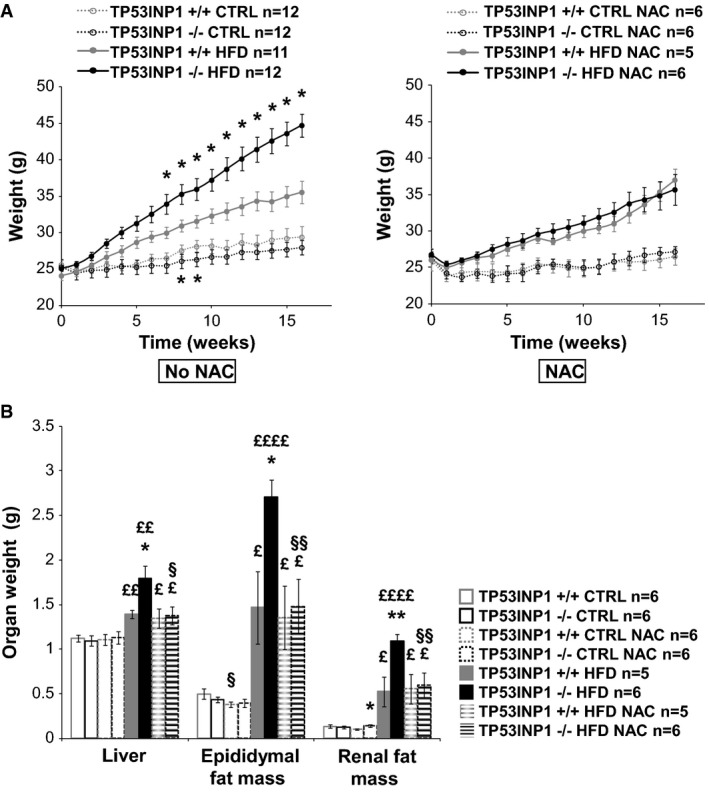
TP53INP1-deficient mice are highly susceptible to HFD-induced obesity owing to their chronic oxidative stress TP53INP1-KO (−/−) and WT (+/+) male mice were subjected to a high-fat diet (HFD, 60% fat) or a control diet (CTRL) for 16 weeks. Mice drank tap water or tap water supplemented with NAC (10 mg/ml or 1%).

We initially observed that the body weight of 5-month-old TP53INP1-deficient (KO or −/−) mice was higher than WT (+/+) in both males and females (Supplementary Fig S1A) and that fat mass was more abundant in TP53INP1-deficient than in WT mice (Supplementary Fig S1B). We supplemented drinking water with the anti-oxidant *N*-acetylcysteine (NAC) which alleviates chronic oxidative stress associated with TP53INP1 deficiency through increase of intracellular glutathione level (Cano *et al*, 2009; N'Guessan *et al*, 2011). NAC supplementation completely abolished fat mass difference between TP53INP1-deficient and WT mice (Supplementary Fig S1A and B). We then fed 8-week-old mice with a high-fat diet (HFD, 60% fat) (control (CTRL) food is 10% fat) during 16 weeks. We observed a higher body weight gain in HFD-fed TP53INP1 KO than WT mice (Fig1A and Supplementary Fig S2A), although food consumption did not differ between genotypes (Supplementary Fig S3). Epididymal and renal fat masses were higher in KO than in WT mice upon HFD (Fig1B and Supplementary Fig S2A). HFD-induced liver weight increase was also higher in HFD-fed TP53INP1 KO mice than WT (Fig1B and Supplementary Fig S2A). HFD-induced steatosis (accumulation of lipid droplets in hepatocytes), assessed by histological analysis, was greater in KO than in WT mice (Supplementary Fig S2B). Taken together, those data show that TP53INP1-deficient mice are prone to obesity and liver complications, suggesting a role of TP53INP1 in dampening fat storage. Interestingly, the gene encoding TP53INP1 was over-expressed in the liver of HFD-fed C57BL/6 mice (Supplementary Fig S2C). Furthermore, in human, morbidly obese patients with hepatic steatosis showed an increase in hepatic *TP53INP1* expression, and *TP53INP1* expression was correlated with the level of a marker of hepatocyte death (keratin 18), with the grade of steatosis and with the expression level of the stress marker NQO1 (Supplementary Fig S2D–H and Supplementary Table S2). This suggests that *TP53INP1* expression is induced as part of an obesity-associated stress response and that this protective function is lacking in TP53INP1-deficient mice, thus impairing fat homeostasis.

Curves show mice body weight recorded every week. CTRL: *P* (−/− versus +/+; *t* = 8w) = 0.047; *P* (−/− versus +/+; *t* = 9w) = 0.023. HFD: *P* (−/− versus +/+; *t* = 7w) = 0.039; *P* (−/− versus +/+; *t* = 8w) = 0.029; *P* (−/− versus +/+; *t* = 9w) = 0.021; *P* (−/− versus +/+; *t* = 10w) = 0.014; *P* (−/− versus +/+; *t* = 11w) = 0.0046; *P* (−/− versus +/+; *t* = 12w) = 0.0028; *P* (−/− versus +/+; *t* = 13w) = 0.0025; *P* (−/− versus +/+; *t* = 14w) = 0.00051; *P* (−/− versus +/+; *t* = 15w) = 0.00027; *P* (−/− versus +/+; *t* = 16w) = 0.00013.

At the end of protocol, mice were sacrificed; liver and epididymal and renal fat masses were taken and weighed. Histograms show organ weight. Liver: *P* (−/− versus +/+; HFD) = 0.014; *P* (HFD versus CTRL; +/+) = 0.00063; *P* (HFD versus CTRL; −/−) = 0.0010; *P* (HFD versus CTRL; +/+ NAC) = 0.034; *P* (HFD versus CTRL; −/− NAC) = 0.027; *P* (NAC versus no NAC; −/− HFD) = 0.014. Epididymal fat mass: *P* (−/− versus +/+; HFD) = 0.011; *P* (HFD versus CTRL; +/+) = 0.028; *P* (HFD versus CTRL; −/−) = 0.000017; *P* (HFD versus CTRL; +/+ NAC) = 0.019; *P* (HFD versus CTRL; −/− NAC) = 0.0054; *P* (NAC versus no NAC; +/+ CTRL) = 0.037; *P* (NAC versus no NAC; −/− HFD) = 0.0025. Renal fat mass: *P* (−/− versus +/+; HFD) = 0.0041; *P* (HFD versus CTRL; +/+) = 0.028; *P* (HFD versus CTRL; −/−) = 0.000013; *P* (HFD versus CTRL; +/+ NAC) = 0.019; *P* (HFD versus CTRL; −/− NAC) = 0.0078; *P* (NAC versus no NAC; −/− HFD) = 0.0047.

Data information: Results are expressed as the mean ± SEM and are representative of two independent experiments. * −/− versus +/+; ^£^ HFD versus CTRL; ^§^ NAC versus no NAC; 1 character: *P* < 0.05; 2 characters: *P* < 0.005; 4 characters: *P* < 0.00005.

In order to evaluate the impact of chronic oxidative stress in obesity predisposition of TP53INP1 KO mice, we treated the mice with NAC at the starting of HFD. Whereas NAC treatment did not modify final weight gain in HFD-fed WT mice, it completely abolished all body weight, organ weight and hepatic steatosis differences between HFD-fed KO and WT mice, bringing the KO mice values to those of the WT (Fig1 and Supplementary Fig S2B). These results illustrate that chronic oxidative stress affecting the TP53INP1-deficient mice predisposes them to increased weight gain and adiposity, further favoring obesity and hepatic steatosis when challenged with a lipid-rich diet.

### Insulin resistance establishment is elicited by chronic oxidative stress induced by TP53INP1 deficiency *in vivo*

**Figure 2:**
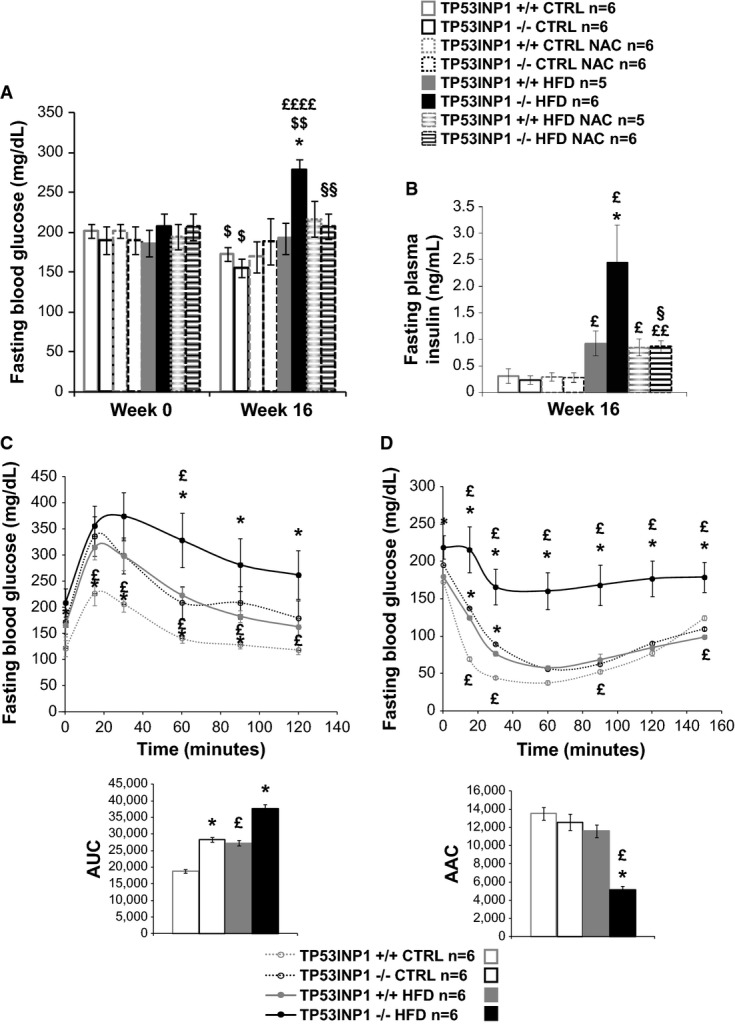
TP53INP1-deficient mice have moderate redox-related insulin resistance syndrome which is exacerbated by HFD protocol Male TP53INP1 KO and WT mice were fed a high-fat diet (HFD, 60% fat) or a control diet (CTRL) during 16 weeks. Mice drank tap water or NAC-supplemented tap water (1%).
A, B Histograms show blood glucose (A) or plasma insulin (B) levels of 6-h-fasted mice at the beginning (Week 0) and/or at the end of the protocol (Week 16). Fasting blood glucose week 16: *P* (−/− versus +/+; HFD) = 0.0052; *P* (CTRL versus HFD; −/−) = 0.000081; *P* (NAC versus no NAC; −/− HFD) = 0.0019; *P* (w16 versus w0; +/+ CTRL) = 0.012; *P* (w16 versus w0; −/− CTRL) = 0.050; *P* (w16 versus w0; −/− HFD) = 0.0023. Fasting plasma insulin: *P* (−/− versus +/+; HFD) = 0.043; *P* (CTRL versus HFD; +/+) = 0.028; *P* (CTRL versus HFD; −/−) = 0.013; *P* (CTRL versus HFD; +/+ NAC) = 0.011; *P* (CTRL versus HFD; −/− NAC) = 0.0015; *P* (NAC versus no NAC; −/− HFD) = 0.038.

As obesity is generally associated with insulin resistance (IR), we investigated the susceptibility of HFD-fed TP53INP1-deficient mice to develop IR, glucose intolerance and hyperinsulinemia. We monitored glycemia and insulinemia at the beginning and end of HFD protocol and determined the HOMA-IR index (Fig2A and B, respectively, and Supplementary Table S1). We also performed glucose tolerance (GTT) and insulin tolerance (ITT) tests at the end of HFD protocol (Fig2C and D, respectively). In HFD-fed WT animals, glucose utilization (GTT, Fig2C) and insulin sensitivity (ITT, Fig2D) were both altered as expected. This was compensated by hyperinsulinemia (Fig2B), while blood glucose remained unchanged (Fig2A), indicating that WT mice under HFD have developed IR. Interestingly, TP53INP1 knockout mice fed a standard diet were also glucose intolerant and insulin resistant, but neither hyperglycemic nor hyperinsulinemic. Glucose intolerance and IR further developed when TP53INP1-deficient mice were fed a HFD, and hyperinsulinemia finally occurred in such experimental conditions with plasma insulin levels twice as high in HFD-fed TP53INP1-deficient as in WT animals. As a consequence, the combined effects of HFD-induced obesity and the absence of TP53INP1 led to hyperglycemia (Fig2A and C), suggesting that these mice had developed T2D. In contrast, NAC-treated HFD-fed TP53INP1-deficient mice showed similar metabolic profiles to HFD-fed WT animals (Fig2A and B) indicative of chronic oxidative stress predisposing those mice to systemic IR, hyperinsulinemia, glucose intolerance and therefore T2D.

C Glucose tolerance test (GTT) was performed on 6-h-fasted mice during 120 min after injection of 1 g glucose/kg of body weight. Curves on the left show blood glucose level monitored after injection of glucose. Histograms on the right show area under curve (AUC). Fasting blood glucose: *P* (−/− versus +/+; CTRL; *t* = 0 min) = 0.045; *P* (−/− versus +/+; CTRL; *t* = 15 min) = 0.013; *P* (−/− versus +/+; CTRL; *t* = 30 min) = 0.016; *P* (−/− versus +/+; CTRL; *t* = 60 min) = 0.030; *P* (−/− versus +/+; CTRL; *t* = 90 min) = 0.017; *P* (−/− versus +/+; HFD; *t* = 60 min) = 0.041; *P* (−/− versus +/+; HFD; *t* = 90 min) = 0.043; *P* (−/− versus +/+; HFD; *t* = 120 min) = 0.034; *P* (HFD versus CTRL; +/+; *t* = 15 min) = 0.0076; *P* (HFD versus CTRL; +/+; *t* = 30 min) = 0.0067; *P* (HFD versus CTRL; +/+; *t* = 60 min) = 0.00058; *P* (HFD versus CTRL; +/+; *t* = 90 min) = 0.0010; *P* (HFD versus CTRL; +/+; *t* = 120 min) = 0.023; *P* (HFD versus CTRL; −/−; *t* = 60 min) = 0.032. AUC: *P* (−/− versus +/+; CTRL) = 0.023; *P* (−/− versus +/+; HFD) = 0.035; *P* (HFD versus CTRL; +/+) = 0.042.

D Insulin tolerance test (ITT) was performed on 6-h-fasted mice during 150 min after injection of 0.70 U insulin/kg of body weight. Curves on the left show blood glucose level monitored after injection of insulin. Histograms on the right show area above curve (AAC). Fasting blood glucose: *P* (−/− versus +/+; CTRL; *t* = 15 min = 0.012; *P* (−/− versus +/+; CTRL; *t* = 30 min) = 0.022; *P* (−/− versus +/+; HFD; *t* = 0 min) = 0.027; *P* (−/− versus +/+; HFD; *t* = 15 min) = 0.011; *P* (−/− versus +/+; HFD; *t* = 30 min) = 0.0037; *P* (−/− versus +/+; HFD; *t* = 60 min) = 0.0028; *P* (−/− versus +/+; HFD; *t* = 90 min) = 0.041; *P* (−/− versus +/+; HFD; *t* = 120 min) = 0.0032; *P* (−/− versus +/+; HFD; *t* = 150 min) = 0.0025; *P* (HFD versus CTRL; +/+; *t* = 15 min) = 0.0082; *P* (HFD versus CTRL; +/+; *t* = 30 min) = 0.033; *P* (HFD versus CTRL; +/+; *t* = 90 min) = 0.047; *P* (HFD versus CTRL; +/+; *t* = 150 min) = 0.028; *P* (HFD versus CTRL; −/−; *t* = 15 min) = 0.026; *P* (HFD versus CTRL; −/−; *t* = 30 min) = 0.0095; *P* (HFD versus CTRL; −/−; *t* = 60 min) = 0.0031; *P* (HFD versus CTRL; −/−; *t* = 90 min) = 0.033; *P* (HFD versus CTRL; −/−; *t* = 120 min) = 0.0068; *P* (HFD versus CTRL; −/−; *t* = 150 min) = 0.0082. AAC: *P* (−/− versus +/+; HFD) = 0.030; *P* (HFD versus CTRL; −/−) = 0.037.

Data information: Results are expressed as the mean ± SEM and are representative of two independent experiments. * TP53INP1 −/− versus TP53INP1 +/+; ^£^ HFD versus CTRL; ^$^ Week 16 versus Week 0; ^§^ NAC versus no NAC; 1 character: *P* < 0.05; 2 characters: *P* < 0.005; 4 characters: *P* < 0.00005.

**Figure 3:**
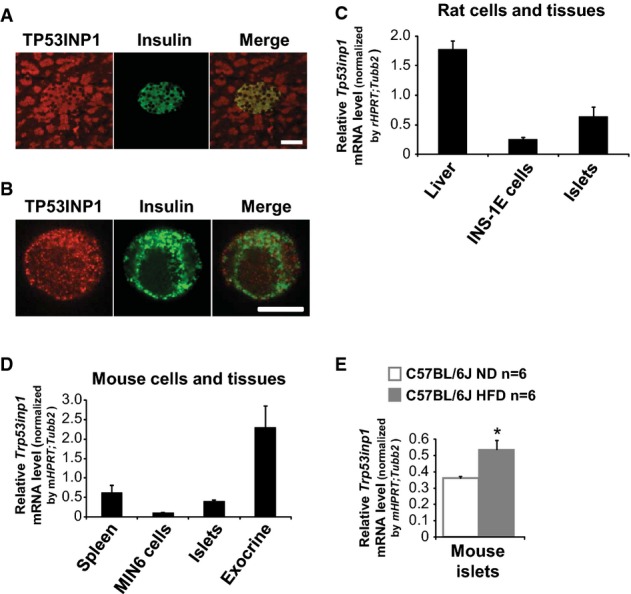
The gene encoding TP53INP1 is expressed in pancreatic endocrine cells

TP53INP1 mRNA has been reported to be present in human islets of Langerhans (∽30^th^ centile) (Eizirik *et al*, 2012). Using immunofluorescence to examine mouse pancreatic sections and human isolated β-cells (Fig3A and B), and quantitative PCR analysis of rodent cells and endocrine tissues (Fig3C and D), we found that *TP53INP1* was expressed both by pancreatic exocrine cells and by the insulin-secreting β-cells which play a central role in the control of glucose homeostasis. Because TP53INP1-deficient mice were glucose intolerant, and since *TP53INP1* transcripts were significantly increased in islets isolated from HFD-fed mice (Fig3E), we next hypothesized that defects in β-cell function or plasticity could occur in TP53INP1 knockout mice. However, neither functional modifications (glucose-induced insulin secretion, NADP(H) or cytosolic free calcium concentration, [Ca^2+^]_c_) nor changes in islet mass were detected in the absence of TP53INP1 (Supplementary Fig S4). These results suggest that HFD-fed TP53INP1 KO mice developed diabetes due to severe IR, which resulted from whole-body redox deregulation rather than specific endocrine pancreatic alterations. Nonetheless, the observed failure of β-cell mass or function to increase in response to elevated insulin demand suggests that TP53INP1 may also be required in β-cells to mount a compensatory response to IR.

A, B (A, B) Immunocytofluorescent staining of TP53INP1 (red) and insulin (green) in mouse pancreatic sections (A) and single human islet beta cell (B). Scale bars represent 50 μm (A) and 10 μm (B).

C–E Quantitative PCR for *Tp53inp1* mRNA levels in tissues and cells from rat (C) and C57BL/6J mice fed with a normal diet (5% fat; ND) or an high-fat diet (45% fat; HFD) (D, E). Results are expressed as the mean ± SEM and are representative of two independent experiments. *n* = 2 for rat liver, islets and mouse spleen; *n* = 4 for INS-1E cells; *n* = 5 for mouse exocrine pancreas; *n* = 6 for Min6 cells and ND and HFD islets; *n* = 11 for mouse islets. *P = 0.035 for HFD versus ND.

### Mitochondrial number is increased in the absence of TP53INP1, promoting chronic oxidative stress

**Figure 4:**
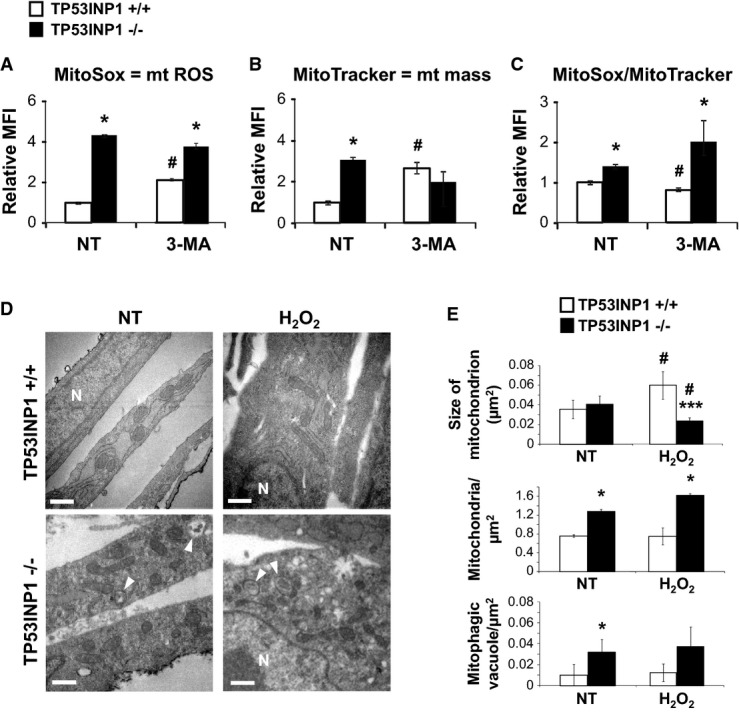
Absence of TP53INP1 increases mitochondrial (mt) ROS level and mass due to increased mitochondria number Immortalized MEFs (MEFi) deficient (−/−) or not (+/+) for TP53INP1 were left untreated (NT) or treated with 3-methyladenine (3-MA, 5 mM) during 4 h.

As susceptibility to obesity and T2D in TP53INP1-deficient mice is redox-linked, we addressed the question of the cellular origin of chronic oxidative stress in these mice (Gommeaux *et al*, 2007; Cano *et al*, 2009; N'Guessan *et al*, 2011). Oxidative stress could be due to ROS over-production in mitochondria which are the main source of ROS since superoxide is a by-product of respiratory chain (Murphy, 2009). We stained WT and TP53INP1-deficient cells (immortalized MEFs, depicted as MEFi in this manuscript) with a mitochondrial superoxide stain (MitoSox) and observed a fourfold higher level of staining in deficient cells compared to WT by flow cytometry analysis (Fig4A). Co-staining with the MitoTracker™ marker also showed that the mitochondrial mass was threefold higher in TP53INP1 KO cells than in WT (Fig4B). Normalization of MitoSox to MitoTracker staining (Fig4C) suggests that the increased mitochondrial mass is not the sole cause of ROS increase. Transmission electron microscopy (TEM; Fig4D and E) revealed a higher number of mitochondria in TP53INP1-deficient cells than in WT. This difference was not affected by H_2_O_2_ (oxidative stress) treatment, suggesting that increase in mitochondrial number is the cause and not the consequence of chronic oxidative stress in the absence of TP53INP1. Together, those data demonstrate an increase in both mitochondrial number and ROS-producing activity in the absence of TP53INP1.

Histograms show mt ROS level measured by flow cytometry upon MitoSox staining. *P* (−/− versus +/+; NT) = 0.019; *P* (−/− versus +/+; 3-MA) = 0.025; *P* (3-MA versus NT; +/+) = 0.031.

Histograms show mt mass evaluated by flow cytometry in KO or WT MEFi using MitoTracker staining. *P* (−/− versus +/+; NT) = 0.012; *P* (3-MA versus NT; +/+) = 0.036.

Histogram shows MitoSox fluorescence normalized with MitoTracker fluorescence. *P* (−/− versus +/+; NT) = 0.041; *P* (−/− versus +/+; 3-MA) = 0.030; *P* (3-MA versus NT; +/+) = 0.045.

After 4 h recovering in normal media, H_2_O_2_ (1 h, 100 μM) or non-treated (NT) MEFi deficient (−/−) or not (+/+) for TP53INP1 were observed by transmission electron microscopy (TEM). N = nucleus; white arrow = mitophagic vacuoles. Scale bar represents 0.5 μm.

Mean size of mitochondrion (area), number of mitochondria and mitophagic vacuoles normalized by cytoplasmic surface area were quantified. Size: *P* (−/− versus +/+; H_2_O_2_) = 0.000027; *P* (H_2_O_2_ versus NT; +/+) = 0.0070; *P* (H_2_O_2_ versus NT; −/−) = 0.013. Nb mito.: *P* (−/− versus +/+; NT) = 0.035; *P* (−/− versus +/+; H_2_O_2_) = 0.016. Nb vacuoles.: *P* (−/− versus +/+; NT) = 0.047.

Data information: Results are expressed as the mean ± SEM and are representative of three independent experiments. In (A-C): *P < 0.05 for TP53INP1−/− versus TP53INP1 +/+; ^#^*P* < 0.05 for 3-MA versus NT. In (E): * TP53INP1−/− versus TP53INP1 +/+; ^#^ H_2_O_2_ versus NT. 1 character: *P* < 0.05; 2 characters: *P* < 0.01; 3 characters: *P* < 0.0005.

### Increased mitochondrial number in the absence of TP53INP1 stems from impaired mitophagy of dysfunctional mitochondrial pool

We previously provided evidence for impaired autophagy in TP53INP1-deficient cells (N'Guessan *et al*, 2011; Seillier *et al*, 2012). Therefore, we addressed the possibility that dysregulation of the mitochondrial compartment might stem from the impaired elimination of damaged mitochondria by mitophagy. Treatment of MEFi with the autophagy inhibitor 3-MA (which blocks autophagy at early stage) led to both increased MitoSox and MitoTracker staining in WT cells (Fig4A–C). Remarkably, this increase was not observed in TP53INP1-deficient cells, suggesting that autophagic flow leading to mitophagy must be chronically impaired in these cells. To gain further insight into this issue, we quantified the number of mitophagic vacuoles in MEFi. Figure4D and E clearly show the presence of autophagic vacuoles containing mitochondria (entire or almost degraded) in TP53INP1 KO cells, whereas very few of these structures were seen in WT counterparts. As no change in mitochondrial mass was observed after autophagy inhibition in TP53INP1-deficient cells, this accumulation of mitophagic structures suggests a blockade of late stages in the mitophagic process, for example, fusion of autophagosomes with lysosomes, rather than increased mitophagic flow. These results were unchanged after H_2_O_2_ treatment, suggesting that oxidative stress observed in the absence of TP53INP1 was the consequence and not the cause of a defect in mitochondria degradation. Higher number of mitophagic structures in TP53INP1 KO cells than in WT was also indicated by LC3/mitotracker co-labeling and quantification by confocal fluorescence microscopy (Supplementary Fig S5).

**Figure 5:**
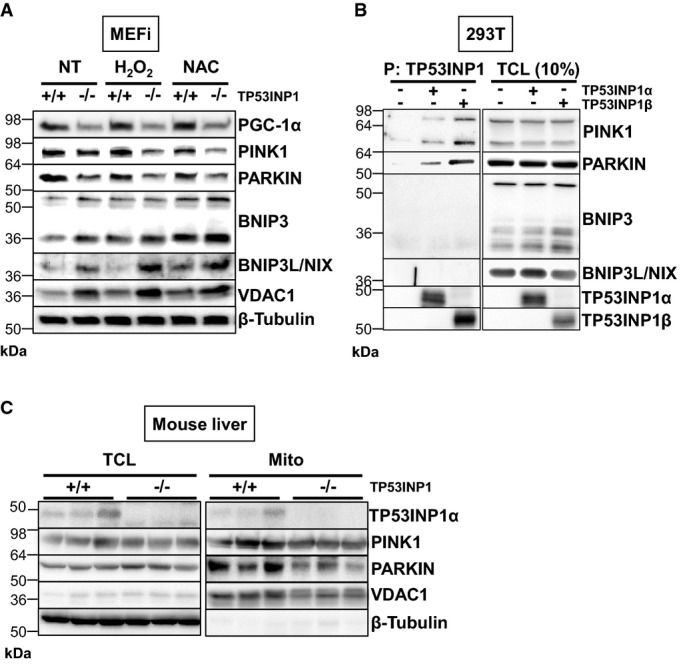
TP53INP1 deficiency is linked with impaired PINK/PARKIN mitophagy

Western blot analysis performed on MEFi lysates (Fig5A) showed higher levels of VDAC1, consistent with increased mitochondrial mass in TP53INP1 KO MEFi. By contrast, PINK1 and PARKIN levels were lowered in the absence of TP53INP1. As both PINK1 and PARKIN proteins are necessary for PINK/PARKIN-mediated mitophagy (following a loss of mitochondrial potential membrane in defective mitochondria) (Novak, 2012), this result is consistent with defective mitochondrial degradation by mitophagy. Neither oxidative stress nor antioxidant treatment (H_2_O_2_ and NAC treatment, respectively) impacted the lowered PINK1 and PARKIN levels in KO cells (Fig5A). Surprisingly, BNIP3 and NIX protein levels were increased in TP53INP1 KO cells compared to WT, suggesting that hypoxia-induced mitophagy, which implicates BNIP3 and NIX (Zhang & Ney, 2009), was not affected in TP53INP1-deficient cells. Lower PINK1 and PARKIN levels in the absence of TP53INP1 were also observed *in vivo* in mitochondria-enriched fractions from mouse liver (Fig5C right). Nevertheless, the clear decrease in PINK1/PARKIN level and increase in VDAC level in TP53INP1 −/− cells (Fig5A) were not totally recapitulated in the mice total liver lysates (Fig5C left).

After 4 h recovering in normal media, TCLs from H_2_O_2_- (1 h, 100 μM), NAC- (24 h, 10 mM) or non-treated (NT) MEFi deficient (−/−) or not (+/+) for TP53INP1 were analyzed by immunoblotting for PGC-1α, PINK1, PARKIN, BNIP3, BNIP3L/NIX, VDAC1 and β-tubulin.

HEK293T cells were cotransfected with plasmids encoding TP53INP1α-NTAP or TP53INP1β-NTAP. TP53INP1α- or β-NTAP was precipitated with a streptavidin-containing resin (P), resolved by PAGE and Western blots developed with anti-TP53INP1 (TP53INP1 precipitation control), anti-PINK1, anti-PARKIN, anti-BNIP3 or anti-BNIP3L/NIX antibody. Western blot on TCL (on the right) served as a transfection control.

Three-month-old TP53INP1-deficient and WT male mice were sacrificed and their livers harvested. Mitochondrial lysates (Mito) were purified from total liver lysates (TCL), and both were analyzed by immunoblotting for TP53INP1, PINK1, PARKIN, VDAC1 and β-tubulin.

Data information: Results are representative of three independent experiments. Source data are available online for this figure.

To gain insights into possible molecular partnerships between TP53INP1 and proteins involved in mitophagy, we performed immunoprecipitation assays. This provided further evidence for a direct interaction between each of the TP53INP1 isoforms (TP53INP1α or TP53INP1β) and both PINK1 and PARKIN, but not with BNIP3 or NIX (Fig5B). Interestingly, Fig5C shows detection of TP53INP1 in mitochondria-enriched fractions from WT liver, thus demonstrating a mitochondrial sub-cellular localization of TP53INP1, in addition to its known nucleo-cytoplasmic localization (Tomasini *et al*, 2001; Seillier *et al*, 2012). Moreover, restoration of TP53INP1 expression induced an increase in the level of both PINK1 and PARKIN in the mitochondrial fraction and confirmed localization of TP53INP1 to mitochondria (Supplementary Fig S6A). Finally, we observed a decreased level of PGC-1α (a regulator of mitochondrial biogenesis and function) in TP53INP1-deficient cells compared to WT (Fig5A), suggesting that mitochondrial accumulation in the absence of TP53INP1 does not rely on increased mitochondrial biogenesis. (We cannot exclude the possibility that the absence of TP53INP1 rather negatively impacts on mitochondrial biogenesis.) Taken together, these data suggest that (i) at least large in part, accumulation of mitochondria in TP53INP1-deficient cells results from a mitophagic defect and (ii) that TP53INP1 is able to participate in the completion of PINK/PARKIN-dependent mitophagy by direct physical interaction with these proteins.

**Figure 6:**
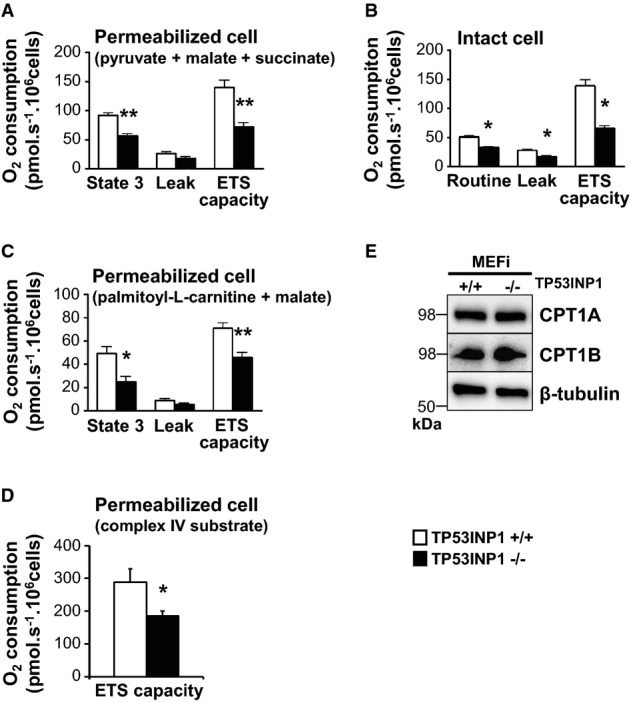
TP53INP1-deficient MEFi contain a dysfunctional mitochondrial pool

As part of mitochondrial ROS production was not related to an increase in mitochondrial mass (Fig4C), we assessed whether mitochondrial over-production of ROS may be due to dysfunctional mitochondria. Mitochondrial oxygen consumption analysis showed decreased oxygen flow in TP53INP1-deficient MEFi compared to WT, both in permeabilized and in intact cells, and whatever the substrate used (glucose versus lipid-related substrate) in the permeabilized cell setting (Fig6A–C). Thus, the oxidative phosphorylation system of TP53INP1 KO MEFi is impaired compared to WT, probably resulting from specific mitochondrial alterations as similar observations were made in intact and permeabilized cells. Decreased oxygen consumption in the setting of lipid substrates (Fig6C) is probably not related to decreased substrate availability since levels of CPT1A and CPT1B (two isoforms of CPT1, the key enzymes responsible for the mitochondrial uptake of long-chain fatty acids for beta-oxidation) did not differ between KO MEFi and WT cells (Fig6E). Analysis of respiratory chain complexes showed decreased activity of complex IV (Fig6D), without any change in complex expression as quantified by Western blotting (L. Peyta, S. Servais, unpublished data). Finally, analysis of mitochondrial shape and size by transmission electron microscopy (TEM; Fig4D and E) showed that upon H_2_O_2_ treatment, mitochondria became bigger (longer) in WT cells probably as a result of stress-induced mitochondrial fusion. By contrast, mitochondria occupied a smaller surface in H_2_O_2_-treated TP53INP1 KO cells, suggesting an impaired ability of TP53INP1-deficient mitochondria to cope with stress.

A–D High-resolution respirometry was performed on permeabilized MEFi TP53INP1 deficient (−/−, black bars) or not (+/+, white bars) using glucose (A), lipid-related substrates (C) or complex IV substrate (D). High-resolution respirometry was also performed on intact MEFi (B). For different respiratory state details (routine, 3, leak, and ETS capacity), please refer to the Materials and Methods section. Results are expressed as the mean ± SEM and are representative of three independent experiments. In (A): *P* (−/− versus +/+; State 3) = 0.0079; *P* (−/− versus +/+; ETS) = 0.0079; *n* = 5 in each group. In (B): *P* (−/− versus +/+; routine) = 0.0159; *P* (−/− versus +/+; leak) = 0.0159; *P* (−/− versus +/+; ETS) = 0.0159; *n* = 5 in each group. In (C): *P* (−/− versus +/+; State 3) = 0.029; *P* (−/− versus +/+; ETS) = 0.0079; *n* = 5 in each group. In (D): *P* (−/− versus +/+; ETS) = 0.0259; *n* = 5 in each group. *TP53INP1−/− versus TP53INP1 +/+; 1 character: *P* < 0.05; 2 characters: *P* < 0.01.

E TCLs from MEFi were analyzed by immunoblotting for CPT1A, CPT1B and β-tubulin. Results are representative of three independent experiments.

Source data are available online for this figure.

Collectively, our data show accumulation of defective mitochondria within TP53INP1-deficient MEFi associated with impaired mitophagy. The cumulative effect of increased mitochondrial number and their dysfunctional state is likely at the origin of ROS over-production and chronic oxidative stress observed in the absence of TP53INP1.

### Accumulation of lipid droplets is linked with oxidative stress in TP53INP1-deficient cells

**Figure 7:**
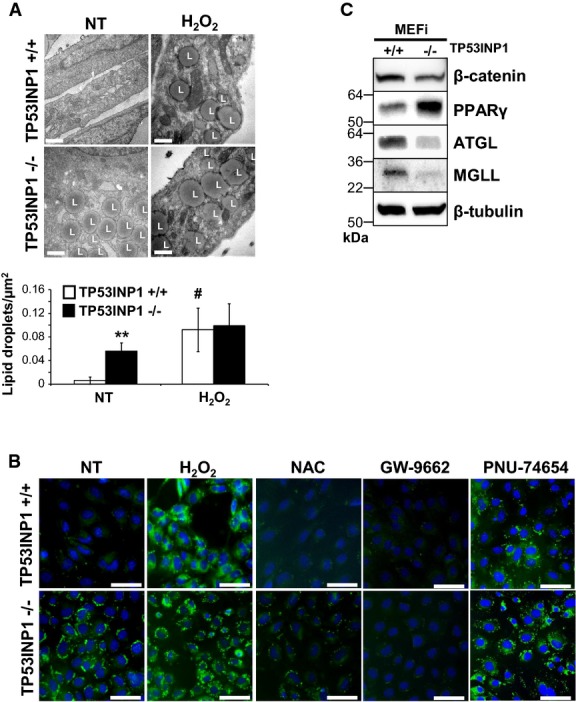
Oxidative stress observed in absence of TP53INP1 is at the origin of presence of lipid droplets (LD) in MEFi MEFi deficient (−/−) or not (+/+) for TP53INP1 were seeded in media containing or not 10 mM NAC. Forty-eight hours later, MEFi were treated or not (NT) with 100 μM hydrogen peroxide (H_2_O_2_) during 1 h in serum free media. Cells were left to recover for 4 h in normal media containing or not NAC (10 mM), GW-9662 (PPARγ inhibitor, 10 μM) or PNU-74654 (Wnt/β-catenin pathway inhibitor, 50 μM) before being harvested. Cells were observed by TEM.
Number of LD normalized by cytoplasmic surface area is shown in both histograms at the bottom of the figure. Scale bar represents 0.5 μm. Results are expressed as the mean ± SEM and are representative of three independent experiments. *P (−/− versus +/+; NT) = 0.00086; ^#^*P* (H_2_O_2_ versus NT; +/+) = 0.013.

TEM analysis of MEFs revealed a huge number of lipid droplets (LD) in TP53INP1-deficient MEFi, whereas these were absent from control MEFs (Fig7A). Staining of these LD using the fluorescent Bodipy compound (Fig7B, left panel) confirmed that TP53INP1 KO MEFs contain far more LD than WT cells. To assess whether this feature could be linked to increased ROS production in deficient cells, we analyzed LD in H_2_O_2_-treated cells (Fig7A and B). Upon oxidative stress, WT cells showed a striking increase in LD number which was not observed in TP53INP1 KO cells. Reciprocally, we treated cells with the antioxidant NAC (Fig7B) and observed a reduction in LD number in both genotypes. Results obtained in primary MEFs corroborate those in MEFi (Supplementary Fig S7), showing that what we observed in MEFi is directly linked to the absence of TP53INP1 and not immortalization event(s). This study thus shows that oxidant treatment increases LD number in WT cells but, conversely, antioxidant treatment reduces LD number in TP53INP1 KO cells, both treatments abolishing differences existing between WT and KO cells. To gain molecular insight into these metabolic alterations, and based on a transcriptomic analysis of MEFi KO cells compared to WT (M. Seillier, A. Carrier, unpublished data), we showed by Western blotting an over-expression of PPARγ in KO cells, while expression of β-catenin was decreased (Fig7C). It is known that the Wnt/β-catenin pathway (canonical Wnt pathway) inhibits expression of the gene coding PPARγ, a positive regulator of adipo- and lipogenesis. Consistently, treatment with a PPARγ inhibitor (GW-9662) prevented LD accumulation, whereas treatment with a Wnt/β-catenin pathway inhibitor (PNU-74654) induced LD formation in both genotypes (Fig7B), suggesting that regulation of PPARγ expression by canonical Wnt pathway is directly involved in LD accumulation in TP53INP1 KO cells. Finally, restoration of TP53INP1 expression led to decreased PPARγ expression (Supplementary Fig S6B) as well as decreased accumulation of LD (Supplementary Fig S6C). Western blotting analysis also showed decreased expression of lipases (ATGL and MGLL) in the absence of TP53INP1 (Fig7C), in agreement with increased LD content in MEFi KO cells. Taken together, those data provide molecular insights into redox-linked lipid metabolism defects in the absence of TP53INP1. We can conclude from these experiments that chronic oxidative stress observed in TP53INP1-deficient cells is at the origin of the accumulation of LD, which are likely involved in the *in vivo* increased fat depot and hepatic steatosis associated with HFD-induced obesity.

Cells were also observed by fluorescence microscopy after Bodipy493/503 staining of LD (green) and DAPI staining of nucleus (blue). L = lipid droplets. Scale bar represents 100 μm. Results are representative of three independent experiments.

TCLs from MEFi were analyzed by immunoblotting for β-catenin, PPARγ, ATGL, MGLL and β-tubulin. Results are representative of three independent experiments.

Source data are available online for this figure.

## Discussion

This work addresses the function of *TP53INP1* as a T2D susceptibility gene as proposed 5 years ago (Voight *et al*, 2010; Cauchi *et al*, 2012) and provides both the demonstration and a mechanism for this function. We should note, however, that at present there is no evidence that the *TP53INP1* locus is associated with obesity in man. Nonetheless, in our mouse model, the absence of TP53INP1 favors increased body weight and fat mass, most spectacularly in an experimental setting of obesity induced by lipid-rich diet. Use of an antioxidant treatment, which alleviates the chronic oxidative stress affecting TP53INP1-deficient animals (N'Guessan *et al*, 2011), completely abolishes the predisposition to obesity, demonstrating that oxidative stress is the cause of exacerbated obesity in these animals. The present data thus provide the novel finding that oxidative stress may also be the cause of obesity and not only the well-known consequence of a chronic low-grade inflammatory state associated with obesity. By its action on redox status homeostasis, TP53INP1 is thus a new molecular actor of obesity prevention, and the current work thus adds to the knowledge of the molecular events involved in obesity predisposition.

We also observed that a lipid-rich diet reinforces the predisposition of TP53INP1-deficient mice to IR. Indeed, basal glycemia and insulinemia are high in obese (HFD-fed) TP53INP1-deficient mice, this feature being completely prevented by antioxidant treatment showing a key role of oxidative stress. Additionally, glycemia of obese TP53INP1-deficient mice is barely affected after insulin injection in the ITT experimental setting. Interestingly, glycemia of CTRL-fed TP53INP1-deficient mice follows the same curve as HFD-fed WT mice, suggesting that absence of TP53INP1 by itself favors IR usually related to obesity. Thus, emergence of IR in TP53INP1 mutant mice appears to result from chronic oxidative stress, without a requirement for increased adiposity. Adiposity may nonetheless strengthen the effects of the existing oxidative stress and IR, a novel observation of the current work. Importantly, our studies provide evidence, through the generation of animal model, of the molecular mechanisms through which the *TP53INP1* gene influences T2D predisposition in man (Voight *et al*, 2010). Intriguingly, our data suggest that antioxidants may be of use in individuals with T2D variants at the *TP53INP1* locus that affect diabetes risk.

We note that the variant at the *TP53INP1* locus on chromosome 8 (rs896854, lying in intron 1) associated with T2D in GWAS studies (Voight *et al*, 2010) appears to lead to impaired β-cell function (i.e. reduced insulin secretion), as measured by a decrease in HOMA-B (Voight *et al*, 2010) and unchanged insulin sensitivity. Moreover, dysglycemia necessarily involves a failure in pancreatic insulin secretion. The present data may suggest that, as well as being more insulin resistant, TP53INP1-deficient mice also possess β-cells which may be more susceptible to the effects of global lipid dysregulation. Interestingly, there are also decreases in miRNAs that regulate *TP53INP1* in human diabetic islets (Kameswaran *et al*, 2014), suggesting that over-expression of *TP53INP1* may occur in β-cells in diabetes. At present, it is unclear whether the risk variant at rs896854 affects the expression of *TP53INP1* (or that of other genes in this locus), in which tissues and in what direction. Future eQTL studies will be needed to answer this question.

Remarkably, our study provides a mechanism for TP53INP1 function in T2D. Increased predisposition of TP53INP1-deficient mice to adiposity is mirrored *in vitro* by a massive LD accumulation within TP53INP1-deficient cells, also dependent on chronic oxidative stress. At the molecular level, this LD accumulation in TP53INP1 KO cells is directly correlated with decrease in lipases expression, but above all with decrease in β-catenin expression consistent with an increase of PPARγ expression. PPARγ is a major player in lipogenesis and LD formation whose expression is inhibited by the Wnt/β-catenin pathway (Moldes *et al*, 2003; Sahini & Borlak, 2014). Restoration of TP53INP1 expression decreases PPARγ expression as well as accumulation of LD in close link with dampening of oxidative stress (Cano *et al*, 2009). Taken together, these data provide molecular insights into redox-linked lipid metabolism defects in the absence of TP53INP1.

Regarding the origin of chronic oxidative stress associated with TP53INP1 deficiency, we provide data in favor of deregulated mitochondrial homeostasis in the absence of TP53INP1. We reveal that TP53INP1-deficient cells produce high levels of mitochondria-derived ROS, resulting from both respiratory chain defect and high number of mitochondria. Furthermore, we show that this latter is the consequence, at least in part, of impaired PINK/PARKIN mitophagy (Novak, 2012) in TP53INP1-deficient cells, consistent with our previous work reporting a role for TP53INP1 in autophagy (N'Guessan *et al*, 2011; Seillier *et al*, 2012). PINK/PARKIN mitophagy involves mitochondrial protein ubiquitination and p62 protein cargo. Interestingly, our laboratory previously provided evidence that TP53INP1 is able to compete with p62 for interaction with LC3 thanks to its better LIR affinity (Seillier *et al*, 2012). Thus, TP53INP1 could be directly involved in mitophagy by playing the role of a protein cargo directing mitochondria for degradation to autophagosomes. Alternatively, accumulation of mitophagic vacuoles in the absence of TP53INP1 could mean that TP53INP1 participates in the completion of PINK/PARKIN-dependent mitophagy. Data shown in the present work, that is, (i) a direct physical interaction of TP53INP1 with PINK1 and PARKIN, (ii) the influence of TP53INP1 expression on PINK1 and PARKIN levels in mitochondria (demonstrated both in loss- and gain-of-function cell models and in KO livers) and (iii) the subcellular localization of TP53INP1 in mitochondria, are in favor of the contribution of TP53INP1 in PINK/PARKIN-dependent mitophagy.

Finally, it is interesting to note that the TP53INP1 paralog TP53INP2 (also known as DOR for diabetes and obesity related) is also involved in MS through its implication in autophagy (Nowak *et al*, 2009; Mauvezin *et al*, 2010; Sala *et al*, 2014).

To conclude, this work provides a preclinical model of mice prone to MS. IR establishment in TP53INP1-deficient mice is favored by chronic oxidative stress, which drives LD accumulation. Furthermore, this work highlights the underlying mechanism by showing that chronic oxidative stress observed in the absence of TP53INP1 stems from impaired mitophagy of a dysfunctional mitochondrial pool (graphical abstract shown in Supplementary Fig S8). Therefore, TP53INP1 mutant mice constitute an original model in which to study the implication of oxidative stress and autophagy in the development of obesity and T2D, two crucial public health issues.

## Materials and Methods

### Mice

Generation of TP53INP1-deficient (*Trp53inp1*^−/−^) mice backcrossed on the C57BL/6 parental genetic background and their genotyping by PCR were described previously (Gommeaux *et al*, 2007; N'Guessan *et al*, 2011). TP53INP1 WT and KO mice originate from heterozygous (*Trp53inp1*^*+/−*^) pairs. Mice entered protocols at 8 weeks of age and were analyzed between 22 and 26 weeks of age according to protocols. When not in HFD or GTT/ITT protocols, mice were weighed at 8 weeks and sacrificed at 22 weeks (5 months) of age. At sacrifice, organs (liver, gonadal and renal fat masses) were removed and weighed and then flash-frozen or fixed in 4% formaldehyde for further analyzes. All mice were kept within the animal facilities and according to the policies of the Laboratoire d'Exploration Fonctionnelle de Luminy (Marseilles, France). WT mice used in Fig3A, D and E and Supplementary Fig S2C were C57BL/6J (i.e. C57BL/6 from Jackson Laboratory).

### HFD protocol

Male mice (8 week old) were used since (i) weight gain upon aging of TP53INP1 KO compared to WT males is more pronounced than females, and (ii) males gain more weight than females when fed with HFD (Hwang *et al*, 2010). Mice were randomly assigned to three cohorts (HFD protocol, HFD protocol with NAC, HFD protocol followed by GTT and ITT) containing four groups each (TP53INP1 WT CTRL and HFD and TP53INP1 KO CTRL and HFD). Mice were housed (two mice per cage) under a 12-h light and 12-h dark cycle. They were fed with either standard chow (CTRL: Special Diet Services #824050; 20% protein, 10% fat, 70% carbohydrates) or high-fat diet (HFD: Special Diet Services #824054; 20% protein, 60% fat, 20% carbohydrates) with *ad libitum* access to food. During 16 weeks (or 18 weeks for GTT/ITT cohort), weekly food intake was measured by monitoring the weight of the remaining food at cage changing, always at the same daytime moment. Concurrently, mice were weighed. One cohort was given antioxidant NAC (Sigma-Aldrich, Saint-Quentin Fallavier, France) at 10 mg/ml (1%) in drinking water during all protocol. Except for GTT/ITT cohort, glycemia was taken at beginning and end of the protocol using a hand-held glucometer (OneTouch® Ultra®; Lifescan, Issy les Moulineaux, France) from blood sampled from the tail vein of 6-h-fasted mice. At week 16, mice were sacrificed by cervical dislocation, and organs were weighed and stored.

### Glucose tolerance and insulin tolerance tests (GTT and ITT)

For the GTT/ITT cohort, GTT test was performed at week 16 of HFD protocol, ITT at week 17, and sacrifice at week 18. Glucose (1 g/kg body weight, GTT) or insulin (0.70 U/kg body weight, NovoRapid®, Novo Nordisk®, Chartres, France, ITT) was injected intraperitoneally to 6-h-fasted mice. Glycemia was taken from tail vein blood sample before and after injection at different time points during 120 (GTT) or 150 (ITT) min. Area under curve (AUC; GTT) and area above curve (AAC; ITT) were determined using Excel and the trapezoidal rule.

### Plasma insulin level measurement

Plasma insulin levels were determined using a sensitive rat insulin RIA kits according to manufacturer instructions (Millipore, Molsheim, France).

### NADP(H), [Ca^2+^]_c_ and insulin secretion

Islets were isolated after collagenase digestion of the pancreas from 3-month-old TP53INP1 WT and KO male mice and were used after an overnight culture in RPMI-1640 (Life Technologies, Cergy Pontoise, France) supplemented with 10 mM glucose and 10% fetal bovine serum (FBS). The medium used for the experiments was a Bicarbonate KRB supplemented with 3 mM glucose. For [Ca^2+^]_c_, NADP(H) and insulin secretion, the experiments were performed on 17 (WT) and 21 (KO) islets as previously reported (Ravier *et al*, 2014).

### Histological analysis

#### Liver

Formaldehyde-fixed paraffin-embedded liver sections from HFD protocol mice were deparaffinized and stained with hematoxylin/eosin. Integrality of mounted stained sections surfaces was viewed on a Nikon microscope, and representative pictures were shown.

#### Pancreas

Pancreases from 3-month-old TP53INP1 WT (*n* = 4) and KO (*n* = 4) male mice were spread into flat embedding cassettes, fixed with 4% paraformaldehyde, paraffin embedded and longitudinally sectioned through the pancreatic head-to-tail axis (4 μm thickness). Three pancreatic sections per mouse, separated by at least 100 μm, were stained with hematoxylin/eosin and scanned using a NanoZoomer slide scanner (Hamamatsu Photonics, Hamamatsu City, Japan). The islet numbers were quantified, and the area of each islet was measured with the NDP.view software, version 1.2.

### Cell culture

Cell lines were cultured in DMEM Glutamax medium (Life Technologies) supplemented with 10% (FBS) in a humidified atmosphere with 5% CO_2_ at 37°C. TP53INP1 WT and TP53INP1 KO primary MEFs were obtained from embryos derived from homozygous breeding at 14.5 days postcoitum (E14.5) according to standard procedure. Immortalized MEFs (MEFi) were prepared from primary MEFs by sequential passages as for 3T3 cell line establishment. MEFs were cultured in the presence of penicillin (100 mU/ml) and streptomycin (100 mg/ml). They were used at early passages. HEK293T and U2OS cell lines were purchased from the American Type Culture Collection (Manassas, VA, USA). TP53INP1α-inducible U2OS cells (U2OSi), obtained by stable cotransfection with pVgRXR and pIND-TP53INP1α-EGFP vectors as previously reported (Gironella *et al*, 2007), were cultured in the presence of zeocin (0.05 mg/ml) and G418 (0.2 mg/ml).

### Cell treatments

Cells were plated in medium supplemented or not with 10 mM NAC and either treated 48 h later (80% confluency) with 1 mM hydrogen peroxide (H_2_O_2_) (Sigma-Aldrich) directly added in culture media or left untreated. After 1 h, cells were thoroughly rinsed twice with PBS and incubated 4 h with new culture medium containing or not 5 mM 3-MA (Early autophagic blocker, Sigma-Aldrich), 10 mM NAC, 10 μM GW9662 (Irreversible PPARγ antagonist; Sigma-Aldrich) and 50 μM PNU-74654 (Wnt/β-catenin pathway inhibitor; Sigma-Aldrich).

### Induction and transfection

In TP53INP1α-inducible U2OS cells, TP53INP1α-GFP expression was induced on 70% confluent cells using 10 μM ponasterone A (Life Technologies) for 24 h. DNA transfections were performed using FuGENE HD (Promega) according to the manufacturer's instructions on 75% confluent cells 24 h before experiment.

### Flow cytometry

MEFi were incubated during 10 min at 37°C with 5 μM MitoSOX™ Red (Life Technologies #M36008) or 200 nM MitoTracker® Deep Red (Life Technologies #M22426) diluted in HBSS (Hank's buffered salt solution; Life Technologies). Cells were trypsinized and rinsed by centrifugation (1,200 *g*, 5 min) with PBS. They were then analyzed on a FACSCalibur flow cytometer (BD Biosciences, Le Pont de Claix, France). Data analysis was performed using FlowJO (Treestar, Olten, Switzerland) software.

### Transmission electron microscopy (TEM)

MEFi were seeded in 10-cm dishes (7.5 × 10^6^ cells) 48 h before treatment. Cells were then rinsed twice with PBS and fixed for 1 h in cacodylate sodium buffer 0.2 M, pH 7.2, containing 2.5% glutaraldehyde, 8% paraformaldehyde and 0.01% CaCl_2_. Samples were then treated as described previously (Baron Gaillard *et al*, 2011). Observations were performed on an EM 912 electron microscope (Zeiss) at 100-kV acceleration equipped with a BioScan camera (Model 792; Gatan, Warrendale, PA, USA). Images were acquired with the Digital Micrograph software (Gatan). Quantification of the number of mitochondria, mitophagic vacuoles and lipid droplets normalized by cytoplasmic surface area was done by counting these structures on 40 images for which surface occupied by cell cytoplasm has been determined. Quantification of mean size of mitochondria was done by using area of an ellipse formula (area = longer diameter × smaller diameter × π), longer diameter of mitochondria being determined on images and smaller diameter considered as half of longer diameter.

### High-resolution respirometry

High-resolution respirometry was performed using a 2-ml chamber OROBOROS® Oxygraph 2K (Oroboros Instruments, Innsbruck, Austria) at 37°C. Respiration rates were calculated as the time derivative of oxygen concentration measured in the closed respirometer and expressed per million viable cells and corrected by non-mitochondrial oxygen consumption (antimycin A). The amplified signal was recorded in a computer with online display of the calibrated oxygen concentration and oxygen flux (DatLab software for data acquisition and analysis; Oroboros Instruments). Intact cells (0.5 × 10^6^ cells/ml) were analyzed in their respective bioenergetic substrate-containing cultivation medium. ROUTINE respiration (no additions), LEAK respiration (oligomycin-inhibited, 8 μg/ml) and ETS capacity (maximum non-coupled respiration induced by stepwise (typically 2–3 steps) titration of carbonyl cyanide *p*-(trifluoromethoxy) phenylhydrazone (FCCP), 0.8 mM dissolved in ethanol) were determined. Oxygen consumption was also measured on cells permeabilized with digitonin (8 μg/ml) in respiration buffer (10 mM KH_2_PO_4_, 300 mM mannitol, 10 mM KCl, 5 mM MgCl_2_, 1 mM EGTA and 1 mg/ml BSA fatty acid free) at final cell densities 0.5 × 10^6^ cells per milliliter. Respiration state 3 and 4 (LEAK) and ETS capacity were measured with pyruvate, malate and succinate (5, 5 and 10 mM, respectively) or palmitoylcarnitine and malate (40 μM and 5 mM, respectively). Activity of complex IV (cytochrome c oxidase) was determined by oxygraphy with trimethyl pentanediol (TMPD) as substrate.

### Fluorescence microscopy

#### Bodipy staining

Since Bodipy fluoresces in green, we used expression vectors encoding TP53INP1 isoforms tagged in red fluorescence. U2OSi cells were cotransfected by TP53INP1α + β-DsRed-N1 vector (containing human full-length TP53INP1 cDNAs) or empty pcDNA4/V5-His vector (Clontech, Saint-Germain-en-Laye, France) 24 h prior experiment. Cells (MEFi or U2OS) plated on glass coverslips in 12-well plates were treated as indicated and then fixed with 4% paraformaldehyde. Cells were washed with PBS and stained with BODIPY® 493–503 7.5 μg/ml (Life Technologies #D3922) in PBS during 5 min. Slides were then mounted in ProLong Gold antifade reagent with DAPI (Invitrogen) for imaging. Fluorescent images were captured using a Nikon microscope Eclipse 90I.

#### Mitotracker-LC3 co-staining

MEFi cells were plated on glass coverslips in 12-well plates 48 h prior experiment. Cells were incubated in a humidified atmosphere with 5% CO_2_ at 37°C during 15 min with MitoTracker® Red CMXRos 100 nM (Life Technologies #M-7512) diluted in warmed culture medium. After 2 washes with 1× PBS, cells were fixed during 15 min at 37°C with warmed 4% paraformaldehyde. Following fixation, cells were incubated in blocking buffer (3% BSA, 0.01% saponin in 1× PBS) containing 0.2 M glycine and 10% goat serum for 30 min. Then, cells were incubated 1 h at room temperature with primary antibody anti-LC3 (PM036-1:500; MBL, Nanterre, France) diluted in blocking buffer and washed three times before the addition of secondary antibody (Alexa Fluor 488 goat anti-rabbit, A-11034-1:500; Invitrogen). After 1 h of incubation at room temperature, cells were washed three times and slides were mounted in ProLong Gold antifade reagent with DAPI (Invitrogen) for imaging. Fluorescent images were captured using a Zeiss LSM 510 confocal microscope (Carl Zeiss, Le Pecq, France). Quantification of Mitotracker-LC3-positive puncta (= mitophagic figures) was done in 10 cells per genotype in three independent experiments.

#### Insulin/TP53INP1 co-staining

Insulin and TP53INP1 immunoreactivity were revealed with an anti-insulin antibody (1:200, DakoCytomation, Ely, UK) and with E12 mAb specific of TP53INP1 (1:100) (Gironella *et al*, 2007), respectively. Images were captured from mouse pancreatic sections and single human islet beta cell using a Zeiss Nipkov spinning disk confocal microscope (Hodson *et al*, 2013). Base images were captured and exported as TIFF files; figures were created using Adobe Photoshop 11. No manipulations other than global contrast and brightness adjustments were performed on images.

### Denaturing lysis

Cells were resuspended in denaturing lysis buffer (8 M urea, 0.1 M Na_2_HPO_4_/NaH_2_PO_4_, 0.01 M Tris–HCl, pH 8.0, 0.5% Triton X-100). Then, proteins were resolved by SDS–PAGE and immunoblotted.

### Mitochondria isolation

#### From cells

TP53INP1α-inducible U2OS cells were seeded in 15-cm dishes (7.5 × 10^6^ cells) 48 h before being collected by scrapping. Mitochondria were isolated according to the manufacturer's instructions of Mitochondria Isolation Kit for Cultured Cells (Abcam #ab110171, Paris, France). Briefly, after one freeze–thaw cycle, pellets from 2 dishes were resuspended in 750 μl Reagent A at 5.0 mg/ml protein and incubated on ice for 10 min. Cells were then disrupted with a Dounce homogenizer. After centrifugation (1,000 *g*, 10 min, 4°C), supernatants (SN1) were saved. Pellets were resuspended in 750 μl Reagent B, ruptured with Dounce homogenizer and centrifugated again. Supernatants (SN2) were collected and added to SN1. Few microliters of SN1 + SN2 was kept as total protein cell lysates. After spinning of remaining SN1 + SN2 (12,000 *g*, 10 min, 4°C), supernatants were discarded: pellets were resuspended in 450 μl Reagent C containing protease inhibitors cocktail (Sigma-Aldrich), forming mitochondrial lysates.

#### From tissue

Six TP53INP1 WT and six KO male mice (3 month old) were sacrificed and their livers sampled. Mitochondrial lysates were extracted from 300 mg of fresh liver according to the manufacturer's instructions of Mitochondria Isolation Kit for Tissue (Abcam #ab110169). A small volume of lysates before first high-speed centrifugation (12,000 *g*) was kept as total protein lysates.

### Coprecipitation assay

HEK293T cells in six-well plates were cotransfected by TP53INP1α- or β-NTAP vector (containing human full-length TP53INP1 cDNAs) or empty pNTAP vector (Agilent, Massy, France). After 24 h of transfection, cells were harvested by scraping and lysed and protein complexes were purified using streptavidin-containing resin according to the manufacturer's instructions of InterPlay Mammalian TAP Purification Kit (Agilent). Then, proteins were resolved by SDS–PAGE and immunoblotted.

### Immunoblotting

After boiling in Laemmli buffer (20 mM Tris pH 6.8, 2 M β-mercaptoethanol, SDS 9%, glycerol 30%), proteins from total lysates, mitochondrial lysates or co-immunoprecipitations were resolved by SDS–PAGE, transferred to polyvinylidene fluoride membrane, blocked in 5% non-fat milk in phosphate-buffered saline (PBS)/Tween-20, blotted and developed with antibodies specific for ATGL (2439-1:1,000; Cell Signaling Technology, Saint-Quentin-en-Yvelines, France), BNIP3 (ab10433-1:250; Abcam), BNIP3L/NIX (ab8399-1:1,000; Abcam), CPT1A (ab128568-1:800; Abcam), CPT1B (ab104662-1:800; Abcam), MGLL (ab119777-1:1,000; Abcam), PARKIN (ab15954-1:1,050; Abcam), PGC-1α (sc13067-1:200; Santa Cruz Biotechnology, Heidelberg, Germany), PINK1 (BC100-494-1:500; Novus Biologicals, Montluçon, France), PPARγ (sc72-73-1:1,250; Santa Cruz Biotechnology), TP53INP1 [rat monoclonal antibody generated in our laboratory, clone F8 (Saadi *et al*, 2013), 2 μg/ml], VDAC1 (ab14734-1:2,500; Abcam), β-catenin (sc1496-1:500; Santa Cruz Biotechnology) or β-tubulin (T4026-1:4,000; Sigma-Aldrich). Secondary antibodies were purchased from Santa Cruz Biotechnology: anti-rabbit horseradish peroxidase (HRP) conjugate (sc-2004-1:4,000) and anti-mouse HRP conjugate (sc-2005-1:6,000). Immunoblots were developed using the Immobilon Western Chemiluminescent HRP Substrate (Millipore). Chemiluminescence was detected using Fusion FX7 device (Fisher Bioblock Scientific, Illkirch, France). The experiments for the immunoblotting were performed at least three times with comparable results.

### Real-time quantitative PCR

Total RNAs from islets and exocrine pancreas (from mouse and rat) were extracted and DNase-treated using the RNeasy microkit from Qiagen, according to the manufacturer's instructions. Total RNAs from other tissues (spleen, liver) and β-cell lines were extracted using RNAnow (Ozyme) and treated with DNase (Ambion) according to the manufacturers' instructions. For HFD islets, C57BL/6J female mice purchased from Charles River (France) were fed either with a standard normal diet (ND, 5 kcal % fat) or a HFD containing lard (45 kcal % fat; D12451; Research Diets, New Brunswick, NJ, USA) from 8 weeks for 23 weeks. Islets were isolated after collagenase digestion of the pancreas (Broca *et al*, 2009). The quality of the islet preparations was validated by measuring the expression of *Insulin* and *Elastase 3b* by RT–qPCR in both exocrine and islet RNAs. The presence of exocrine tissue in the islets was < 5% (mean 1.7%, not shown). INS-1E and MIN6 cells were grown and maintained as described previously (Broca *et al*, 2009; Quoyer *et al*, 2010).

Reverse transcription was performed on total RNA using random hexamer oligonucleotides and MoMuLV-RT (Invitrogen). Real-time PCR amplification was performed in duplicate, using the 7500 System (Applied Biosystems) and according to the manufacturer's instructions. The sequences of the primers used were as follows: *Trp53inp1*: GTTGACTTCATAGATACCTGCCC/GTGTGCTCTGCTGAGGACTC which were validated both on rat and mouse cDNAs; *Tubb2*: CAAGGCTTTCCTGCACTGGT/AACTCCATCTCGTCCATGCC, which were validated both on rat and mouse cDNAs; *mHprt*: GCAGTACAGCCCCAAAATGG/GGTCCTTTTCACCAGCAAGCT; and *rHprt*: CAAAATGGTTAAGGTTGCAAGCT/AACACTTCGAGAGGTCCTTTTCAC. The level of expression of each gene X was normalized to the geometric mean of the expression levels of two housekeeping genes (*Hprt* and *Tubb2*) according to the formula: X/geometric mean (*Hprt*, *Tubb2*) = 2^−(*C*t(X) − arithmetic mean (*C*t(Hprt),*C*t(Tubb2)))^, where *C*t is the threshold cycle.

### Statistical analysis

Results are expressed as the mean ± SEM of results from at least two independent experiments. Statistical analyses were mainly performed via Student's *t*-tests except Mann–Whitney *U*-tests for Fig6. *P* < 0.05 was considered as significant.

### Human studies

Morbidly obese patients (*n* = 39) were recruited through the Department of Digestive Surgery and Liver Transplantation (Nice Hospital) where they underwent bariatric surgery for their morbid obesity. Bariatric surgery was indicated for these patients in accordance with French guidelines. Exclusion criteria were presence of a hepatitis B or hepatitis C infection, excessive alcohol consumption (> 20 g/day) or another cause of chronic liver disease as previously described (Anty *et al*, 2006; Bekri *et al*, 2006; Bertola *et al*, 2009). The characteristics of the study groups are described in Supplementary Table S2. Before surgery, fasting blood samples were obtained and used to measure alanine amino transferase (ALT), glucose, insulin, HDL cholesterol, LDL cholesterol and triglycerides. Insulin resistance was calculated using the homeostatic model assessment (HOMA-IR) index (Wallace *et al*, 2004). Surgical liver biopsies were obtained during surgery, and no ischemic preconditioning had been performed. Histopathological analysis was performed to evaluate hepatic steatosis (0, < 5%; 1, 5–30%; 2, > 30–60%; 3, > 60%).

Total liver RNA was extracted from human tissues using the RNeasy Mini Kit (Qiagen, Contraboeuf, France) and treated with Turbo DNA free (Applied Biosystems, Contraboeuf, France) following the manufacturer's protocol. The quantity and quality of the RNA were determined using the Agilent 2100 Bioanalyser with RNA 6000 Nano Kit (Agilent Technologies). Total RNA (1 μg) was reverse-transcribed with a High-Capacity cDNA Reverse Transcription Kit (Applied Biosystems). Real-time quantitative PCR was performed in duplicate for each sample using the ABI Step One Fast Real-Time PCR System (Applied Biosystems) as previously described (Kleiner *et al*, 2005). TaqMan gene expression assays were purchased from Applied Biosystems: RPLP0 (Hs99999902_m1) and TP53INP1 (Hs01003820_m1). Gene expression was normalized to the housekeeping gene human *RPLP0* (ribosomal phosphoprotein large P0) calculated based on the comparative cycle threshold *C*t method (2^−ΔΔ*C*t^). Results are expressed as the mean ± SEM. Statistical significance of differential human gene expression between two study groups was determined using the non-parametric Mann–Whitney *U*-test with the Δ*C*t of each group. Correlations were analyzed using the Spearman's rank correlation test. *P* < 0.05 was considered as significant.

### Study approval

Care and manipulation of mice were performed in accordance with national and European legislation on animal experimentation and were approved by the Aix-Marseille University Institutional Animal Care and Use Committee. Regarding human study, all subjects gave their informed written consent to participate in this study in accordance with French legislation regarding Ethics and Human Research (Huriet-Serusclat law). The ‘Comité Consultatif de Protection des Personnes dans la Recherche Biomédicale de Nice’ approved the study (07/04:2003, No 03.017).

**The paper explained**

**Problem**

The gene encoding tumor protein 53-induced nuclear protein 1 (TP53INP1) is a target of the tumor suppressor p53 and displays itself a tumor suppressor activity. TP53INP1 contributes to maintain cellular homeostasis in response to stress, in synergy with p53. Interestingly, the tumor suppressor activity of TP53INP1 is associated with its antioxidant function, shedding light in the link between the oxidative stress response and carcinogenesis. In addition, (i) TP53INP1 is involved in autophagy, a crucial catabolism process for cell homeostasis, and (ii) a genome-wide association study (GWAS) published in 2010 identified *TP53INP1* as a new type 2 diabetes susceptibility locus, but a pathological mechanism was not identified. In this context, this study further investigates the importance of TP53INP1 in cell metabolism regulation, associated with its implication in autophagy and redox homeostasis.

**Results**

In this paper, we demonstrate that *TP53INP1* is involved in the prevention of redox-driven obesity, which promotes insulin resistance, and thus type 2 diabetes. Furthermore, we unveil the impact of TP53INP1 on mitochondrial homeostasis, both quantitatively and qualitatively, as well as the central role of mitophagy alterations in chronic oxidative stress observed in the absence of TP53INP1. Thus, we provide a mechanism of TP53INP1 function in redox-dependent metabolism homeostasis.

**Impact**

TP53INP1 is a novel molecular actor in obesity and type 2 diabetes, thus being a promising target in prevention or therapy of metabolic syndrome. Thus, this study provides a useful preclinical model of mice prone to metabolic alterations.

## Supporting Information

Source Data for Supplementary Figure S6

Review Process File
