# Supplementary material for: Defects in mitophagy promote redox-driven metabolic syndrome in the absence of TP53INP1
Source: EMBO Mol Med. 2015 Mar 31;7(6):802–18. doi: 10.15252/emmm.201404318 (PMC4459819; doi:10.15252/emmm.201404318)
Supplement: Supplementary file 1 [file emmm0007-0802-sd1.pdf]

Seillier M *et al*.

"Defects in mitophagy promote redox-driven metabolic syndrome in the absence of TP53INP1"

Supplementary items

- **Supplementary Figures : 8**
- **Supplementary Tables : 2**

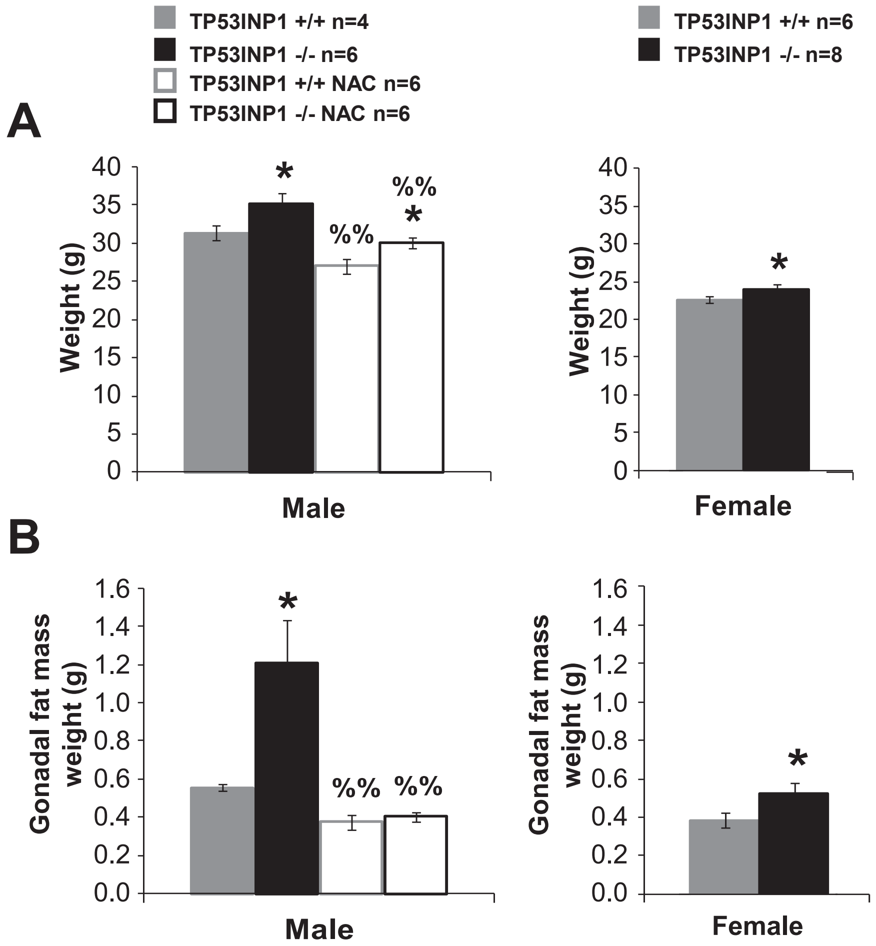

Figure S1. Male and female TP53INP1-deficient mice have higher body weight and gonadal fat mass than WT owing to their metabolic stress. (A) Five-month-old TP53INP1-deficient and WT mice (males and females) which drank during tap water supplemented or not with NAC for three months (10 mg/ml) were weighed. Male: p(-/- vs. +/+; CTRL) = 0.013 ; p(-/- vs. +/+; NAC) = 0.010 ; p(NAC vs. no NAC; +/+) = 0.004 ; p(NAC vs. no NAC; -/-) = 0.003. Female: p(-/- vs. +/+) = 0.039. **(B)** Gonadal fat mass was taken after sacrifice and weighed. Male: p(-/- vs. +/+; CTRL) = 0.013 ; p(NAC vs. no NAC; +/+) = 0.002 ; p(NAC vs. no NAC; -/-) = 0.004. Female: p(-/- vs. +/+) = 0.024. Results are expressed as the mean±S.E.M, and are representative of two independent experiments. *: -/- vs. +/+; %: NAC vs. no NAC; 1 character: p<0.05; 2 characters: p<0.005.

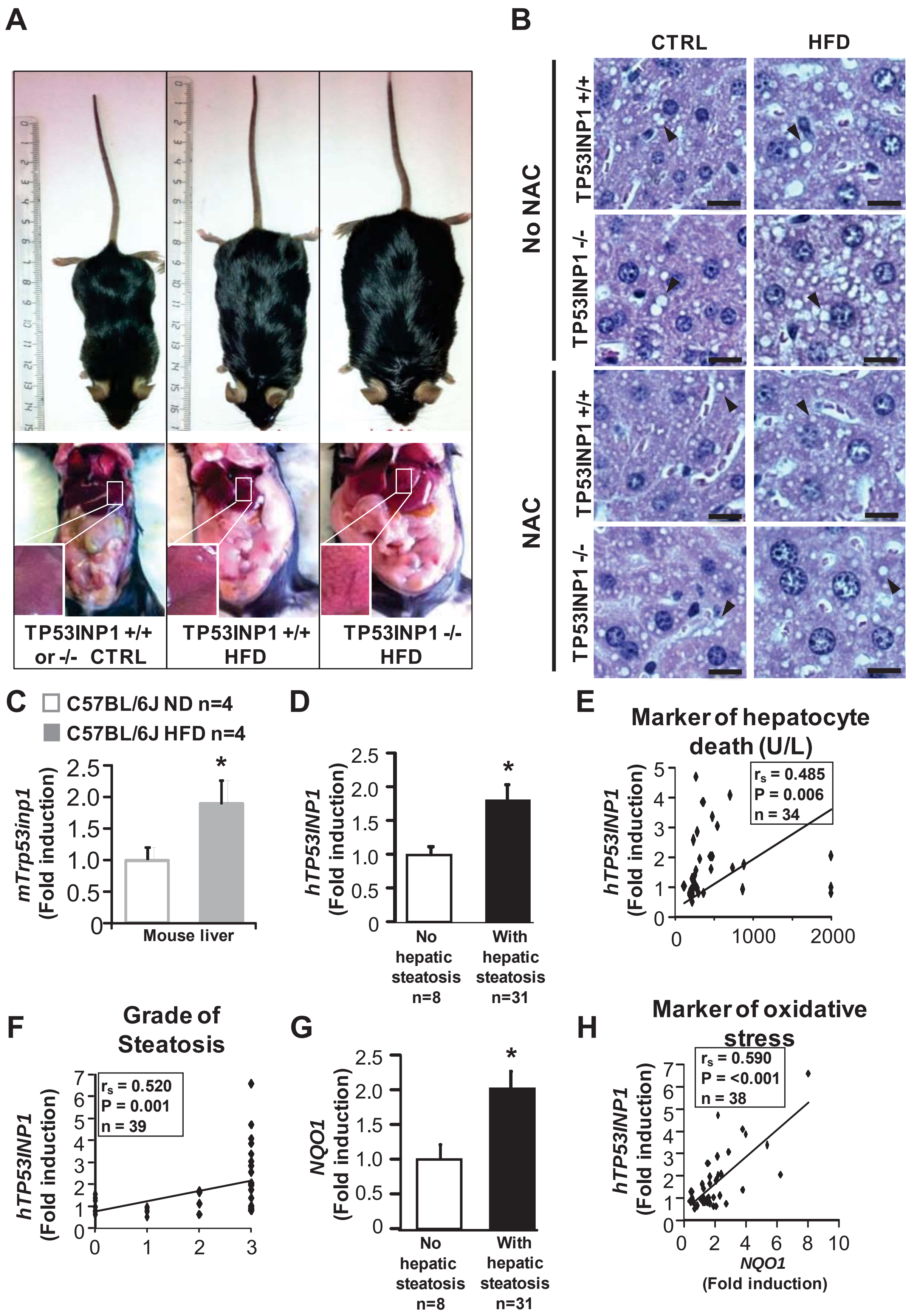

A
B
CTRL
HFD
No NAC
NAC
TP53INP1 +/+
TP53INP1 -/-
TP53INP1 +/+ or -/- CTRL
TP53INP1 +/+ HFD
TP53INP1 -/- HFD
C
C57BL/6J ND n=4
C57BL/6J HFD n=4
mTrp53inp1 (Fold induction)
Mouse liver
D
hTP53INP1 (Fold induction)
No hepatic steatosis n=8
With hepatic steatosis n=31
E
Marker of hepatocyte death (U/L)
rs = 0.485
P = 0.006
n = 34
F
Grade of Steatosis
rs = 0.520
P = 0.001
n = 39
G
NQO1 (Fold induction)
H
Marker of oxidative stress
rs = 0.590
P = <0.001
n = 38
NQO1 (Fold induction)

Figure S2. Absence of TP53INP1 favors hepatic steatosis, which is a stress accompanied with increased TP53INP1 expression in both mice and human. Male TP53INP1-deficient and WT mice were fed a High Fat Diet (HFD, 60% fat) or a control diet (CTRL) for 16 weeks (WT CTRL, n=6; KO CTRL, n=6; WT HFD, n=5; KO HFD, n=6). Mice drank tap water supplemented or not with NAC (10 mg/ml). TP53INP1-deficient mice were bigger and exhibited redox-dependent liver steatosis. **(A)** Mice were sacrificed at the end of the protocol and macroscopic observations were carried out on epididymal/renal fat masses and liver. **(B)** Formalin-fixed paraffin-embedded liver sections were stained by hematoxylin-eosin in order to observe steatosis level in each cohort. Arrow head = lipid droplet; nucleus are stained blue. Scale bar represents 0.5µm. Results are representative of three independent experiments. **(C)** Quantitative PCR for murine *Trp53inp1* mRNA levels in liver extract of C57BL/6J mice fed with a normal diet (5% fat; ND) or an High Fat Diet (45% fat; HFD) for 25 weeks. In HFD-fed mice, overexpression of TP53INP1 is also correlated with liver steatosis. Results are expressed as the mean±S.E.M and are representative of two independent experiments. *: p=0.031. **(D-H)** In human, morbidly obese patients with hepatic steatosis show an increase in hepatic TP53INP1 expression (*: p=0.031) **(D)**, and TP53INP1 expression is correlated with the level of a marker of hepatocyte death (keratin 18) **(E)** and with the grade of steatosis **(F)**. **(G-H)** Evaluation by qPCR of the hepatic level of NAD(P)H Dehydrogénase Quinone 1 (NQO1) as an indirect indicator of oxidative stress shows induction of NQO1 expression level in patients with hepatic steatosis (*: p=0.024) **(G)**. This induction of NQO1 is also correlated with induction of TP53INP1 **(H)**. Results are expressed as the mean±S.E.M.. rs= Spearman's rank correlation coefficient; P= p-value; n= number of patients.

Altogether, those data show that accumulation of lipids in steatotic liver is correlated with induction of stress response proteins such as the antioxidant NQO1 and TP53INP1. Expression of TP53INP1 is increased in most of stress settings that our laboratory assessed in the past. TP53INP1 is a player in stress resolution. In its absence (TP53INP1 -/- mice), hepatic cells miss TP53INP1 to cope with stress thus hepatic steatosis is promoted.

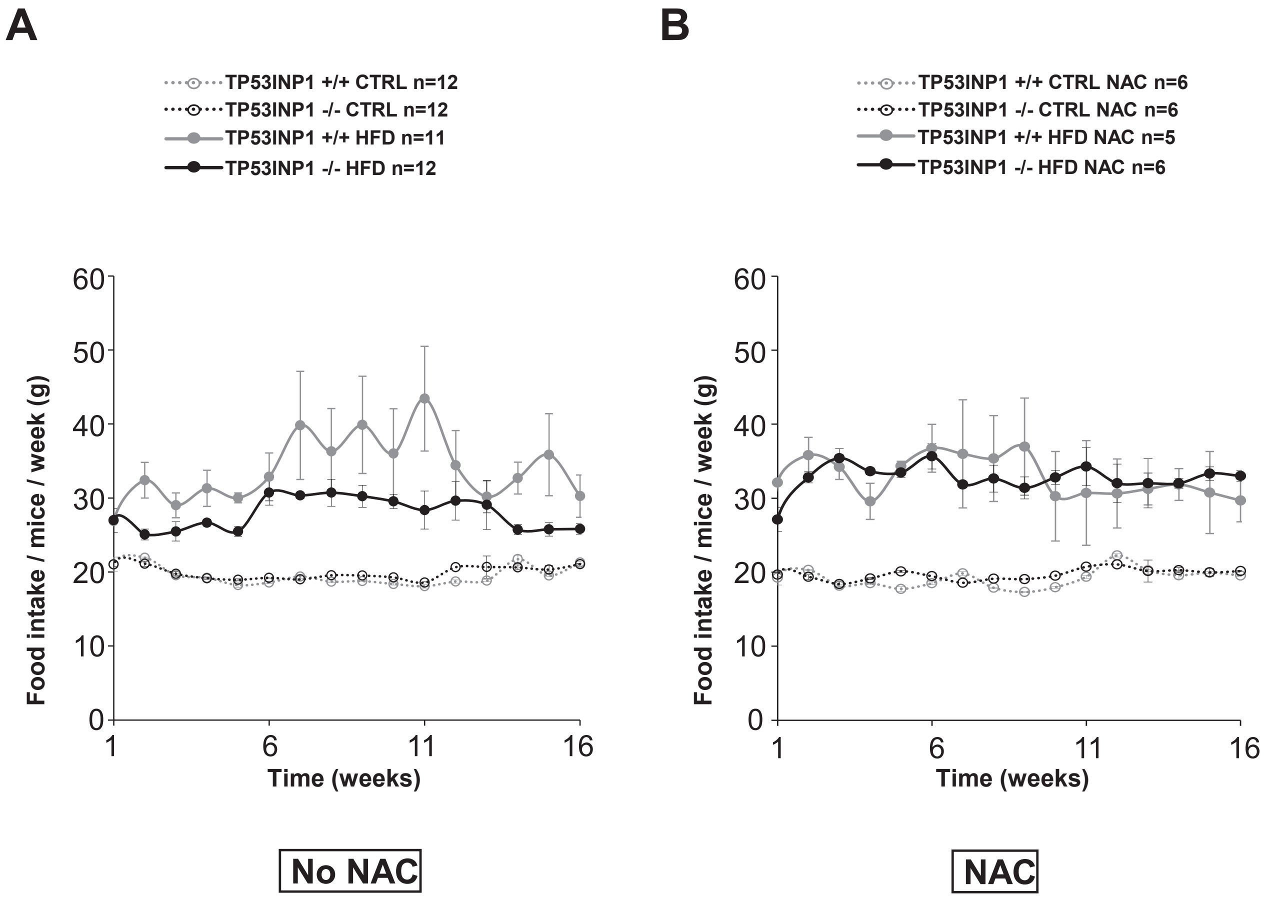

Figure S3. TP53INP1-deficient mice eat as much as WT mice, even when treated with NAC. Male TP53INP1-deficient and WT mice were fed a High Fat Diet (HFD, 60% fat) or a control diet (CTRL) during 16 weeks. Mice drank tap water **(A)** or tap water with NAC **(B)**. Food was weighed every week: curves show food intake per mouse per week. Results are expressed as the mean±S.E.M, and are representative of three independent experiments. Differences are statistically non-significant according to Student's t test.

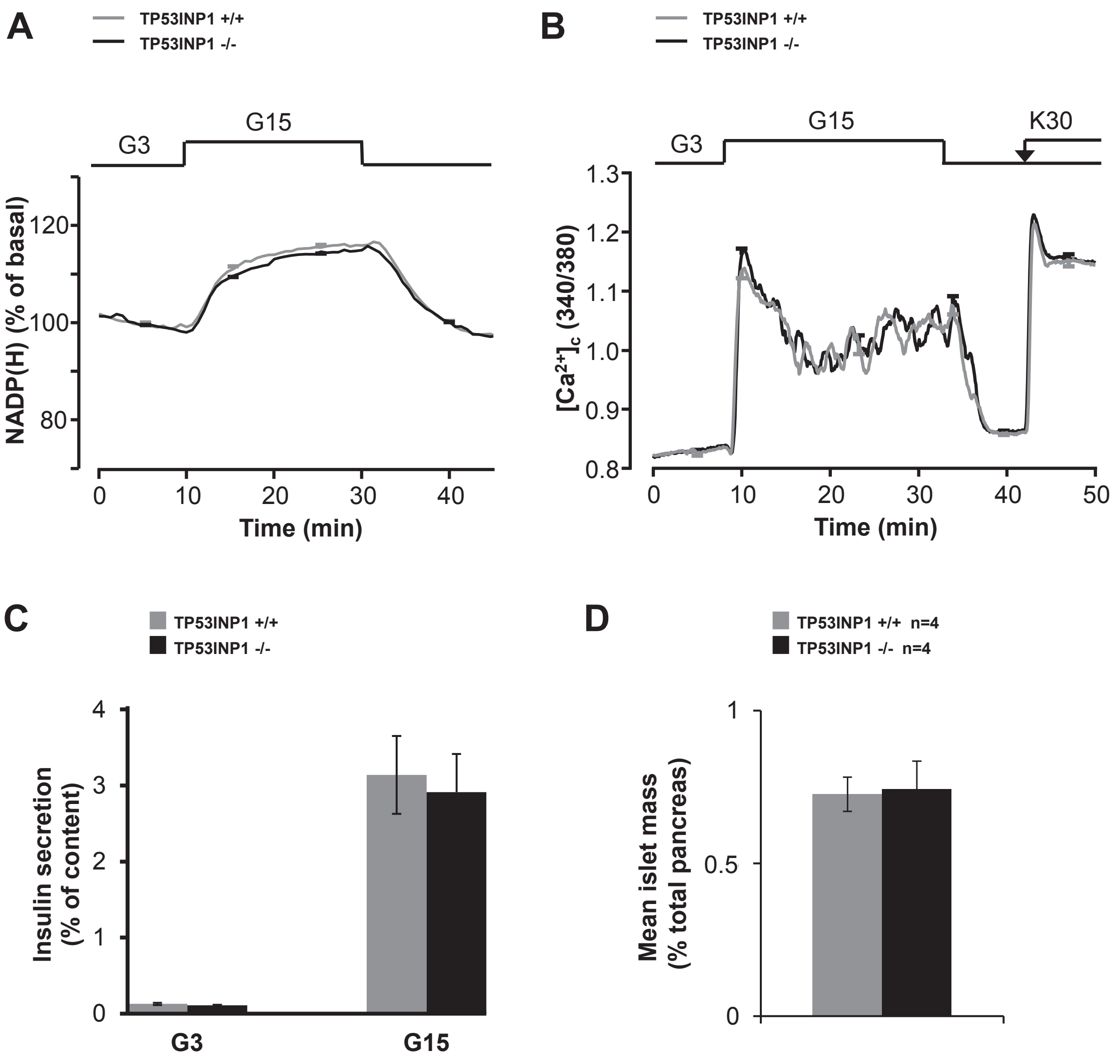

Figure S4. TP53INP1-deficient mice do not display any defect in glucose-induced metabolic changes in islet ββ-cells, nor in insulin secretion or islet mass. NADP(H) changes **(A)**, reflecting changes in glucose metabolism, and $[Ca^{2+}]_c$ changes **(B)** were monitored in islets isolated from TP53INP1 KO and WT mice. The islets were perifused with 3 mM glucose (G3) and stimulated with 15 mM glucose (G15) as indicated. Results are expressed as the mean±S.E.M of 17-21 islets from three independent experiments/mice. **(C)** Insulin secretion of isolated islets from TP53INP1 KO and WT mice incubated during 30 min in 3 mM or 15 mM glucose as indicated. **(D)** Pancreases isolated from 3-month-old WT and TP53INP1 KO mice were used for histomorphometric studies. Islet mass was determined using 4μm pancreatic sections which were labelled with eosin and hematoxylin and scanned using the NDPview software. Results are expressed as the mean±S.E.M, and are representative of two independent experiments.

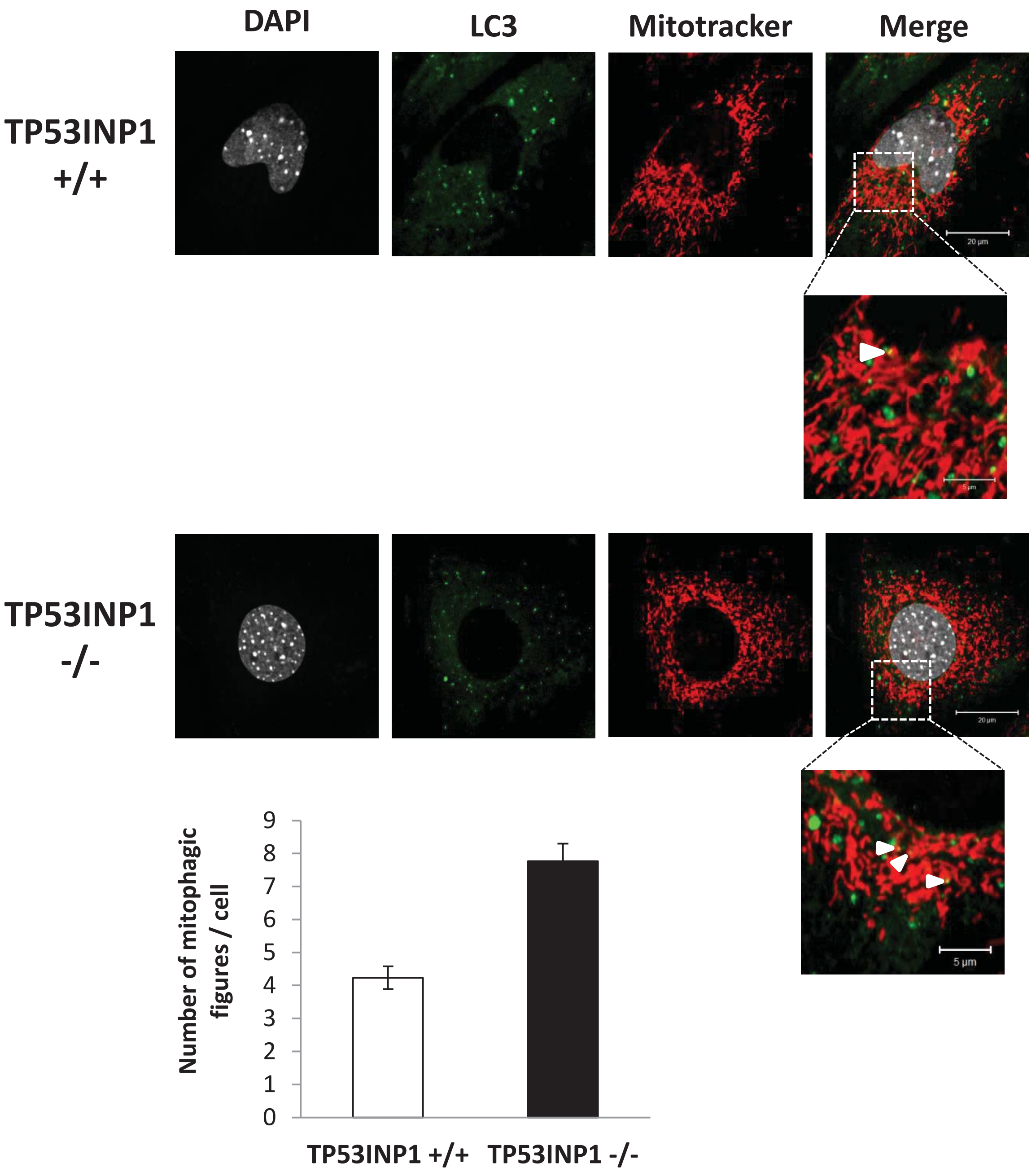

Figure S5. TP53INP1-deficient MEFi contain a higher number of mitophagic structures than WT. Cells were co-stained with an anti-LC3 antibody and Mitotracker and analyzed on a confocal fluorescence microscope. Mitotracker-LC3-positive puncta (yellow dots = mitophagic structures) are depicted with a white arrow in the high-magnification images. Mitophagic structures were carefully quantified on high magnification images (10 cells per genotype) in three independent experiments (30 total cells per genotype). Quantification histogram shows the mean±S.E.M. Scale bar represents 20 µm (5 µm for high-magnification images).

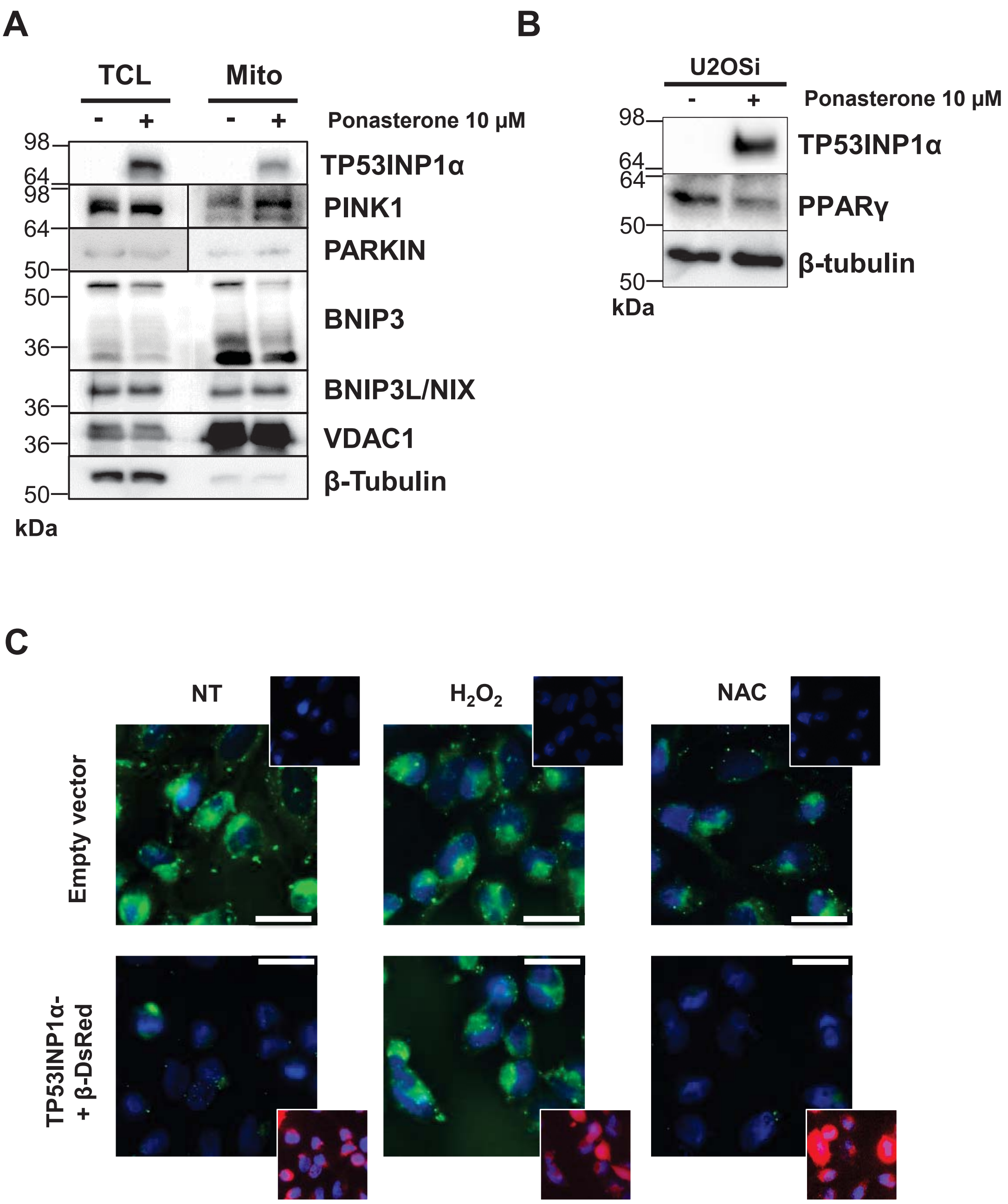

Figure S6. Restoration of TP53INP1 expression in U2OS cells. (A) U2OSi were induced or not for TP53INP1α-GFP expression by addition of ponasterone (10 μM) in growth medium 24h before experiments. Mitochondrial lysates (Mito) were purified from total cell lysates (TCL) and both were analyzed by immunoblotting for TP53INP1, PINK1, PARKIN, BNIP3, BNIP3L/NIX, VDAC1 and β-tubulin. **(B)** TCLs from U2OSi, induced or not by ponasterone A (10 μM), were analyzed by immunoblotting for PPARγ and β-tubulin. **(C)** U2OSi were seeded in media containing or not 10mM NAC. Twenty-four hours later, U2OSi were transfected with TP53INP1α and β-DsRed-N1 vector (bottom images) or empty vector (upper images). After 24h, U2OSi were treated or not (NT) with 1 mM hydrogen peroxide (H_2O_2) during 1h. Cells were left to recover for 4h in normal media containing NAC (10mM) or not, and then harvested. Cells were observed by fluorescence microscopy after Bodipy493/503 staining of LD (green) and DAPI staining of nuclei (blue). TP53INP1α or β-DsRed expression was evaluated by measuring Ds-Red fluorescence (red, small insert). Scale bar represents 100 μm. Results are representative of three independent experiments.

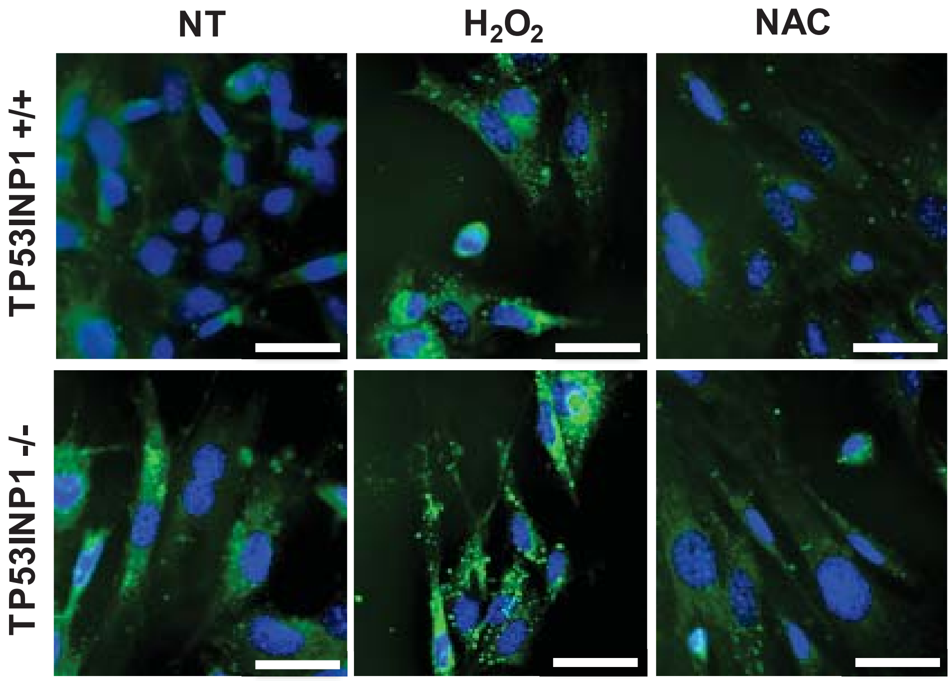

Figure S7. Oxidative stress observed in the absence of TP53INP1 is at the origin of the presence of lipid droplets (LD) in primary MEF. Primary MEF (MEFp) deficient (-/-) or not (+/+) for TP53INP1 were seeded in media containing or not 10mM NAC. Fourty-eight hours later, MEFp were treated or not (NT) with 1 mM hydrogen peroxide (H_2O_2) for 1h. Cells were left to recover for 4h in normal media containing NAC (10mM) or not, and then harvested. Cells were observed by fluorescence microscopy after Bodipy493/503 staining of LD (green) and DAPI staining of nuclei (blue). Scale bar represents 100 µm. Results are representative of two independent experiments.

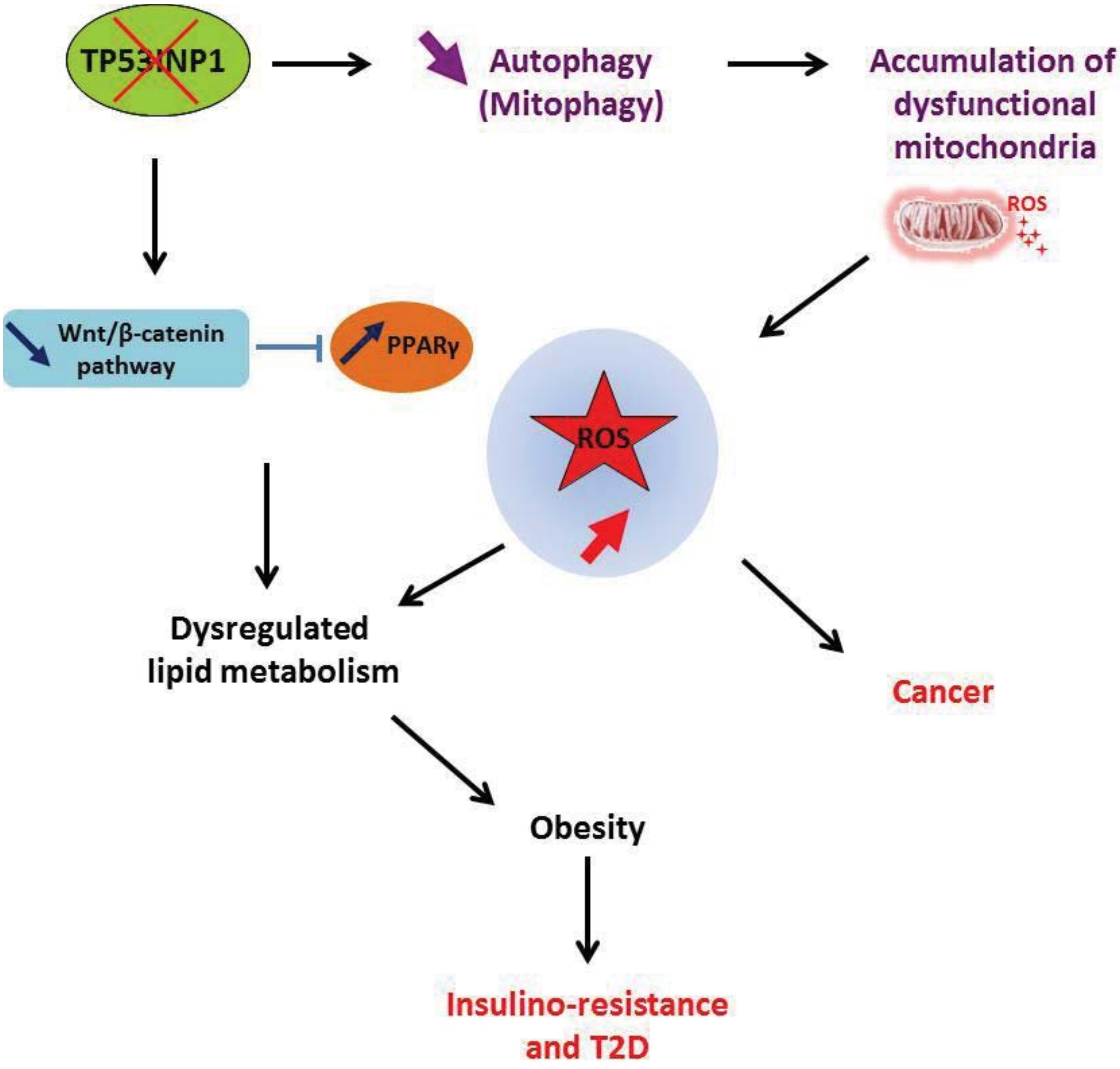

Figure S8. Graphical abstract. TP53INP1 is a protein with tumor suppressive activity due to its role in redox control. We show in this paper that TP53INP1 also plays a role in T2D prevention by regulating redox-associated lipid metabolism. Its implication in redox control stems from its involvement in autophagy, in particular mitophagy which contributes in the homeostasis of mitochondria, the main cellular source of ROS.

Table S1. HOMA-IR of mice in HFD protocol

Group	HOMA-IR		p
	+/+	-/-	
CTRL	3.12 ± 1.43	1.74 ± 0.51	0.197
HFD	11.12 ± 3.14	36.77 ± 9.98	0.025
CTRL NAC	2.52 ± 0.68	3.16 ± 1.21	0.326
HFD NAC	10.28 ± 2.13	9.85 ± 1.71	0.440

HOMA-IR: Homeostasis Model Assessment of Insulin Resistance. HOMA-IR is calculated according to this formula : fasting-glycemia (mg/dL) x fasting-insulinemia (mUI/L) / 405. Data are expressed as mean± S.E.M and compared using the parametric Student's t test.

Table S2. Characteristics of obese patients

	Without hepatic steatosis	With hepatic steatosis	P
n	8	31	
Age (years)	37.8 ± 4.6	37.8 ± 1.9	0.930
Sexe (F/M)	7/1	21/10	
BMI (kg/m²)	42.9 ± 0.6	44.1 ± 1.0	0.861
ALT (IU/L)	17.9 ± 3.8	50.6 ± 9.9	0.004
Fasting insulin (mIU/L)	11.0 ± 2.6	23.5 ± 4.15	0.115
Fasting glucose (mmol/L)	4.8 ± 0.1	5.7 ± 0.2	0.007
HOMA-IR	2.3 ± 0.5	6.2 ± 1.1	0.048
HbA1c (%)	5.3 ± 0.1	5.8 ± 0.1	0.097
Triglycerides (mmol/L)	0.98 ± 0.14	2.23 ± 0.36	0.001
HDL cholesterol (mmol/L)	1.71 ± 0.12	1.32 ± 0.07	0.005
LDL cholesterol (mmol/L)	2.38 ± 0.54	3.24 ± 0.15	0.027
Grade of steatosis (n)	0(8)	1(5)/2(5)/3(21)	≤0.001

Without hepatic steatosis: obese patients with normal liver histology. With hepatic steatosis: obese patients with steatosis. Data are expressed as mean±S.E.M and compared using the non-parametric Mann Witney test.
