# Supplementary material for: Defects in mitophagy promote redox-driven metabolic syndrome in the absence of TP53INP1
Source: EMBO Mol Med. 2015 Mar 31;7(6):802–18. doi: 10.15252/emmm.201404318 (PMC4459819; doi:10.15252/emmm.201404318)

# *Uncropped gels used for Figure S6.A*

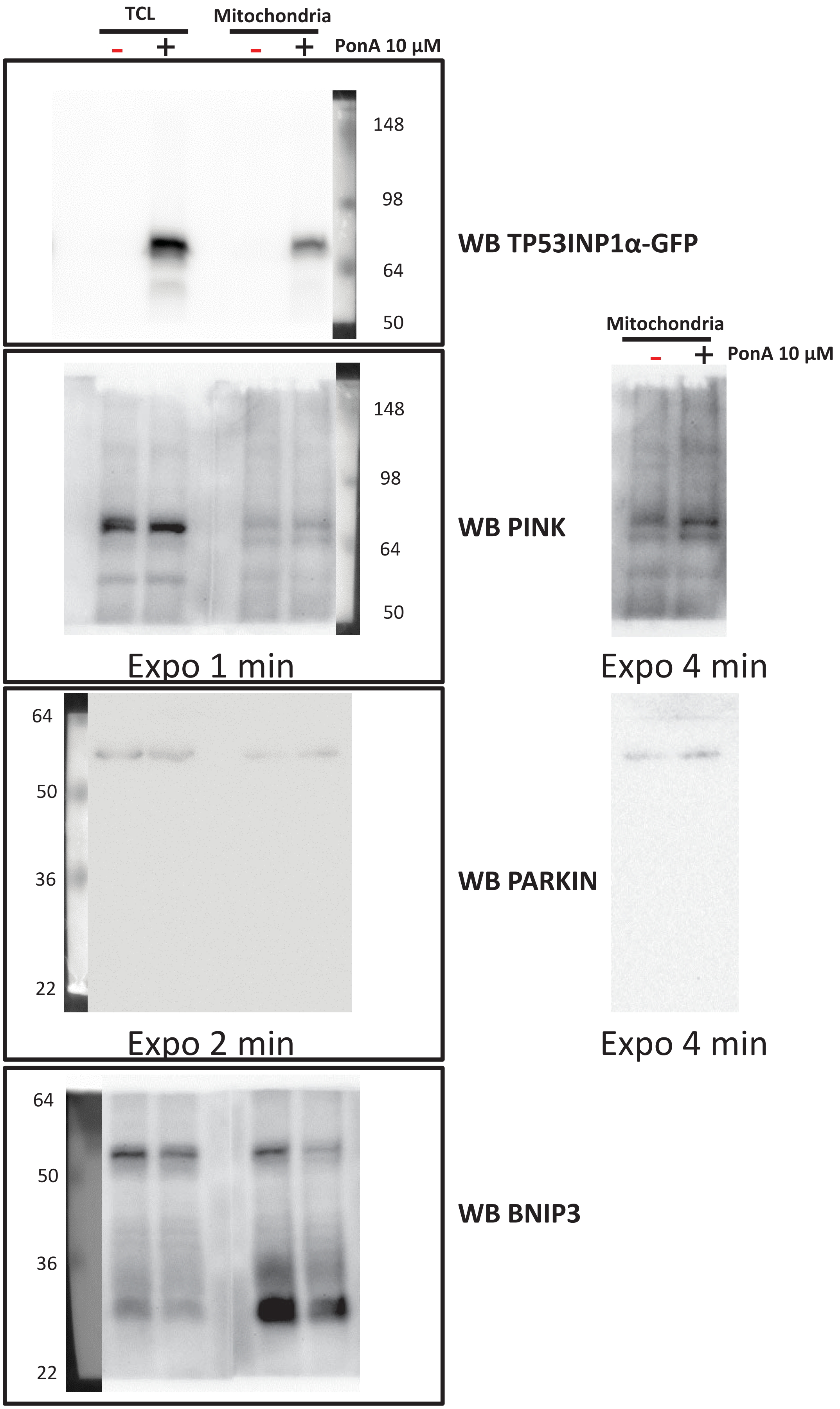

# ***Uncropped gels used for Figure S6.A***

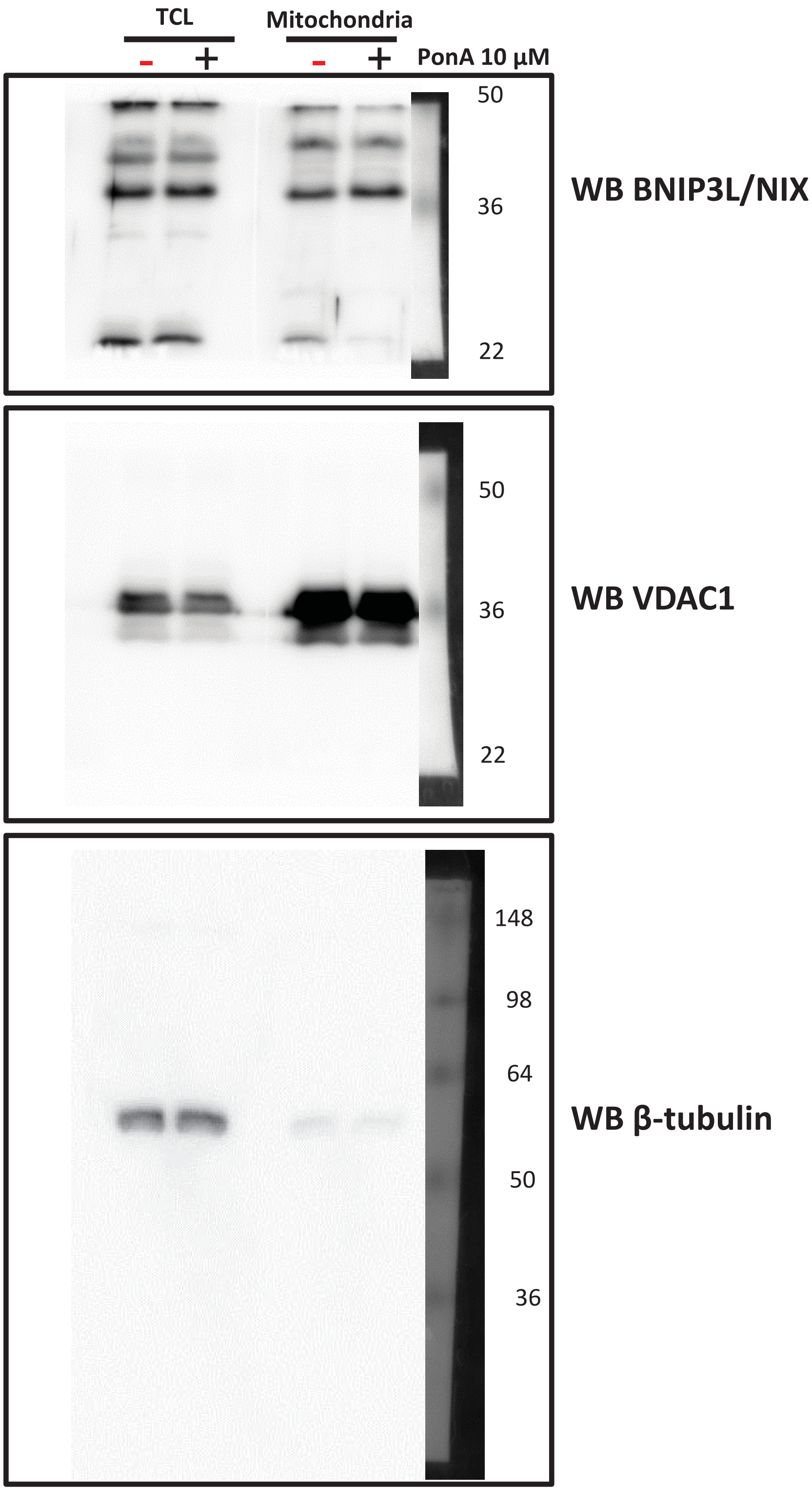

# *Uncropped gels used for Figure S6.B*

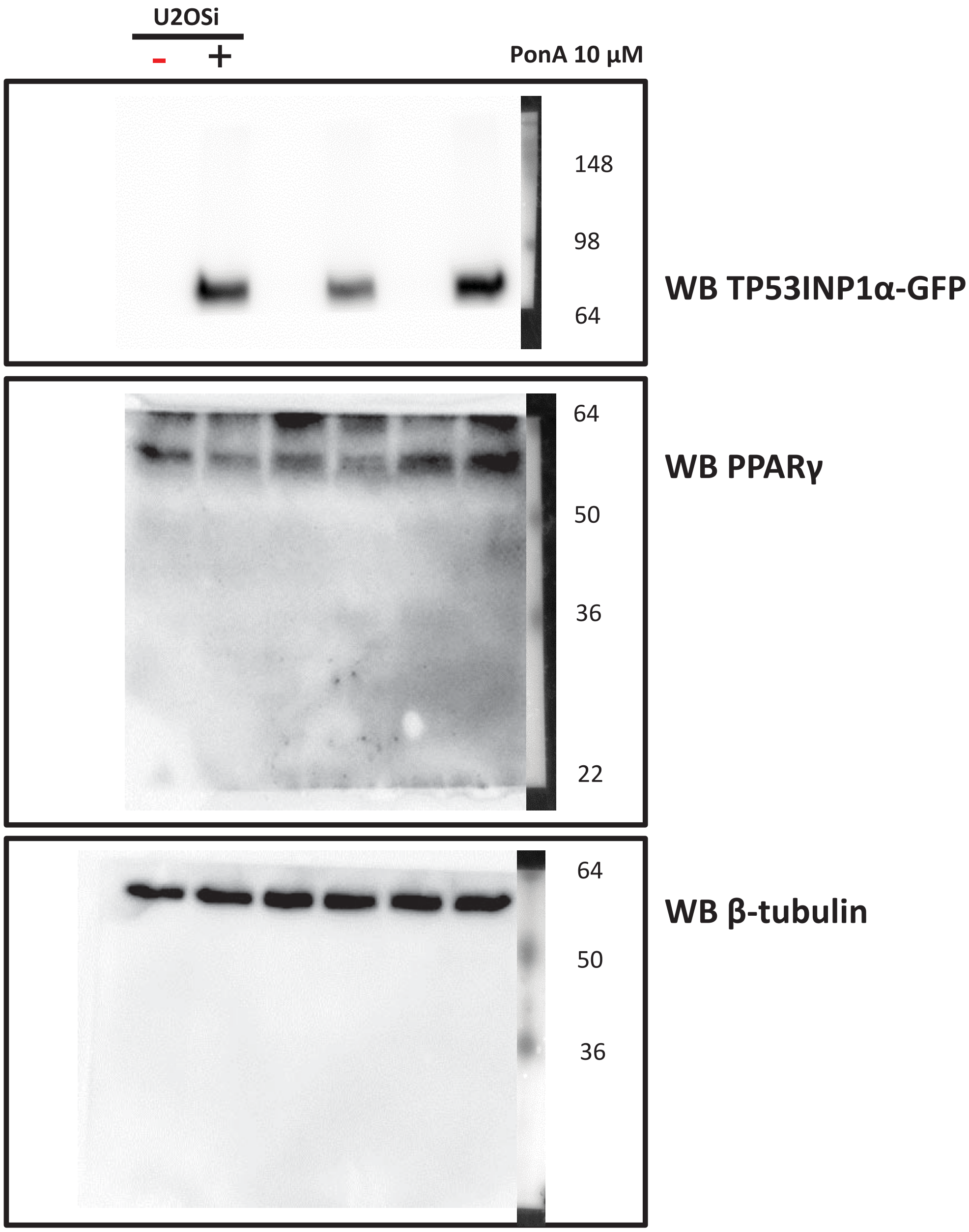

Supplement: Supplementary file 2 [file emmm0007-0802-sd2.pdf]
