# Supplementary material for: Defects in mitophagy promote redox-driven metabolic syndrome in the absence of TP53INP1
Source: EMBO Mol Med. 2015 Mar 31;7(6):802–18. doi: 10.15252/emmm.201404318 (PMC4459819; doi:10.15252/emmm.201404318)
Supplement: Supplementary file 3 [file emmm0007-0802-sd3.pdf]

Defects in mitophagy promote redox-driven metabolic syndrome in the absence of TP53INP1

Marion Seillier, Laurent Pouyet, Prudence N'Guessan, Marie Nollet, Florence Capo, Fabienne Guillaumond, Laure Peyta, Jean François Dumas, Annie Varrault, Gyslaine Bertrand, Stéphanie Bonnafous, Albert Tran, Gargi Meur, Piero Marchetti, Magalie A. Ravier, Stéphane Dalle, Philippe Gual, Dany Muller, Guy A. Rutter, Stéphane Servais, Juan L. Iovanna and Alice Carrier

Corresponding author: Alice Carrier, Cancer Research Center of Marseille CRCM

Review timeline:

Submission date:	03 June 2014
Editorial Decision:	27 June 2014
Revision received:	17 November 2014
Editorial Decision:	18 December 2014
Revision received:	02 February 2015
Editorial Decision:	04 March 2015
Accepted:	09 March 2015

Transaction Report:

(Note: With the exception of the correction of typographical or spelling errors that could be a source of ambiguity, letters and reports are not edited. The original formatting of letters and referee reports may not be reflected in this compilation.)

Editor: Céline Carret

1st Editorial Decision 27 June 2014

Thank you for the submission of your manuscript to EMBO Molecular Medicine. We have now heard back from the three referees whom we asked to evaluate your manuscript. Although the referees find the study to be of potential interest, they also raise a number of concerns that should be addressed in the next final version of the manuscript.

As you will see from the comments below, the three referees are enthusiastic about the study but do have suggestions and recommendations to further improve conclusiveness and clarity as well as increase the potential clinical aspect, which is particularly important for your scope. All three referees recommend improving the mechanistic insights and provide clear and nicely detailed suggestions to do so. In addition, particularly referees 1 and 3 are concerned about the human data that do not seem to match the mouse findings and we would strongly recommend to address this issue as best as you possibly can.

Given these evaluations, I would like to give you the opportunity to revise your manuscript, with the understanding that the referees' concerns must be fully addressed and that acceptance of the manuscript would entail a second round of review. Please note that it is EMBO Molecular Medicine policy to allow only a single round of revision and that, as acceptance or rejection of the manuscript will depend on another round of review, your responses should be as complete as possible.

I look forward to receiving your revised manuscript.

***** Reviewer's comments *****

Referee #1 (Comments on Novelty/Model System):

The findings are novel as they provide the first biological link between TP53INP1 loss and increased metabolic syndrome. The potential causative link with chronic ROS is also novel and potentially clinically tractable. As the manuscript stands, the mouse model is appropriate, but the link to human disease is tenuous at best. This arises from the scarcity of human data presented (limited to analysis of TP53INP1 mRNA levels in obese patients), but more critically, the discrepancy with the mouse model. In the patients, there are clearly higher levels of TP53INP1 mRNA associated with steatosis whereas in the mouse, the absence of TP53INP1 is linked to steatosis.

Referee #1 (Remarks):

The manuscript by Seiller et al., describes novel and potentially clinically-relevant findings regarding a role of the p53 target gene product TP53INP1 in ameliorating oxidative stress and contributing to protection from metabolic syndrome. Building upon previous findings form this group and others that TP53INP1 plays a role in mitophagy as well as GWA studies showing a link between loss of TP53INP1 and metabolic syndrome in obese individuals, the authors have generated a knockout mouse model which shows increased propensity for total weight gain (in male mice) and liver steatosis compared to WT mice. Critically, treating the mice with the antioxidant N-acetylcysteine (NAC) reverses chronic oxidative stress, but also overall and organ-specific weight gain in the TP53INP1-/- mice. It is also demonstrated that TP53INP1-/- mice have hyperglycemia and hyperinsulinemia. At the cellular level, the authors provide evidence for increased formation of lipid droplets in the cytoplasm of TP53INP1-/- mice which is exacerbated upon a high fat diet. In vitro, TP53INP1-/- MEFs demonstrate increased ROS levels which are accompanied by increased number of mitochondria and defective mitophagy. Data from human obese patients are far less convincing and in one respect, contradictory with the mouse model.
The main conclusion of this work-that TP53INP1plays a critical role in protecting form insulin resistance and T2D-would be significantly strengthened if the authors could provide additional information regarding the role of TP53INP1 in mitophagy and try to clarify some of their findings in human patients.

Major issues:

1. In the human patient data, the correlation between increased TP53INP1 mRNA levels in obese patients with steatosis vs. those with non-steatosis is confusing: If TP53INP1 is protective against chronic oxidative stress and metabolic syndrome, why are the levels of TP53INP1 higher in patients with steatosis? The authors' statement (middle of p.5) that "in humans, hepatic TP53INP1-expression was upregulated with hepatic steatosis and correlated with liver injury...supporting our results on mice model is adding to the confusion, since in the mice, loss of TP53INP1 is linked to increased steatosis (Figs. E2A,B). This needs to be explained. What do the protein levels of TP53INP1 look like in these two patient cohorts? The correlation with ALT is barely statistically significant and not very convincing.
2. The link between loss of TP53INP1 and defective mitophagy/increased mitochondrial numbers requires additional supporting data. First, in addition to staining with mitochondria-specific dyes (mito-Green and mito-Red), the authors need to perform quantitative analysis of mitochondrial DNA and normalize to nuclear DNA. This can be readily done by amplifying mtDNA-specific genes (e.g., COX-I) and nuclear-specific genes. Moreover, co-localization of mitotracker dyes with GFP-LC3 or any other autophagy-specific marker would be more convincing to demonstrate mitophagy processing.
3. Even if there's defective mitophagy, there is no definitive evidence that this is the sole rason for increased ROS. There could also be increased mitochondrial biogenesis in TP53INP1-deficient cells (albeit these mitochondria would have abnormal OXPHOS). The levels of the major mitochondrial biogenesis regulator, PGC-1 should be analyzed as it could a target of repression/degradation by TP53INP1.
4. The in vitro experiments with MEFi showed convincingly that the TP53INP1-/- MEFs have higher basal and high-lipid induced levels of ROS and lipid droplets. This could be due to indirect effects induced

during development. Demonstrating that expression of TP53INP1 in trans in these cells can rescue these phenotypes, would provide unequivocal evidence for a more direct involvement of TP53INP1 in this process.
5. Why do the male TP53INP1 KO mice exhibit a much more profound phenotype in terms of HFD weight gain that female KO mice? This is never discussed. Also, since the phenotype is more severe in the male mice, why are the TP53INP1 mRNA levels analyzed in female mice in Fig. 3C
6. In figure 3A, it is unclear what is shown. Is this a picture of a single pancreas from one mouse or beta-cells in culture? In any case, there is no obvious co-localization of TP53INP1(red) and insulin (green).
7. Why are the levels of TP53INP1 much lower in the islets of mice and rats (Fig. 3B and 3C) compared to the exocrine pancreas and other tissues (e.g., spleen?). One would expect that the islets would express higher levels, which the authors report on p 7.
Minor points:
a. What is "oxygen flow", mentioned on p9 (first paragraph)? From the description of the experiment and the methods section, it appears that the authors are actually measuring oxygen consumption? If that's the case, the term should be corrected.
b. There are several misspellings/grammar errors in the manuscript:
i. P.5: "...histological analysis was more important in KO that in WT mice..." should read: "...histological analysis was more pronounced in KO that in WT mice..."
ii. P.6: "...predisposes them to overweight..." should read "... predisposes them to inceased weight gain...".
iii. P. 12, second par: "Consistently with our previous works" should read ":consistent with our previous work".
iv. P31: "...males mice were submitted to a..." should read "male mice were subjected to...".

Referee #2 (Remarks):

The authors have analyzed in the present manuscript the role of TP53INP1 in the propensity to obesity-related development of type-2 diabetes, with the help of a mouse KO model. The authors convincingly show that TP53INP1-deficient mice are prone to obesity and hepatic steatosis and that this is due to oxidative stress accumulation. They further strongly demonstrate that their KO mice develop type 2 diabetes, proving the value of their model for disease studies. This study represents another successful example of GWASs usage to unravel disease mechanisms. However, the subsequent mechanistic analysis is rather descriptive (as also reflected in their abstract) and could be improved. How does TP53INP1 prevent oxidative stress? Is mitophagy in general affected or is there a specific effect on the PINK1/PARKIN pathway?

Major comments:
To prove an effect of TP53INP1 in mitophagy, in PINK1/PARKIN levels and in lipid droplet accumulation, rescue experiments by re-expression of the TP53INP1 protein must be performed.
The role of TP53INP1 in mitophagy should be further substantiated and quantified.
To bring an insight into the molecular regulation at game, the authors should investigate the subcellular localization of the TP53INP1 protein, especially in presence and absence of both oxidative stress and lipid stress. Furthermore, the importance of their analysis of TP53INP1 expression in beta-cells (Fig.3) is unclear to this reviewer, because insulin was anyway present in the plasma (Fig. 2B). If, then, they could compare it to the mRNA expression in target tissues, in the presence and absence of HFD and NAC.
The authors could overexpress PINK1 and PARKIN in TP53INP1 KO cells and then look for a possible rescue of their phenotypes. How does TP53INP1 regulate PINK1/PARKIN? Is there a physical interaction? Parkin subcellular localization in wt and TP53INP1 KO cells could be investigated using microscopy immunostaining or WB analysis after subcellular fractionation.
They observed an impressive and convincing increased mitochondrial mass and ROS production in absence of TP53INP1, effect mimicked, at least in part, by autophagy inhibition (Fig. 4). They then confirm the presence of autophagic vacuoles containing mitochondria in TP53INP1 KO cells, unaffected by H2O2 treatment (Fig. 5A). This brings the authors to conclude that the observed oxidative stress is a consequence and not a cause of impaired mitochondria degradation. However, this assumes that H2O2 treatment equals TP53INP1 KO in terms of oxidative stress and mitochondrial mass effects. Therefore, ROS production and

mitochondrial mass should be analysed in wt and KO cells, with or without H2O2 and all 4 conditions with or without 3MA.

Minor comments:
Fig.5A remove red points and rather sign mitochondria without covering them.
The subcutaneous fat could also be provided.
The unpublished data mentioned on top of page 7 demonstrating no defects in insulin secretion or islet mass could be included.

Referee #3 (Comments on Novelty/Model System):

The model is of high interest as the authors generate mice deficient in TP53INP1 to dissect its role in metabolic diseases induced by HFD.

Referee #3 (Remarks):

The study of Seillier et al, aims to investigate the role of TP53INP1, a p53 regulated protein with antioxidant properties with tumor suppressive functions, in metabolic syndrome induced by feeding a high fat diet (HFD). For this end authors generate TP53INP1 knockout mice. The data show that KO mice fed HFD gain more body weight, and exhibits more adiposity and hepatomegaly compared to WT mice. KO mice also exhibit worse glucose intolerance and insulin resistance than WT mice and they are also more sensitive to HFD-induced hepatic steatosis than WT mice. All these effects induced by HFD in the KO mice are blunted by NAC administration, while NAC given to WT mice fed HFD does not cause any beneficial effect Mitochondria from KO mice generate more superoxide (mito-sox) and exhibit increased mitochondrial mass compared to WT mice. Work with MEFi indicates that the lack of TP53INP1 increases mitochondria number and size and exhibit decreased oxygen consumption determined by high resolution respirometry, and exhibit reduced Parkin/Pink1 levels, indicative of impaired mitophagy. These findings suggest that in the absence of TP54INP1 mitophagy is impaired contributing to an increase population of dysfunctional mitochondria that generate ROS and this event determines a worse progression towards obesity, fatty liver disease (steatosis) and insulin resistance.

COMMENTS

While the study is of interest and potential relevance, there are a number of concerns that need to be further addressed regarding the mechanisms of TP53INP1 regulation of mitochondrial function and impact in metabolic syndrome.

1. What of the basic questions in to establish is whether TP53INP1 is actually a mitochondrial-targeted protein. Does TP53INP1 regulate mitochondrial respiratory complex assembly and/or function.

2. Is there any defect in the mitochondrial fatty acid b-oxidation capacity in the absence of TP53INP1. Any change in expression or activity of CTP-1.

3. Does the lack of TP53INP1 regulate mitochondrial antioxidant defenses, e.g. GSH, MnSOD, Prx-III, which could further contribute to the increased ROS examined. Is there also any change in hydrogen peroxide levels?

4. The authors do not show that NAC actually decrease the mt ROS determined by mitoSox in Figure 4. This should be established.

5. Does NAC revert the decrease in Parkin and Pink1 and therefore resumes mitophagy. This is critical to establish cause and effect relationship.

6. I believe all the work with MEFi are not particularly relevant to the overall aim. I think it would be more important to establish these events in primary mouse hepatocytes instead. Why H2O2 does not potentiate LD in TP53INP1 null cells (Figure 6B).

7. The data on human samples (Fig E2C, D) do not support the findings in the KO mice, and I disagree with such statement made in page 5 (last sentence in first paragraph). To be consistent with the mice data, I would expect lower TP53INP1 levels in samples from patines with steatosis.

8. Overall, the chronic ROS generation contributing to insulin resistance and obesity is only of relevance in the absence of TP53INP1 not in WT mice. This implies that antioxidants would not be of much value as potential therapy for metabolic syndrome. This issue should be discussed at the end.

1st Revision - authors' response 17 November 2014

EMM-2014-04318

" Defects in mitophagy promote redox-driven metabolic syndrome in the absence of TP53INP1 "

Response: We warmly thank the Reviewers and Editors for reading our manuscript carefully and for their suggestions. We provide responses to the issues raised by the three reviewers below. We believe we have fully addressed the referees' concerns, and provide responses that are as complete as possible. The revised work now provides the deeper mechanistic insights requested by the three referees and we feel has substantially improved our study.

The corrections and additional text appear in red in the revised manuscript.

<u>Response to Reviewers' comments:</u>

Referee #1 (Comments on Novelty/Model System):

The findings are novel as they provide the first biological link between TP53INP1 loss and increased metabolic syndrome. The potential causative link with chronic ROS is also novel and potentially clinically tractable. As the manuscript stands, the mouse model is appropriate, but the link to human disease is tenuous at best. This arises from the scarcity of human data presented (limited to analysis of TP53INP1 mRNA levels in obese patients), but more critically, the discrepancy with the mouse model. In the patients, there are clearly higher levels of TP53INP1 mRNA associated with steatosis whereas in the mouse, the absence of TP53INP1 is linked to steatosis.

Response: We completely agree with Reviewer #1. We provide below the answer to this major question (see Major issues # 1.).

Referee #1 (Remarks):

The manuscript by Seiller et al., describes novel and potentially clinically-relevant findings regarding a role of the p53 target gene product TP53INP1 in ameliorating oxidative stress and contributing to protection from metabolic syndrome. Building upon previous findings form this group and others that TP53INP1 plays a role in mitophagy as well as GWA studies showing a link between loss of TP53INP1 and metabolic syndrome in obese individuals, the authors have generated a knockout mouse model which shows

increased propensity for total weight gain (in male mice) and liver steatosis compared to WT mice. Critically, treating the mice with the antioxidant N-acetylcysteine (NAC) reverses chronic oxidative stress, but also overall and organ-specific weight gain in the TP53INP1-/- mice. It is also demonstrated that TP53INP1-/- mice have hyperglycemia and hyperinsulinemia. At the cellular level, the authors provide evidence for increased formation of lipid droplets in the cytoplasm of TP53INP1-/- mice which is exacerbated upon a high fat diet. In vitro, TP53INP1-/- MEFs demonstrate increased ROS levels which are accompanied by increased number of mitochondria and defective mitophagy. Data from human obese patients are far less convincing and in one respect, contradictory with the mouse model.
The main conclusion of this work-that TP53INP1plays a critical role in protecting form insulin resistance and T2D-would be significantly strengthened if the authors could provide additional information regarding the role of TP53INP1 in mitophagy and try to clarify some of their findings in human patients.

Major issues:

1. In the human patient data, the correlation between increased TP53INP1 mRNA levels in obese patients with steatosis vs. those with non-steatosis is confusing: If TP53INP1 is protective against chronic oxidative stress and metabolic syndrome, why are the levels of TP53INP1 higher in patients with steatosis? The authors' statement (middle of p.5) that "in humans, hepatic TP53INP1-expression was upregulated with hepatic steatosis and correlated with liver injury...supporting our results on mice model is adding to the confusion, since in the mice, loss of TP53INP1 is linked to increased steatosis (Figs. E2A,B). This needs to be explained. What do the protein levels of TP53INP1 look like in these two patient cohorts? The correlation with ALT is barely statistically significant and not very convincing.

Response: We thank Reviewer #1 for having highlighted the necessity to clarify the human investigation and its interpretation. Actually the focus of this present work was to take advantage of our unique TP53INP1 tools (mouse and cells models) to gain insights into the cellular and molecular role of TP53INP1 in metabolism as a new T2D gene. As obesity is a cause of T2D, we decided to take advantage of expertise and clinical material of the team of Philippe Gual in Nice (France) (co-author). Fortunately, this team found a correlation between TP53INP1 expression and complications in obese patients. We reported previously that TP53INP1 is over-expressed in many kinds of stress situation, both in vitro and in vivo, including inflammation (Tomasini R et al. JBC 2001). We showed that this over-expression is transient in acute stress and reaches basal level when stress is resolved, which is one major feature of stress proteins. However this over-expression is maintained in chronic stress situations. We further showed that this protective function is lacking in TP53INP1-deficient mice, leading to cell defects observed in chronically stressed cells. Based on this knowledge, we consider that this is what happens in obese patients metabolic tissues (liver in our study), i.e. that TP53INP1 up-regulation is a *response* to the stress of fat accumulation, acting in a *protective* manner. As such, the present study confirms the key role of TP53INP1 as a protective factor in obesity thus T2D promoted by obesity. This role is lacking in TP53INP1-deficient mice which show defects in their response to fat-induced stress. We focused our work on cellular and molecular deciphering of this defective stress response, and provide clues in the role of TP53INP1 in stress resolution by its involvement in autophagy.
In order to improve the manuscript, we made several major changes. First we deleted the figure concerning ALT results (Figure E2D), and we instead show data on a hepatocyte death marker (keratin 18) for which correlation with TP53INP1 expression is much better than ALT. Secondly, we added text in the Introduction and Results sections in order to clarify this issue, in particular by adding some key information from our past studies on the role of TP53INP1 as a key stress protein.

2. The link between loss of TP53INP1 and defective mitophagy/increased mitochondrial numbers requires additional supporting data. First, in addition to staining with mitochondria-specific dyes (mito-Green and mito-Red), the authors need to perform quantitative analysis of mitochondrial DNA and normalize to nuclear DNA. This can be readily done by amplifying mtDNA-specific genes (e.g., COX-I) and nuclear-specific genes. Moreover, co-localization of mitotracker dyes with GFP-LC3 or any other autophagy-specific marker would be more convincing to demonstrate mitophagy processing.

Response: We thank Reviewer #1 for these very pertinent suggestions. We have now performed the required experiments. Regarding mitochondrial DNA quantification, we performed quantitative PCR of mitochondrial genes (COX3 and 12S RNA) and nuclear genes (NDUFV and 18S RNA), and we observed only a slight increase in the ratio mitochondrial/nuclear genes in KO MEFi cells compared to WT. We were thus intrigued by this result since our data obtained by two different techniques (Mitotracker labelling and TEM) convincingly showed a higher number of mitochondria in KO than in WT. We looked at the literature and asked mitochondrial specialists for their interpretation of these findings. Interestingly, mtDNA changes often result from increased PGC-1alpha. Yet, we showed during this revision that PGC-1alpha is *decreased* in KO cells compared to WT (Figure 5A). This result allowed us to propose that mitochondrial biogenesis is likely not increased in TP53INP1 KO cells, thus that mitochondrial accumulation stems from decreased elimination of these organelles. Nevertheless, taken together, those observations are in agreement with impaired mitochondrial homeostasis in the absence of TP53INP1. This issue requires further investigation beyond the scope of this paper but worth addressing (and we have ongoing work in collaboration with Rodrigue Rossignol, Bordeaux, France on this point). Thus we did not add the mtDNA results to the present manuscript in order to avoid introducing complexity instead of required clarity.
Concerning co-localization of mitotracker dyes with LC3, we performed immunocyto-fluorescence experiments followed by careful analysis and quantification of co-localized figures (thanks to a confocal microscope device). The data are completely in accordance with observations made by TEM. We added a supplementary figure (Figure E5) to show those data.

3. Even if there's defective mitophagy, there is no definitive evidence that this is the sole reason for increased ROS. There could also be increased mitochondrial biogenesis in TP53INP1-deficient cells (albeit these mitochondria would have abnormal OXPHOS). The levels of the major mitochondrial biogenesis regulator, PGC-1 should be analyzed as it could a target of repression/degradation by TP53INP1.

Response: This was also a very pertinent suggestion. As mentioned in the answer to issue #2 (above), we analyzed PGC-1alpha by Western blotting experiment and found that it is decreased in KO cells compared to WT (Figure 5A). This result suggest that mitochondrial biogenesis is not increased in TP53INP1 KO cells but rather decreased, thus that mitochondria accumulation stems rather from decreased elimination than increased biogenesis.

4. The in vitro experiments with MEFi showed convincingly that the TP53INP1-/- MEFs have higher basal and high-lipid induced levels of ROS and lipid droplets. This could be due to indirect effects induced during development. Demonstrating that expression of TP53INP1 in trans in these cells can rescue these phenotypes, would provide unequivocal evidence for a more direct involvement of TP53INP1 in this process.

Response: We completely agree that experiments regarding re-expression of TP53INP1 were missing in the initial manuscript. We already reported abolition of TP53INP1-deficiency-associated oxidative stress upon TP53INP1 re-expression (Cano C. et al Cancer Research 2009). We have added this key information in the Introduction section. In the present paper, we report rescue of lipid droplets accumulation upon restoration of TP53INP1 expression (Figure E6). We have added the data of TP53INP1 restoration in the revised manuscript.

5. Why do the male TP53INP1 KO mice exhibit a much more profound phenotype in terms of HFD weight gain that female KO mice? This is never discussed. Also, since the phenotype is more severe in the male mice, why are the TP53INP1 mRNA levels analyzed in female mice in Fig. 3C.

Response: Indeed, we observed that HFD-fed male gain more weight than females as reported elsewhere, as reported by other laboratories (Hwang LL Obesity 2010), probably because sexual hormones impact on metabolism. We have added this information in the revised manuscript (in the Material and Methods section).

Figure 3 gathers important data regarding expression of TP53INP1 in the pancreas as a key organ involved in T2D. These data were obtained by experts of the endocrine pancreas who are co-authors of this study (i.e. AV, GB, GM, PM, MAR, SD, DM, and GAR). Therefore, TP53INP1 expression measured in HFD islets was done independently of our study using TP53INP1-deficient mice and initiated on both genders. We have deleted "female" in the legend of Figure 3E to facilitate the reading. The gender of animals used in Figure 3E is given in the Material and Methods section.

6. In figure 3A, it is unclear what is shown. Is this a picture of a single pancreas from one mouse or beta-cells in culture? In any case, there is no obvious co-localization of TP53INP1(red) and insulin (green).

Response: We have given explanations as well as additional data (IHF on mouse pancreas section) in Figure 3 to clarify this issue.

7. Why are the levels of TP53INP1 much lower in the islets of mice and rats (Fig. 3B and 3C) compared to the exocrine pancreas and other tissues (e.g., spleen?). One would expect that the islets would express higher levels, which the authors report on p 7.

Response: To clarify this issue, which is related to the previous one, we have added IHF on mouse pancreas section in Figure 3, which helps to illustrate that TP53INP1 is expressed in both endocrine and exocrine pancreas. We have improved the presentation of data shown on Figure 3 in the revised manuscript, both in the Results and Discussion sections. Whilst the level of immunoreactivity would indeed appear to be similar in endocrine and exocrine cells we would point out that this is not incompatible with differences in mRNA levels in these compartments, should e.g. mRNA half-life or the efficiency of translation differ between the two.

Minor points:
a. What is "oxygen flow", mentioned on p9 (first paragraph)? From the description of the experiment and the methods section, it appears that the authors are actually measuring oxygen consumption? If that's the case, the term should be corrected.

Response: Yes indeed, we do measure Oxygen consumption. We have corrected the legends in Figure 6.

b. There are several misspellings/grammar errors in the manuscript:
i. P.5: "...histological analysis was more important in KO that in WT mice..." should read: "...histological analysis was more pronounced in KO that in WT mice..."
ii. P.6: "...predisposes them to overweight..." should read "... predisposes them to inceased weight gain...".
iii. P. 12, second par: "Consistently with our previous works" should read ":consistent with our previous work".
iv. P31: "...males mice were submitted to a..." should read "male mice were subjected to...".

Response: We have corrected these errors in the revised manuscript.

Referee #2 (Remarks):

The authors have analyzed in the present manuscript the role of TP53INP1 in the propensity to obesity-related development of type-2 diabetes, with the help of a mouse KO model. The authors convincingly show that TP53INP1-deficient mice are prone to obesity and hepatic steatosis and that this is due to oxidative stress accumulation. They further strongly demonstrate that their KO mice develop type 2 diabetes, proving the value of their model for disease studies. This study represents another successful example of GWASs

usage to unravel disease mechanisms. However, the subsequent mechanistic analysis is rather descriptive (as also reflected in their abstract) and could be improved. How does TP53INP1 prevent oxidative stress? Is mitophagy in general affected or is there a specific effect on the PINK1/PARKIN pathway?

Major comments:
To prove an effect of TP53INP1 in mitophagy, in PINK1/PARKIN levels and in lipid droplet accumulation, rescue experiments by re-expression of the TP53INP1 protein must be performed.
The role of TP53INP1 in mitophagy should be further substantiated and quantified.
To bring an insight into the molecular regulation at game, the authors should investigate the subcellular localization of the TP53INP1 protein, especially in presence and absence of both oxidative stress and lipid stress. Furthermore, the importance of their analysis of TP53INP1 expression in beta-cells (Fig.3) is unclear to this reviewer, because insulin was anyway present in the plasma (Fig. 2B). If, then, they could compare it to the mRNA expression in target tissues, in the presence and absence of HFD and NAC.

Response: We completely agree with this Reviewer that some explanations were missing. Concerning subcellular localization of the TP53INP1 protein, we have added references to our earlier studies demonstrating a nucleo-cytoplasmic localization of TP53INP1. In addition, subcellular fractionation experiments done during the course of this revision showed that TP53INP1 is also detected in the mitochondrial fraction (Figure 5C and Figure E6A) (see also answer to the next issue).
Regarding the TP53INP1 expression in beta-cells issue, we have added IHF on mouse pancreas section in Figure 3, which helps to illustrate that TP53INP1 is expressed in both endocrine and exocrine pancreas. To clarify this issue, we have improved the presentation of data shown on Figure 3 in the revised manuscript, both in the Results and Discussion sections.

The authors could overexpress PINK1 and PARKIN in TP53INP1 KO cells and then look for a possible rescue of their phenotypes. How does TP53INP1 regulate PINK1/PARKIN? Is there a physical interaction? Parkin subcellular localization in wt and TP53INP1 KO cells could be investigated using microscopy immunostaining or WB analysis after subcellular fractionation.

Response: We thank Reviewer #2 for these very pertinent suggestions. We performed immunoprecipitation assays which provided evidence for a direct interaction between TP53INP1 and both PINK1 and PARKIN, but not with BNIP3 or NIX (Figure 5B). Less PINK1 and Parkin are found in the mitochondrial fraction in TP53INP1 KO cells than in WT (Figure 5C). By contrast, re-expression of TP53INP1 rescues the level of PINK1 and Parkin in mitochondria (Figure E6A). These data suggest that physical interaction of TP53INP1 with PINK1 and Parkin impacts on their cell level in particular in mitochondria.
We agree with Reviewer #2 that experiments regarding re-expression of TP53INP1 were missing in the initial manuscript. Altogether, experiments performed in the past and during the course of the revision show that re-expression of TP53INP1 alleviates TP53INP1-deficiency-associated features, i.e. oxidative stress (Cano C. et al Cancer Research 2009), (2) PINK1 and PARKIN decrease in the mitochondrial fraction (Figure E6A), PPARgamma overexpression (Figure E6B), and lipid droplets accumulation (Figure E6C). We have added these important data of TP53INP1 restoration in the revised manuscript (Figure E6).

They observed an impressive and convincing increased mitochondrial mass and ROS production in absence of TP53INP1, effect mimicked, at least in part, by autophagy inhibition (Fig. 4). They then confirm the presence of autophagic vacuoles containing mitochondria in TP53INP1 KO cells, unaffected by H2O2 treatment (Fig. 5A). This brings the authors to conclude that the observed oxidative stress is a consequence and not a cause of impaired mitochondria degradation. However, this assumes that H2O2 treatment equals TP53INP1 KO in terms of oxidative stress and mitochondrial mass effects. Therefore, ROS production and mitochondrial mass should be analysed in wt and KO cells, with or without H2O2 and all 4 conditions with or without 3MA.

Response: We thank Reviewer #2 for this question. Actually, because H2O2 does not affect mitochondrial number (Figure 4), H2O2 treatment does not equal TP53INP1 KO in terms of mitochondrial mass effects. By contrast, H2O2 treatment equals TP53INP1 KO in terms of oxidative stress and lipid droplets

accumulation (Figure 7). These data are in favor of a mitochondrial defect as the cause of the oxidative stress.

Minor comments:
Fig.5A remove red points and rather sign mitochondria without covering them.

Response: We have taken in consideration this suggestion in the revised manuscript.

The subcutaneous fat could also be provided.

Response: Indeed, we did not measure subcutaneous fat during the course of this work. We apologize for this. Actually, investigation on this issue is part of another ongoing project.

The unpublished data mentioned on top of page 7 demonstrating no defects in insulin secretion or islet mass could be included.

Response: We have added these data, mentioned as "data not shown" in the initial manuscript, in a new figure (Figure E3).

Referee #3 (Comments on Novelty/Model System):

The model is of high interest as the authors generate mice deficient in TP53INP1 to dissect its role in metabolic diseases induced by HFD.

Referee #3 (Remarks):

The study of Seillier et al, aims to investigate the role of TP53INP1, a p53 regulated protein with antioxidant properties with tumor suppressive functions, in metabolic syndrome induced by feeding a high fat diet (HFD). For this end authors generate TP53INP1 knockout mice. The data show that KO mice fed HFD gain more body weight, and exhibits more adiposity and hepatomegaly compared to WT mice. KO mice also exhibit worse glucose intolerance and insulin resistance than WT mice and they are also more sensitive to HFD-induced hepatic steatosis than WT mice. All these effects induced by HFD in the KO mice are blunted by NAC administration, while NAC given to WT mice fed HFD does not cause any beneficial effect Mitochondria from KO mice generate more superoxide (mito-sox) and exhibit increased mitochondrial mass compared to WT mice. Work with MEFi indicates that the lack of TP53INP1 increases mitochondria number and size and exhibit decreased oxygen consumption determined by high resolution respirometry, and exhibit reduced Parkin/Pink1 levels, indicative of impaired mitophagy. These findings suggest that in the absence of TP54INP1 mitophagy is impaired contributing to an increase population of dysfunctional mitochondria that generate ROS and this event determines a worse progression towards obesity, fatty liver disease (steatosis) and insulin resistance.

COMMENTS

While the study is of interest and potential relevance, there are a number of concerns that need to be further addressed regarding the mechanisms of TP53INP1 regulation of mitochondrial function and impact in metabolic syndrome.

1. What of the basic questions in to establish is whether TP53INP1 is actually a mitochondrial-targeted protein. Does TP53INP1 regulate mitochondrial respiratory complex assembly and/or function.

Response: We completely agree with Reviewer #3 that these questions were missing in the initial manuscript. We have added in the revised manuscript data showing detection of TP53INP1 in the mitochondrial fraction (Figure 5C and Figure E6A). Regarding mitochondrial respiratory complex

assembly and/or function, we performed analysis of respiratory chain complexes showing decreased activity of complex IV specifically (Figure 6E), without any change in complex expression as quantified by Western blotting (not shown).

2. Is there any defect in the mitochondrial fatty acid b-oxidation capacity in the absence of TP53INP1. Any change in expression or activity of CTP-1.

Response: Again we agree with Reviewer #3 that this question was missing in the initial manuscript. We provide new data showing that decreased oxygen consumption in the setting of lipid substrates (Figure 6C) is probably not related to decreased FAO since levels of CPT1A and CPT1B did not differ between KO MEFi and WT cells (Figure 6D).

3. Does the lack of TP53INP1 regulate mitochondrial antioxidant defenses, e.g. GSH, MnSOD, Prx-III, which could further contribute to the increased ROS examined. Is there also any change in hydrogen peroxide levels?

Response: We have investigated this question of antioxidant defenses in previous studies showing that both enzymatic and small molecules antioxidants are affected (decreased) in the absence of TP53INP1 (N'Guessan P et al. Antioxidants and Redox Signaling 2011). We have added this key information in the Introduction section.

4. The authors do not show that NAC actually decrease the mt ROS determined by mitoSox in Figure 4. This should be established.

Response: This is also an important issue related to the previous one. In our 2011 paper published in Antioxidants and Redox Signaling (N'Guessan P et al.), we showed that NAC alleviates chronic oxidative stress associated with TP53INP1 deficiency through increase of intracellular glutathione level.

5. *Does NAC revert the decrease in Parkin and Pink1 and therefore resumes mitophagy. This is critical to establish cause and effect relationship.*

Response: In the context of the role of TP53INP1, we have of course performed experiments aiming at addressing the link between ROS increase and autophagy impairment in TP53INP1 KO cells. In fact, autophagy is induced by ROS and NAC treatment results in autophagy inhibition (this is what we observed in accordance with data published by other laboratories). In consequence, NAC treatment does not reverse the autophagic block in TP53INP1 KO cells. Those data led us to investigate the hypothesis that autophagic defect in TP53INP1 KO cells is the cause and not the consequence of oxidative stress. We provide in this paper the demonstration of this molecular link.

6. I believe all the work with MEFi are not particularly relevant to the overall aim. I think it would be more important to establish these events in primary mouse hepatocytes instead. Why H2O2 does not potentiate LD in TP53INP1 null cells (Figure 6B).

Response: We agree with Reviewer #3 that data obtained on cells derived from animals were missing. We have now done experiments using primary mouse hepatocytes (reported in Figure 5C). Regarding the second question, we think that LD content in TP53INP1 null cells is so high that further increasing H2O2 level has no impact in these cells.

7. The data on human samples (Fig E2C, D) do not support the findings in the KO mice, and I disagree with such statement made in page 5 (last sentence in first paragraph). To be consistent with the mice data, I would expect lower TP53INP1 levels in samples from patines with steatosis.

Response: This important issue was also raised by Reviewer #1. We added text in the Introduction and Results sections in order to clarify this issue, in particular by adding some key information from our past studies on the role of TP53INP1 as a key stress protein (see answer to Reviewer #1 Major issue #1).

8. Overall, the chronic ROS generation contributing to insulin resistance and obesity is only of relevance in the absence of TP53INP1 not in WT mice. This implies that antioxidants would not be of much value as potential therapy for metabolic syndrome. This issue should be discussed at the end.

Response: We have added this important clinical issue in the Discussion section.

2nd Editorial Decision 18 December 2014

Thank you for the submission of your revised manuscript to EMBO Molecular Medicine. We have now received the enclosed reports from the referees that were asked to re-assess it. As you will see, all reviewers appreciate the improved manuscript and while referee 3 is now fully satisfied, referees 1 and 2 are still concerned about some aspects of the data. Referee 1 is not fully satisfied with the explanation regarding the mouse vs human data discrepancy and request clarifications. Due to the relevance of this part of the work for our scope, I would strongly encourage you to address experimentally this concern as much as you can. Referee 2 suggests to remove the data regarding PINK/PARKIN, as this referee does not find the conclusions well supported by the experiments provided. I would like to suggest to de-emphasize this part of the work, rather than removing the data, and mention the limitations highlighted by referee 2.

In addition, to solve the Western Blot issue mentioned by referee 2, we would encourage you to provide source data, with the aim of making primary data more accessible and transparent to the reader. Would you be willing to provide a PDF file per figure that contains the original, uncropped and unprocessed scans of all or key gels used in the figure? The PDF files should be labeled with the appropriate figure/panel number, and should have molecular weight markers; further annotation may be useful but is not essential. The PDF files will be published online with the article as supplementary "Source Data" files. If you have any questions regarding this just contact me.

In order to move forward following submission of a revised version and accelerate the process, I would mention here a few editorial points that must be addressed too:

1) please provide all expanded view material in the final version (no red font please) as this will not be further edited.

2) reporting statistics: please provide exact p-values and not approximations, and exact n, and number of times the experiments were reproduced.

3) Animal reporting-Please provide the origin of the mice used.

4) Antibodies used, please provide the dilutions at which these antibodies were used.

In order to help you with the sort of information we need for points 1, 2 and 3 follow the author Checklist that you can download from http://embomolmed.embopress.org/authorguide#editorial3
This checklist is to be submitted with all revised manuscripts and up;loaded as an .xls file. Please make sure that all information applicable to your study is detailed in the material and methods, only indicate within the checklist the page number where the information can be found.

We usually expect revised manuscript within two weeks, however as experiments should be performed in this case, I would be grateful if could let us know whether 1 month would be acceptable. I look forward to seeing a revised form of your manuscript as soon as possible.

***** Reviewer's comments *****

Referee #1 (Comments on Novelty/Model System):

There is still some confusion (at least to this reviewer) as to the relationship between the human data and mouse model. (see comments below for additional explanation).

Referee #1 (Remarks):

Carrier et al., have responded in a positive manner to most major criticisms regarding this manuscript. They provide additional data regarding the potential mechanism by which TP53INP1 might be inducing mitophagy and confer protection against ROS in cells and in mice. However, the explanation regarding the apparent discrepancy between the mouse data and human patients is still rather convoluted:

The authors claim that the increased levels of TP53INP1 found in obese patients with steatosis IS evidence that the liver tissue in these patients is stressed (presumably by oxidative stress), and since TP53INP1 is both a regulator of anti-oxidant response AND a stress marker, then these results are consistent with the animal model. However, I don't believe this is the case, at least the way the data is presented. It appears that ANY difference in the levels of TP53INP1 in obese patients with and without steatosis would fit their hypothesis. This suggests that this is not a very meaningful correlation. Is TP53INP1 the only marker of tissue stress? This is unlikely, and perhaps analysis of additional oxidative stress markers would reveal similar correlations.

What is most confusing, though, is the title of the legend in Fig. E2: Verbatim, the authors state: "TP53INP1 predisposes to liver steatosis". This is clearly contrary to the mouse model, (where absence of TP53INP1 predisposes to liver steatosis predisposes to steatosis and TP53INP1 predisposes to liver steatosis protects against ROS in vitro), and remains in the revised version.
The authors should either de-emphasize the human data and remove any conflicting text; or they should analyze the levels of TP53INP1 predisposes to liver steatosis in WT mice fed with HFD and demonstrate a similar correlation with TP53INP1 predisposes to liver steatosis levels.

Referee #2 (Remarks):

In their revised version, the authors made clear with rescue experiments that TP53INP1 affects LD accumulation, depending on PPARgama. This nicely adds to my previous concern on the molecular mechanism involved. However, I suggest to remove the connection to PINK1/PARKIN. In fact, and the point that it might depend on PINK1/PARKIN got even less convincing. Although I see co-IP between TP53INP1 and both PINK1 and PARKIN, the clear decrease in PINK1/PARKIN level in TP53INP1 -/- cells and VDAC increase (5A) is not recapitulated in the mice liver (5C), except partially for PARKIN. Most importantly, also the rescue experiments regarding PINK1/PARKIN are not convincing (E6A). Also, the presence of TP53INP1 in mitochondria is not convincing. In general, the WB images between TCL and mito fractions should not be cut (like in 5C and E6A) but rather several exposures should be presented.

Referee #3 (Comments on Novelty/Model System):

The revised version have addressed substantially all the concerns raised.

Referee #3 (Remarks):

The authors have satisfactorily addressed the concerns raised. No further comments.

2nd Revision - authors' response 02 February 2015

Response to Editor and Reviewers:

Thank you for the submission of your revised manuscript to EMBO Molecular Medicine. We have now received the enclosed reports from the referees that were asked to re-assess it. As you will see, all reviewers appreciate the improved manuscript and while referee 3 is now fully satisfied, referees 1 and 2 are still concerned about some aspects of the data. Referee 1 is not fully satisfied with the explanation regarding the mouse vs human data discrepancy and request clarifications. Due to the relevance of this part of the work for our scope, I would strongly encourage you to address experimentally this concern as much as you can. Referee 2 suggests to remove the data regarding PINK/PARKIN, as this referee does not find the conclusions well supported by the experiments provided. I would like to suggest to de-emphasize this part of the work, rather than removing the data, and mention the limitations highlighted by referee 2.

Response: We warmly thank the Reviewers and Editor for reading our revised manuscript carefully and for their suggestions which enrich our work. We provide responses to the issues raised by the Editor and Reviewers below.
We addressed experimentally the concern raised by Reviewer 1, and added new convincing data in Figure E2 and explained more substantially the issue in the manuscript text. We thank Editor for the suggestion to keep data regarding PINK/PARKIN but de-emphasize this part of this work. For that purpose, we did remove "PINK/PARKIN" in "PINK/PARKIN Mitophagy" text in the Graphical abstract Figure E8. We also added the limitations highlighted by referee 2 in the Results section.

In addition, to solve the Western Blot issue mentioned by referee 2, we would encourage you to provide source data, with the aim of making primary data more accessible and transparent to the reader. Would you be willing to provide a PDF file per figure that contains the original, uncropped and unprocessed scans of all or key gels used in the figure? The PDF files should be labeled with the appropriate figure/panel number, and should have molecular weight markers; further annotation may be useful but is not essential. The PDF files will be published online with the article as supplementary "Source Data" files. If you have any questions regarding this just contact me.

Response: We did prepare a pdf file per figure that contains the original, uncropped and unprocessed scans of gels used in the figure.

In order to move forward following submission of a revised version and accelerate the process, I would mention here a few editorial points that must be addressed too:

1) *please provide all expanded view material in the final version (no red font please) as this will not be further edited.*

Response: We do provide the final version of Expanded Views Material.

2) *reporting statistics: please provide exact p-values and not approximations, and exact n, and number of times the experiments were reproduced.*

Response: We did add the complete statistics information in both manuscript (Figure legends, in blue font) and Experimental Views (in black font, no color font in this section as requested by the Editor in the point 1 above).

3) *Animal reporting-Please provide the origin of the mice used.*

Response: We did add origin of the mice used in the "Mice" paragraph in the Materials and Methods section (in blue font when it is an addition).

4) *Antibodies used, please provide the dilutions at which these antibodies were used.*

Response: We are providing antibodies dilutions in the Materials and Methods section wherever antibodies use is mentioned (in blue font when it is an addition).

***** Reviewer's comments *****

Referee #1 (Comments on Novelty/Model System):

There is still some confusion (at least to this reviewer) as to the relationship between the human data and mouse model. (see comments below for additional explanation).

Referee #1 (Remarks):

Carrier et al., have responded in a positive manner to most major criticisms regarding this manuscript. They provide additional data regarding the potential mechanism by which TP53INP1 might be inducing mitophagy and confer protection against ROS in cells and in mice. However, the explanation regarding the apparent discrepancy between the mouse data and human patients is still rather convoluted:

The authors claim that the increased levels of TP53INP1 found in obese patients with steatosis IS evidence that the liver tissue in these patients is stressed (presumably by oxidative stress), and since TP53INP1 is both a regulator of anti-oxidant response AND a stress marker, then these results are consistent with the animal model. However, I don't believe this is the case, at least the way the data is presented. It appears that ANY difference in the levels of TP53INP1 in obese patients with and without steatosis would fit their hypothesis. This suggests that this is not a very meaningful correlation. Is TP53INP1 the only marker of tissue stress? This is unlikely, and perhaps analysis of additional oxidative stress markers would reveal similar correlations. What is most confusing, though, is the title of the legend in Fig. E2: Verbatim, the authors state: "TP53INP1 predisposes to liver steatosis". This is clearly contrary to the mouse model, (where absence of TP53INP1 predisposes to liver steatosis predisposes to steatosis and TP53INP1 predisposes to liver steatosis protects against ROS in vitro), and remains in the revised version.
The authors should either de-emphasize the human data and remove any conflicting text; or they should analyze the levels of TP53INP1 predisposes to liver steatosis in WT mice fed with HFD and demonstrate a similar correlation with TP53INP1 predisposes to liver steatosis levels.

Response: We thank Reviewer 1 for his/her insistence on this issue which is indeed crucial. We did further address this concern experimentally. First we monitored over-expression of the oxidative stress marker NQO1 in the liver of obese patients cohort (Figure E2G); NQO1 expression was found to be correlated with TP53INP1 expression (Figure E2H) which is itself correlated with steatosis grade (Figure E2F). Secondly we analyzed the expression of TP53INP1 in the liver of HFD-fed C57BL/6J mice and found an almost two-fold increase compared to control-fed mice (done in two different cohorts of HFD-fed mice).

Thus TP53INP1 gene is over-expressed during stress associated with liver steatosis both in mouse and human. We added these new convincing data in Figure E2, we modified accordingly the title of Figure E2 which was unfortunately initially erroneous, and explained more substantially our model in the manuscript text in the result section (in blue font). We also provide below a visual scheme with a simplified working model.

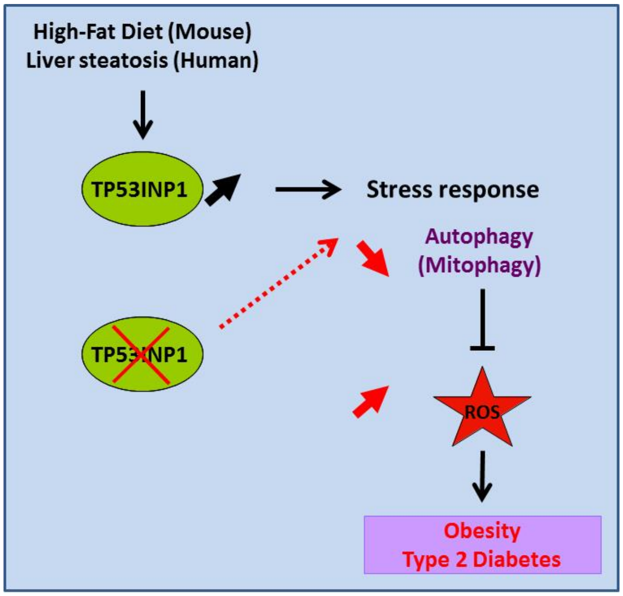

Visual scheme for the issue raised by Rewiever 1

Our laboratory reported previously that TP53INP1 is over-expressed in many kinds of stress situation, both in vitro and in vivo, including inflammation (Tomasini R et al. JBC 2001). We showed that this over-expression is transient in acute stress and reaches basal level when stress is resolved, which is one major feature of stress proteins. However this over-expression is maintained in chronic stress situations. We further showed that this protective function is lacking in TP53INP1-deficient mice, leading to cell defects observed in chronically stressed cells. Based on this knowledge, we consider that this is what happens in obese patients metabolic tissues (liver in our study), i.e. that TP53INP1 up-regulation is a *response* to the stress of fat accumulation, acting in a *protective* manner, as is the case for the antioxidant protein NQO1. As such, the present study confirms the key role of TP53INP1 as a protective factor in obesity thus T2D promoted by obesity. This role is lacking in TP53INP1-deficient mice which show defects in their response to fat-induced stress. We focused our work on cellular and molecular deciphering of this defective stress response, and provide clues in the role of TP53INP1 in stress resolution by its involvement in autophagy.

Referee #2 (Remarks):

In their revised version, the authors made clear with rescue experiments that TP53INP1 affects LD accumulation, depending on PPARgama. This nicely adds to my previous concern on the molecular mechanism involved. However, I suggest to remove the connection to PINK1/PARKIN. In fact, and the point that it might depend on PINK1/PARKIN got even less convincing. Although I see co-IP between TP53INP1 and both PINK1 and PARKIN, the clear decrease in PINK1/PARKIN level in TP53INP1 -/- cells and VDAC increase (5A) is not recapitulated in the mice liver (5C), except partially for PARKIN. Most importantly, also the rescue experiments regarding PINK1/PARKIN are not convincing (E6A). Also, the presence of TP53INP1 in mitochondria is not convincing. In general, the WB images between TCL and mito fractions should not be cut (like in 5C and E6A) but rather several exposures should be presented.

Response: We thank Reviewer 2 for these comments. In accordance with the suggestion of the Editor, we kept data regarding PINK/PARKIN but de-emphasized this part in this work. For that purpose, we did remove "PINK/PARKIN" in "PINK/PARKIN Mitophagy" text in the Graphical abstract Figure E8. Also we modified the text in the Results section (blue font) to mention the limitations highlighted by referee 2. We also modified the title of Figure 5 ("TP53INP1-deficiency is linked with impaired PINK/PARKIN mitophagy" instead of "TP53INP1-deficiency is responsible of impaired PINK/PARKIN mitophagy"). Regarding the rescue experiments (Figure E6A), we show consistent increase of PINK1 when TP53INP1 is over-expressed both in TCL and mitochondrial fraction. By contrast, Parkin expression is low in this experimental setting (U2OS cells) but we made our best to visualize higher level of Parkin in the mitochondrial fraction of TP53INP1-over-expressing cells (4 minutes exposure, see uncropped-unprocessed gels PDF file). Please note that the results are representative of three independent experiments. Finally, regarding the presence of TP53INP1 in mitochondria, this was observed not only on mitochondrial fractions analyzed by WB (Figures 5C and E6A) but also by immunocytofluorescence microscopy (colocalization of fluorescent tagged-TP53INP1 with Mitotracker labeling; data not added into the present manuscript). Thus we totally trust this finding even if we agree that this issue requires further illustration.

Referee #3 (Comments on Novelty/Model System):

The revised version have addressed substantially all the concerns raised.

Referee #3 (Remarks):

The authors have satisfactorily addressed the concerns raised. No further comments..

Response: We thank very much Reviewer 3.

3rd Editorial Decision 04 March 2015

Thank you for submitting your revised manuscript. As you will see from the referee's report below, your article is now ready for acceptance.

***** Reviewer's comments *****

Referee #1 (Remarks):

The authors now provide a reasonable hypothesis and data which clear some of the discrepancies between the mouse and human data in the role of p53 in mitophagy and steatosis. The manuscript makes a significant contribution to the field, and I believe it is suitable for publication in EMBO Mol. Medicine.
