# Supplementary material for: Defects in mitophagy promote redox-driven metabolic syndrome in the absence of TP53INP1
Source: EMBO Mol Med. 2015 Mar 31;7(6):802–18. doi: 10.15252/emmm.201404318 (PMC4459819; doi:10.15252/emmm.201404318)

Uncropped gels used for Figure 5.A

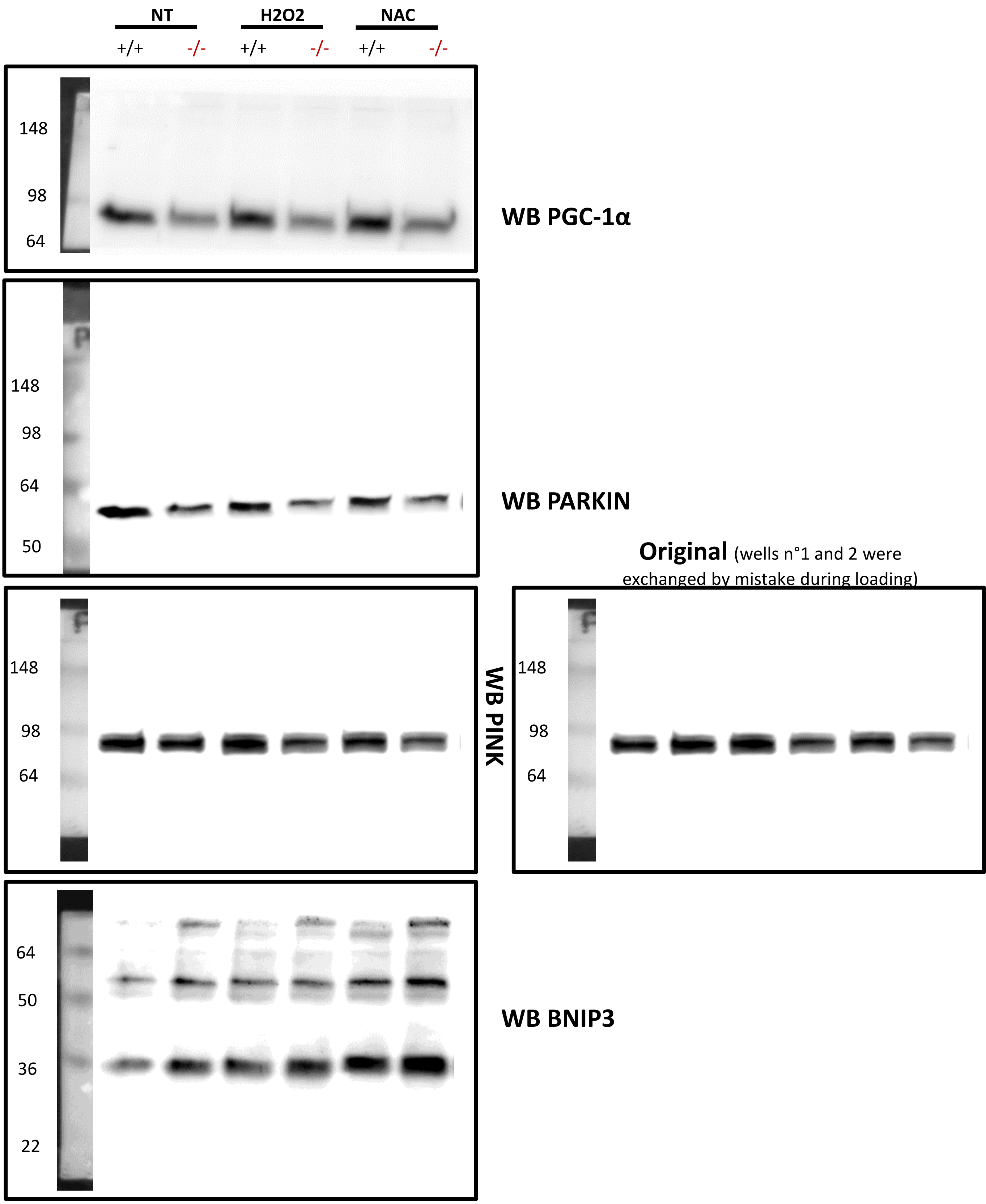

Uncropped gels used for Figure 5.A

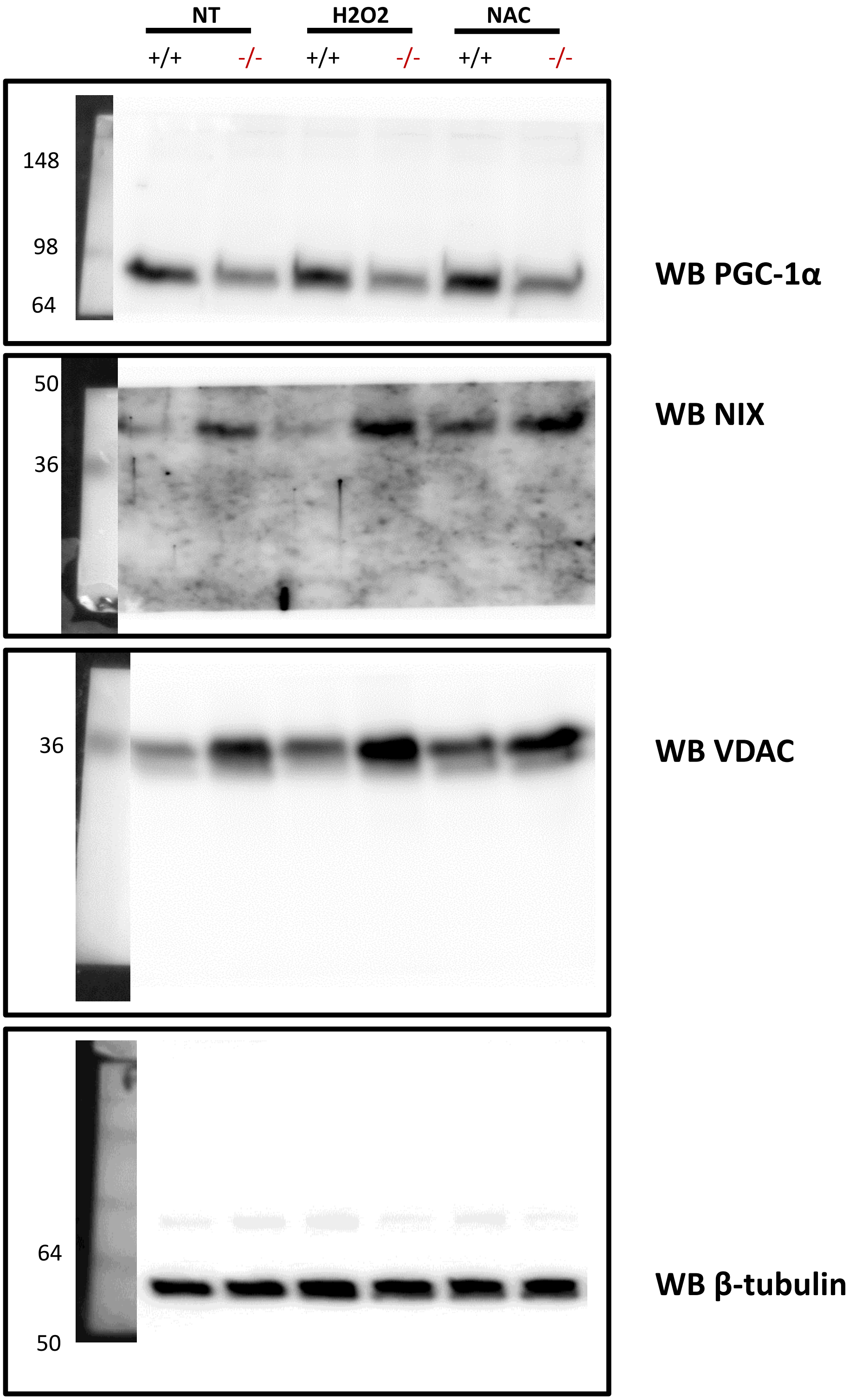

Uncropped gels used for Figure 5.B

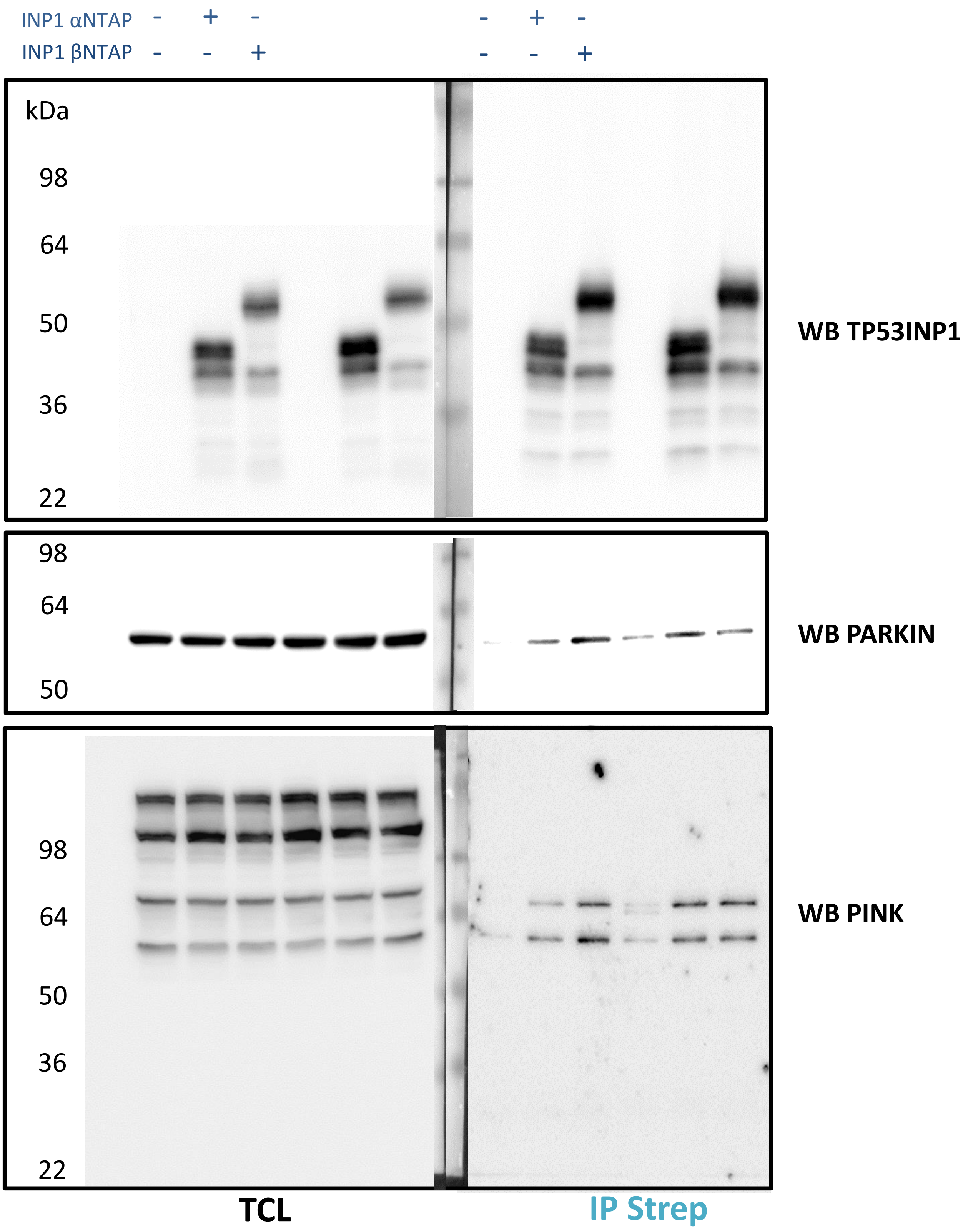

Uncropped gels used for Figure 5.B

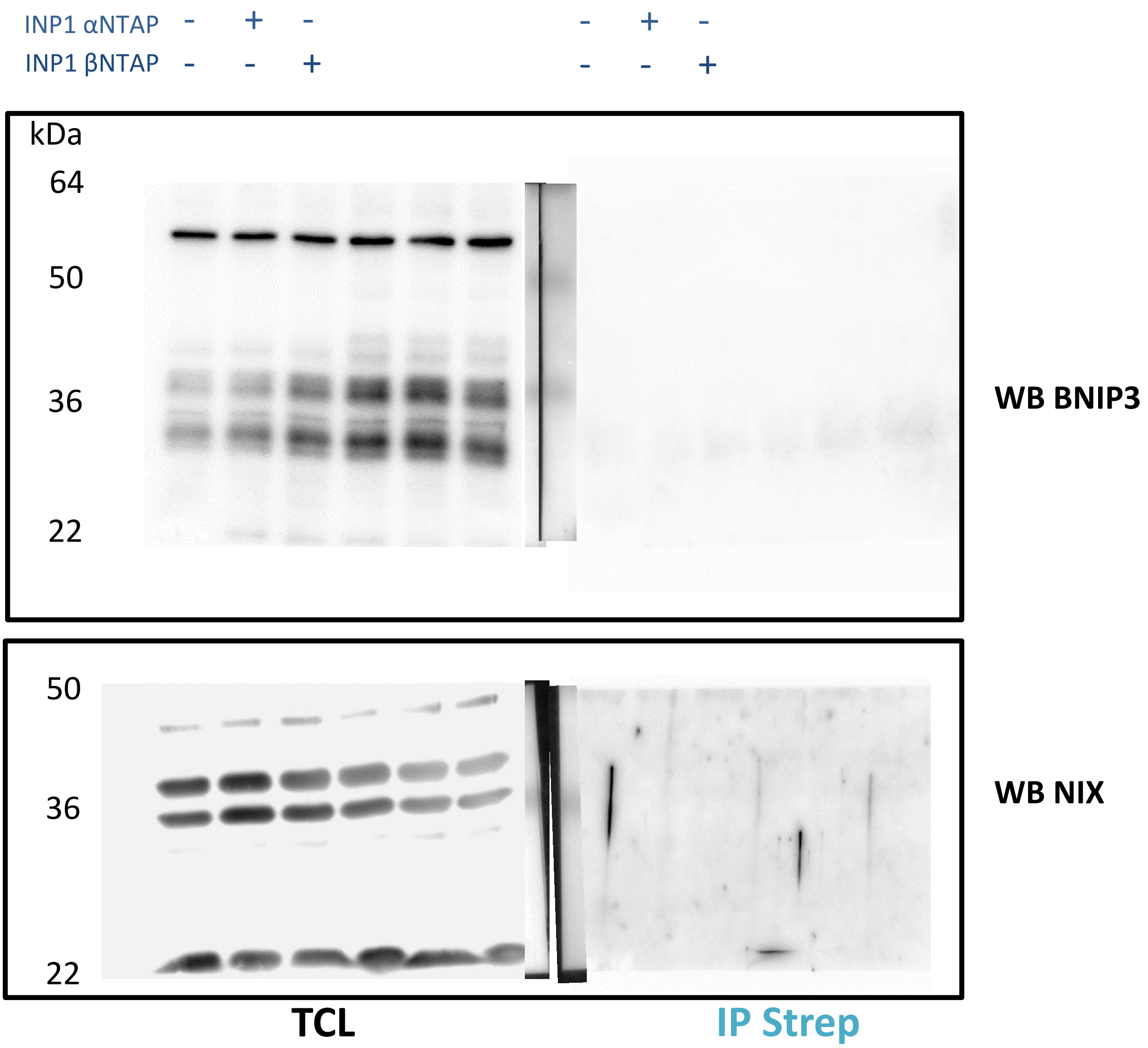

Uncropped gels used for Figure 5.C

TCL and mitochondrial lysates were run on different gels but revealed in the same time

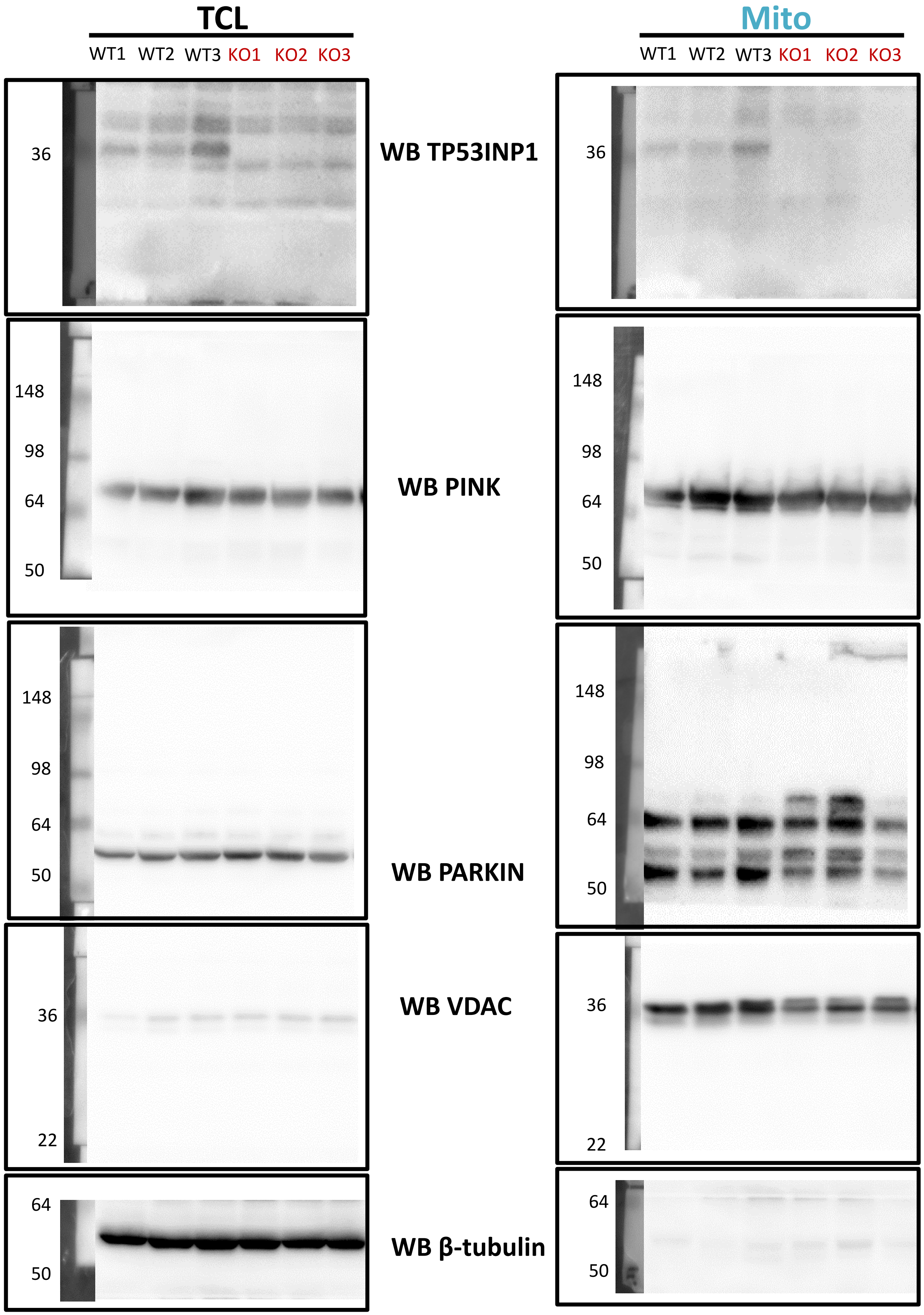

Supplement: Supplementary file 4 [file emmm0007-0802-sd4.pdf]
