# Supplementary figures and images for: Defects in mitophagy promote redox-driven metabolic syndrome in the absence of TP53INP1
Source: EMBO Mol Med. 2015 Mar 31;7(6):802–18. doi: 10.15252/emmm.201404318 (PMC4459819; doi:10.15252/emmm.201404318)

**Uncropped gels used for Figure 6**

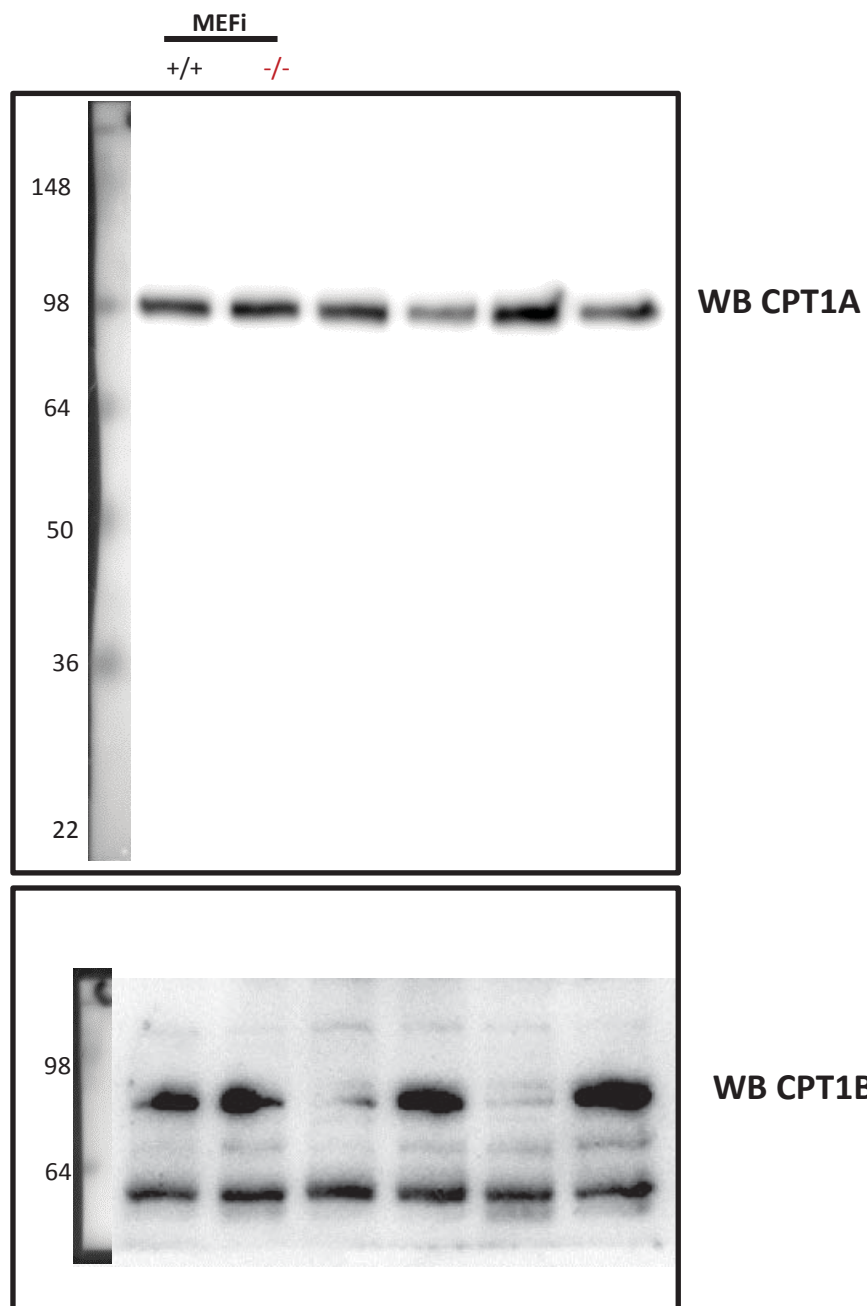

**Uncropped gels used for Figure 6**

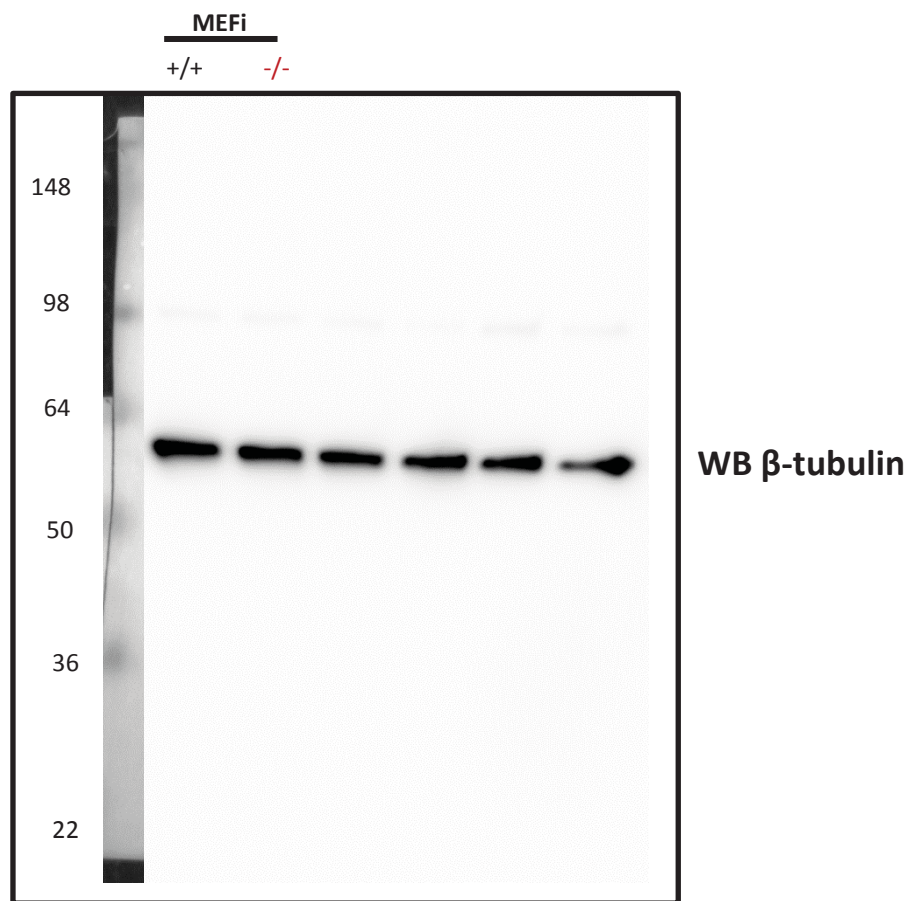

Supplement: Supplementary file 5 [file emmm0007-0802-sd5.pdf]

**Uncropped gels used for Figure 7**

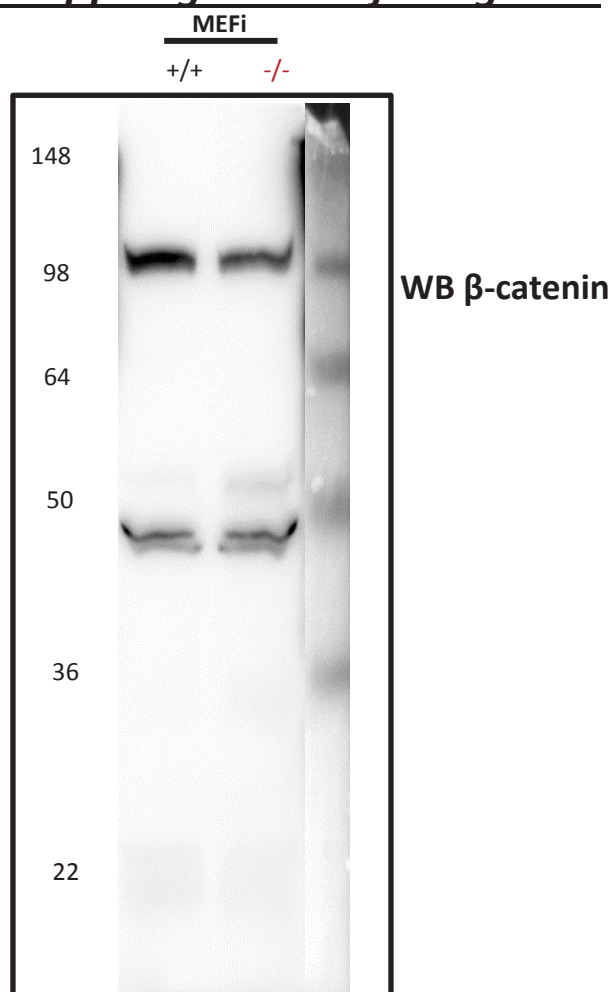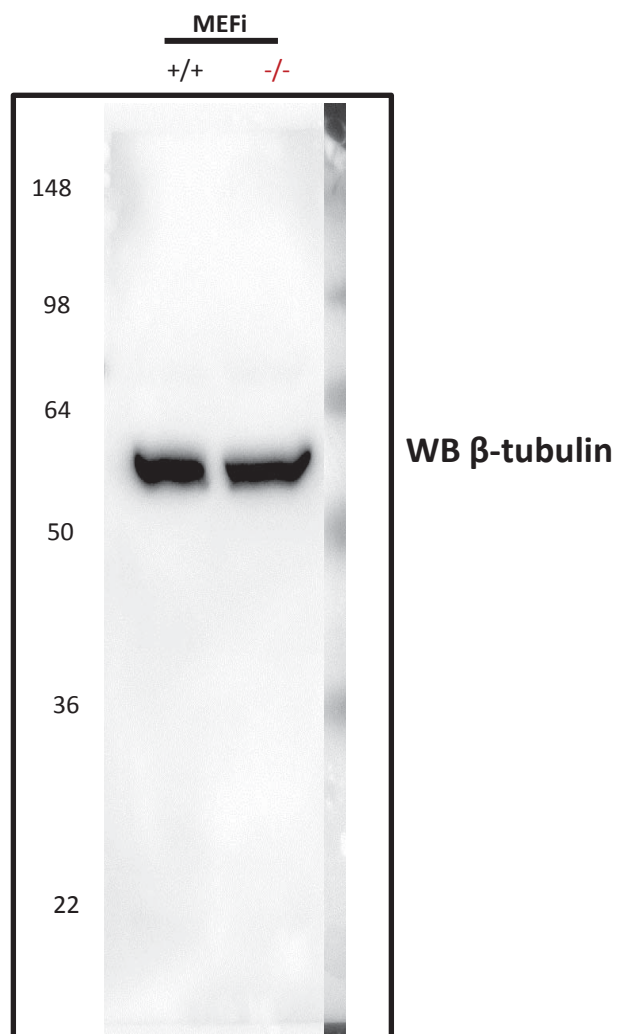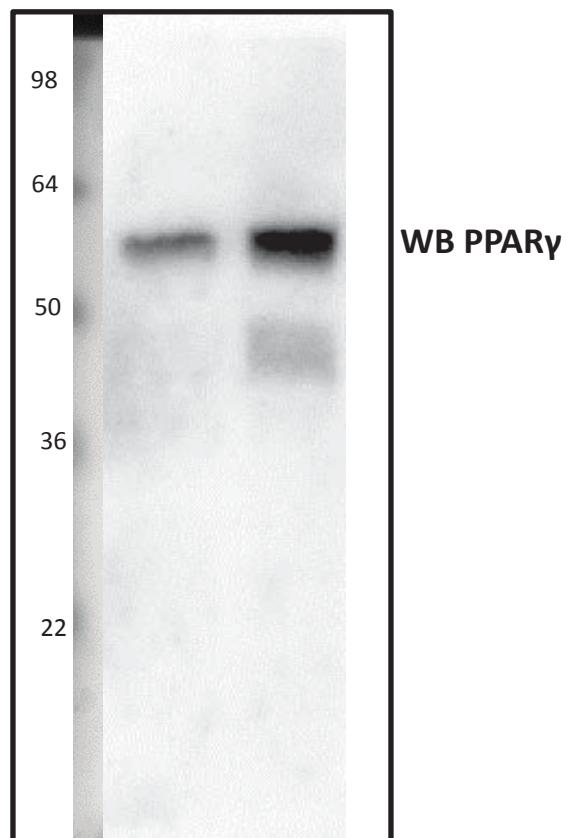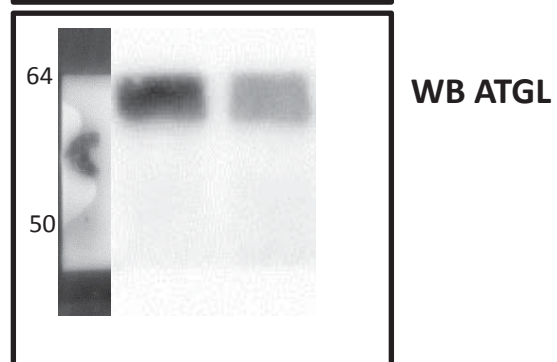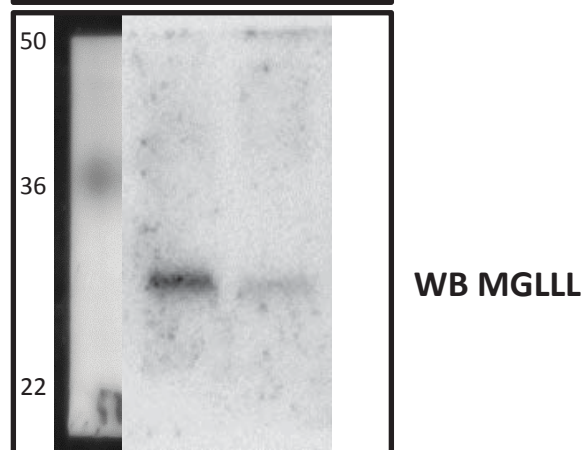

Supplement: Supplementary file 6 [file emmm0007-0802-sd6.pdf]
